# Higher social class is associated with higher contextualized emotion recognition accuracy across cultures

**DOI:** 10.1371/journal.pone.0323552

**Published:** 2025-05-13

**Authors:** Konstantinos Kafetsios, Ursula Hess, Itziar Alonso-Arbiol, Astrid Schütz, Dritjon Gruda, Kelly Campbell, Bin-Bin Chen, Daniel Dostal, Marco J. Held, Petra Hypsova, Shanmukh Kamble, Takuma Kimura, Alexander Kirchner-Häusler, Marina Kyvelea, Stefano Livi, Eugenia Mandal, Dominika Ochnik, Nektarios Papageorgakopoulos, Martin Seitl, Ezgi Sakman, Nebi Sumer, Filip Sulejmanov, Annalisa Theodorou, Ayse K. Uskul

**Affiliations:** 1 School of Psychology, Aristotle University of Thessaloniki, Thessaloniki, Greece; 2 Department of Psychology, Palacký University, Olomouc, Czechia; 3 Department of Psychology, Humboldt University, Berlin, Germany; 4 Psychology Department, University of the Basque Country, San Sebastián, Spain; 5 Psychology Department, Otto-Friedrich-University Bamberg, Bamberg, Germany; 6 Católica Porto Business School, Universidade Católica Portuguesa, Porto, Portugal; 7 National University of Ireland Maynooth, School of Business, Maynooth, Ireland; 8 Psychology Department, California State University, San Bernardino, California, United States of America; 9 Psychology Department, Fudan University, Shanghai, China; 10 Psychology Department, Karnatak University, Dharwad, India; 11 Faculty of Lifelong Learning and Career Studies, Hosei University, Tokyo, Japan; 12 School of Psychology, University of Sussex, Brighton, United Kingdom; 13 Medical School, University of Crete, Rethymno, Greece; 14 Department of Social and Developmental Psychology, Sapienza University of Rome, Rome, Italy; 15 Institute of Psychology, University of Silesia in Katowice, Katowice, Poland; 16 Department of Clinical Psychology, Faculty of Medicine, Academy of Silesia, Katowice, Poland; 17 Polish Academy of Science, Branch in Katowice, Committee of psychological Sciences, Katowice, Poland; 18 Department of Psychology, Bilkent University, Ankara, Turkey; 19 Department of Psychology, Sabanci University, Istanbul, Turkey; University of Glasgow, UNITED KINGDOM OF GREAT BRITAIN AND NORTHERN IRELAND

## Abstract

We tested links between social status and emotion recognition accuracy (ERA) with participants from a diverse array of cultures and a new model and method of ERA, the Assessment of Contextualized Emotion (ACE), which incorporates social context and is linked to different types of social interaction across cultures. Participants from the Czech Republic (Study 1) and from 12 cultural groups in Europe, North America, and Asia (Study 2) completed a short version of the ACE, a self-construal scale, and the MacArthur Subjective Social Status (SSS) scale. In both studies, higher SSS was associated with more accuracy. In Study 2, this relationship was mediated by higher independent self-construal and moderated by countries’ long-term orientation and relational mobility. The findings suggest that the positive association between higher social class and emotion recognition accuracy is due to the use of agentic modes of socio-cognitive reasoning by higher status individuals. This raises new questions regarding the socio-cultural ecologies that afford this relationship.

Emotion Recognition Accuracy (ERA), the accurate perception of emotion displays and emotional states, is a fundamental human ability and key to regulating social relationships and social interaction [1]. ERA helps facilitate and coordinate interpersonal interaction and communication [2] and provides the necessary “affective glue” for interactions between individuals [3,4]. In this research we relate ERA to individual and cultural characteristics that shape social interactions. We focus on social class, a person’s perceived rank in the social hierarchy and their material resources, which is thought to significantly influence people’s everyday social interactions [5,6].

Existing research suggests that ERA capabilities of individuals from higher and lower social status reflect the ways in which they engage with others in their social environment [5,7]. In particular, the social-class-as-culture explanation [8] suggests that people from higher social strata focus more on the self and are less engaged with others, whereas people from lower social strata pay more attention to their social relationships, since social relationships constitute a more critical component for them [9].

These differences in how people of lower or higher social strata engage with others are argued to impact ERA. For example, using several different methodological approaches to ERA, Kraus and colleagues [7] demonstrated an emotion perception advantage for lower-class individuals compared to their higher-class counterparts in North America. In Study 1 participants with higher educational attainment performed worse on a standard test of emotion recognition, the Faces part of the Mayer Salovey Caruso Emotional Intelligence Test (MSCEIT) [10], compared to those with lower education attainment. In a second study, university students who reported lower subjective social status (SSS assessed using the MacArthur subjective social status scale) [11] judged the emotions of an interaction partner more accurately compared to higher SSS students, and in a third study student participants primed with higher social status made less accurate inferences on mental states in the Reading the Mind in the Eyes Test (RMET) [12] compared to those primed with lower social status. An online study in the U.S. also found an inverse relationship between mental state perception as measured with the RMET and social class [13 Study 1], results that were replicated by Schmalor and Heine in another sample recruited in the U.S. [14 Study 2a]. Moreover, recognition of full-bodied expressions of 34 emotional states in the U.S. was more (vs. less) accurate among participants who reported lower (vs. higher) subjective social status using the MacArthur subjective social status scale [15].

Yet, these results and the assumptions behind them have not gone uncontested. For example, Deveney and colleagues [16, Studies 1 and 2] failed to replicate the negative relationship between social class and emotion perception accuracy in RMET and related tasks using subjective [11] and objective social status indicators such as parental income or education level. In Study 3, lower objective social class was even associated with less accuracy in decoding emotions in a multiracial emotion identification task. In a meta-analysis [17] on the role of vertical dimensions of social relations (social power, dominance and social status) and interpersonal accuracy (the ability to make correct inferences about other people’s states and traits), higher social status had a nonsignificant association with ERA but predicted higher interpersonal accuracy overall. There are arguments that higher power, which partially overlaps with social status [8,18], can explain positive associations between social class and emotion perception [19]. In a recent literature review, Schmid Mast and colleagues [20] concluded that holders of positions of power do not appraise others more or less accurately, rather the actual effect depends on the social structuring circumstances; this conclusion was also shared in the Hall et al. [17] meta-analysis. In order to assist readers to fully appreciate existing research on the relation between facets of social class and emotion recognition accuracy we compiled a summary of findings in Table S3.

We identified two problems with the existing literature that has linked social class to emotion recognition accuracy. *First*, all studies in this line of research have used emotion recognition accuracy tasks that focus solely on pattern matching (see [21] for a comprehensive critique of cognitive ERA tasks), and with few exceptions (e.g., the empathic accuracy task), showed faces without social context. As argued [21] a cognitive, pattern-matching, approach essentially changes a social perception task into a cognitive task. Such a task not only takes away the social context within which social perception takes place but, when (as is common) combined with a forced choice answer format, makes it possible to replace emotion recognition with a discrimination or even a guessing task. This bias toward context-free emotion recognition tasks is at odds with the social context-based arguments that form the basis of considering social class a form of culture.

*Second*, most research testing the social-class-as-culture account stems from a single cultural group, which does not provide insight into how observed findings replicate across cultures or how certain aspects of countries’ social and cultural ecologies can shape social class and ERA associations. The scant emphasis on the cross-cultural analysis of the relationship between social class and ERA is particularly odd given that the argument regarding the likely mediating processes between social class and ERA relies heavily on an account of class as culture [22]. We discuss this reasoning in greater detail in the following sections.

## The problem with a decontextualized approach to ERA

Much of the theoretical rationale concerning the psychological effects of social class and in particular the effects of lower versus higher social class on emotion communication is based on describing social class in terms of independent and interdependent self-construal [8], which in turn is strongly linked to cultural background [23]. Specifically, higher class individuals are considered to focus more on the self thereby endorsing an independent self-construal. By contrast, lower class individuals are claimed to pay more attention to their social context and their social relationships [9], which is a defining feature of interdependent self-construal. This difference in motivational priorities when attending to self-versus-others is thought to explain much of the differences found with respect to ERA: lower class persons focus more on others; hence they are more accurate on ERA tasks.

Given this line of argument, however, it is curious that the tasks typically used to assess ERA involved single faces dissociated from their social context. Emotion perception in real life rarely operates devoid of context [24,25]; as such, the context-less nature of the task and the type of information provided are not directly related to the way or ways that emotion perception is practiced in everyday life. The present study addressed some of these shortcomings by adopting the Assessment of Contextualized Emotions (ACE) [26], a new model and method of ERA that has been linked to different types of social interaction outcomes [27,28] as well as correlates of social interaction [29].

The ACE model emphasises the importance of social context when assessing emotion recognition accuracy. By embedding the expresser into a group, the ACE includes the most generic social context (other people) in the emotion decoding process. The term ‘Emotion Recognition Accuracy’ used throughout this MS, refers to how accurately individuals can identify specific emotions from facial expressions. The term, in effect, points to the end-result of the emotion recognition process. The terms ‘Emotion decoding’ or ‘Emotion Decoding Accuracy’ depict the broader process of extracting emotional meaning from facial expressions. ACE distinguishes between emotion decoding accuracy (the signal, the accurate perception of intended emotions, e.g., sadness for a sad expresser), and emotion decoding bias (noise, the perception of other, secondary, emotions that may or may not be actually shown but are perceived, such as anger for a sad expresser).

In order to properly assess the influence of social knowledge on Emotion Recognition Accuracy (ERA), it is insufficient to merely evaluate secondary emotions from single faces. Secondary emotions are more likely to be perceived when observers engage in perspective-taking to understand others, but this process is contingent on the availability of a social context during the perception process. In line with the Truth and Bias model of social perception [30] see also [31,32] ACE accuracy and bias are fundamentally separate processes, with bias being distinguishable from mere error [21]. These two ERA indicators, have unique, measurable and meaningful associations with parameters of everyday social interaction quality [27,28], personal and social well-being [29], personality traits that tap the social domain [33], and neural indicators of perspective taking [34], whereas a standard emotion perception task (MSCEIT-faces) [10] or a standard hit rates assessment of ERA do not (see also [29]).

By embedding ERA within a social context and allowing decoders to assess both accuracy and bias, the Assessment of Contextualized Emotion allows to tap participants’ perspective taking capabilities. Indeed, supporting this idea, recent neuroimaging evidence suggests that an ACE-type emotion task activates brain regions involved in theory of mind, unlike tasks that simply require labelling an emotion expression without context [34].

In addition, culture profoundly impacts emotion recognition accuracy as measured with the ACE. An experimental study conducted with Greek participants [35] found that both chronic and temporarily primed independent self-construal were positively associated with accuracy in the ACE task. A mini meta-analysis of five lab studies using the ACE-cartoons in Greece [33] (Studies 1–5) and a single study utilizing the ACE-faces in Germany [33] (Study 6) also found that higher chronic independent self-construal was associated with higher accuracy. This relationship is primarily understood in social-cognitive terms. Specifically, independent and interdependent self-orientations are respectively associated with less or more attention to context, and therefore with more or less accuracy in the focal emotion recognition task [36,37].

The social class as culture perspective also puts forward expectations as to how social status shapes people’s social cognition. It understands social class as a facet of culture, and the class-specific social environments as affording distinct mind-sets and behavioral scripts unique to each class. Individuals of lower social status tend to adopt a more contextualized cognitive style as expressed by the way they think, their external attributions, expected actions, or other-referent feelings. By contrast, higher social status is associated with a more agentic socio-cognitive engagement with the social world [9]. In several cultural contexts, higher social status individuals tend to adopt more self-orientated, agentic and analytic modes of thinking and feeling [38]. Compared to persons of lower social class, higher social class persons show lower inhibition and higher approach tendencies [39,40], higher self-goals and higher focus on the future [41]. These findings align with evidence that power is linked to a more selective and focused processing style [42]. Schmid Mast et al. [19] reason that this more deliberate processing by individuals high in power is associated with higher interpersonal sensitivity, a concept that includes ERA.

Notably, these different, class associated, patterns of social cognition affect emotion perception, especially when context is provided as well [35,37]. Specifically, higher status, agentic, independent persons are expected to be more focused on the focal target’s emotions and hence would be more accurate in emotion recognition.

## Social class and culture

A second concern motivating the current study was that the existing literature on the social class as culture account has focused very much on the concept of individualism and failed to consider the impact of broader social and cultural ecologies. Typically, research showing that higher SES is associated with higher self-centeredness, and lower other-orientation (such as reduced prosocial behavior; [43]) has emanated from countries higher in individualism. Yet, different cultures may structure the relationship between social class and psychological tendencies, such as focusing on the self and on others, differently [see 44]. Examining the social class – ERA link in different cultures allows to test whether differences in the emphasis people of higher social status place on attending to others versus the self, could translate into differences in decoding other persons’ emotions. As noted above, it is a curious oversight that even though culturally connoted self-other orientations are postulated to explain social class effects on ERA, there is almost no research that spans different cultural and geographical contexts. To our knowledge, only one study considered ethnicity when examining ERA. Monroy et al. (2022) failed to find robust differences among ethnic sub-groups within the U.S.

Therefore, in addition to person and culture-level individualism, Study 2 tested the moderating effect of various cultural factors that are expected to moderate the SSS- ACE relationship. First, we reasoned that one aspect of culture-level influence may have to do with long-term (future) orientation. It has long been argued that social class is positively related to future orientation and delay of gratification [41,45–47]. At the individual level, high-level construal and long-term orientation have been associated with construing emotions in more instrumental, situationally impactful ways [48]. We therefore reasoned that long-term orientation, an expression of future orientation at the collective level [49], would strengthen social class and ERA relationships.

Second, we considered social inequality, after Schmalor and Heine’s [14] reasoning and evidence at the individual level, that perceiving higher economic inequality shifts people’s focus toward the self and hence reduces emotional intelligence abilities especially for those higher in social status.

Last, we assessed cultural differences in relational mobility, the extent to which a given society affords individuals the freedom and the opportunity to form and develop new social and personal relationships [50]. We considered relational mobility as a culture-level behavioral indicator of exposure to social interactions with different persons and relationships. Specifically, limited experiences with different people and new faces can affect accurate recognition of facial emotion expressions [51,52]. Hence, we expected that individuals from cultures with higher relational mobility would exhibit more accuracy (and less bias). We aimed to explore how this aspect of social ecology would play out for the social class –ERA relationship. Since the effect of individualistic orientation related to social class is conceptually situated at the individual and not the cultural level, a culture level effect of individualism was not expected. We nevertheless exploratively tested the effect of specific cultural level indicators.

## The present studies

To address the above concerns and research gaps we examined the relationships between social class and emotion recognition accuracy in a diverse array of cultures using an ERA method that incorporates social context. Study 1 was conducted in the Czech Republic and involved a community sample and Study 2 was conducted in 12 cultures that span three continents: North America (USA), North, Central and South-East Europe (Ireland, UK, Italy, Germany, Greece, Turkey, Poland, and Spain) and South and East Asia (Japan, China, India). The countries were selected with the aim to have a good mix of cultures from different parts of the world in terms of key cultural orientations, such as variation in individualism/collectivism [53] or honor/face/dignity [54].

We used both a subjective [11] and an objective indicator of social class (Parental Educational Attainment Level), because social class contexts are considered as being influenced by two processes: the construction of an individual’s rank relative to others in the social class hierarchy and the objective experience of the different levels of material resources that define an individual’s social live [8]. In this vein, parental status is frequently employed as a proxy for gauging the socioeconomic background of university/college students [55].

We predicted that - due to the agentic mind set of higher social class persons - higher social class would be associated with more accuracy (and less bias) in the ACE task and this association will be explained by the established [38] individualistic self-construal of higher-class persons. A central question within the scope of these studies was whether a traditional hit rates approach - associating one (correct) label to a single emotion expression - can provide the same information as the accuracy and bias approach of the ACE. This is important given that most of the studies on the topic have adopted such a traditional hit rates approach to assess ERA. Across all studies, the assessment of accuracy and bias in a contextualized assessment of emotion was expected to be superior to simple hit rates in revealing associations with personality traits. Finally, Study 2 tested the moderating effect of cultural factors that are known to moderate the SSS - ACE relationship: Long Term Orientation, Relational mobility and Inequality.

## Study 1

Study 1 constituted a first test of the main hypothesis that higher social class is associated with higher accuracy when an assessment of contextualized emotion is used but not when using a traditional hit rates approach, which associates one (correct) label to an emotion perception.

## Method

### Participants and procedure

Participants (*N* = 338) were between 18 and 35 years (*M* = 23.24, *SD* = 4.92), of whom 241 self-identified as women 97 as men. From the total sample of 384, 44 did not complete all the necessary information and 2 were outside the targeted age range. These cases were excluded. They were recruited using non-probability sampling from the general population of the Czech Republic via advertisements on social media platforms, in local newspapers and posters displayed in public areas. Data collection started on 5^th^ January 2023 and ended on 17th December 2023. The procedure involved different phases of data collection (online and in the laboratory, see detailed description in the Supplementary File S1). Participants provided informed consent in writing. Here we present results from the main online data collection. A power analysis performed using G*Power statistical software [56] showed that a minimum of 273 participants was required to achieve a statistical power of 80%, assuming a small effect size (*r* = .15), a Type I error rate of 5%, and a one-tailed test of significance. The data presented here are part of a larger pre-registered project (https://osf.io/pu56h/?view_only=3fbcf8042 fa34383b68dc436ae404dd9). However, the hypotheses of the present study were not preregistered. The research project was approved by the research ethics committee of the Palacký University.

### Measures

All measures were translated into Czech using a team-based translation approach [57].

#### Subjective and objective social class.

Social class was operationalized in terms of subjective social status (SSS) using the MacArthur Scale of Subjective Socioeconomic Status [11], a measure that depicts a ladder with 10 rungs, which participants use to indicate their relative standing in society. Second, we assessed participants’ parental educational status on a six-point scale (mother and father) ranging from ‘no formal education’ to “completed a post-graduate university degree.” Parental education status is considered a measure of objective SES and a reliable indicator of social class used in past research [9].

#### Emotion recognition accuracy.

**Fig 1:**
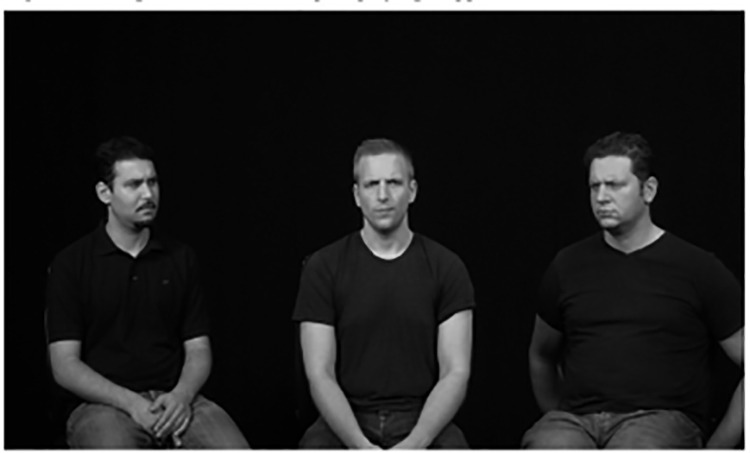
Example of ACE –Faces. *Note:* Target depicts emotion of anger, with surrounding persons expressing a congruent emotion. For copyright reasons only an example image is provided. Interested authors can ask for permission to use the material.

We used a brief version of the Assessment of Contextualized Emotions [see 33] to assess ERA. Participants saw a series of 24 images (see Fig 1 for an example) showing four emotions (sad, happy, angry, disgust) expressed by one man or one woman, who was shown either alone or surrounded by two other individuals of the same gender who showed either the same or a neutral expression. There are 16 different pictures (8 male) depicting emotion in context and 8 different pictures depicting emotion in single faces. Stimuli were selected from the ACE-full [27] as those showing higher test-retest reliability based on previous research [33 study 7]. By embedding the expresser together with others, the ACE includes the most generic social context (other people) in the emotion decoding process. Participants’ task was to rate, on a seven-point scale, the central person’s emotion expression using seven emotion scales (calm, fear, anger, surprise, disgust, sad, happy). The scoring dimensions of the ACE are accuracy (the intensity rated for the target emotion scale, i.e., anger intensity on the anger scale for an angry expression) and bias (the mean of ratings on all non-target scales) which have been shown to constitute two distinct facets of emotion decoding [27,33]. Accuracy is based on an intensity judgment because intensity has been shown to be a valid and meaningful indicator of ERA [58]. Specifically, ACE accuracy pertains to the ability to accurately perceive the emotional intensity expressed by the focal individual whereas ACE bias accounts to perceiving intensity of emotions additional to those communicated by the focal person.

Hit rates were calculated on the same ratings such that 1 was scored when the target scale (i.e., anger for an anger expression) was rated higher than any other scale, 0 when this was not the case. Accuracy, bias scores and hit rates, were calculated separately for each emotion and later combined. In line with the truth and bias model of social perception [30], ACE accuracy and bias are two dimensions which – event though theoretically independent – are typically correlated (see [33], average *r*(338) =.472 in the present sample); therefore, when predicting ACE accuracy or bias, it is imperative to account for the respective other variable.

***Individualism and collectivism*** were measured with the 24 item Singelis individualism and collectivism scale assessing the horizontal and vertical dimensions of individualism and collectivism [59]. Example items: for horizontal individualism (e.g., “I often do my own thing”; “I am a unique individual”); for vertical individualism (e.g., “It annoys me when other people perform better than I do”; “Competition is the law of nature”); for horizontal collectivism (e.g., “The well-being of my coworkers is important to me”; “To me, pleasure is spending time with others”); and for vertical collectivism (e.g., “I would sacrifice an activity that I enjoy very much if my family did not approve of it”; “I usually sacrifice my self-interest for the benefit of my group”).

***The Faces branch of the Mayer, Salovey, Caruso EI Test*** (MSCEIT) [10] includes photographs of faces. Participants are instructed to assess how much a certain emotion (given a list of five emotions) is expressed in each of the photographs (from 1 = not at all present to 5 = very much present). Scoring of the items utilizes the consensus method which indicate the extent to which responses align with the established norms for this specific sample [10].

## Results and short discussion

Subjective social status (SSS) was significantly and positively related to ACE accuracy and bias (*r* (338) =.160, *p* = .003, and *r*(338) =.147, *p* = .007) but not to either hit rates accuracy (*r* (338) = -.067, p = .218) or the MSCEIT faces scores (*r* (338) = -.032, p = .551.05 see Table S1). SSS was also significantly and positively related to vertical individualism (*r*(338) =.180, *p* = .001). To test the effect of social status on ERA accuracy and bias we regressed ACE accuracy on SSS controlling for gender and ACE bias. SSS was a significant predictor of ACE accuracy (β = .102, *t* = 2.13, *p* = .034, total *F*(4,334) = 37.87, *p* < .001 See Table S2). When, in a second step, vertical individualism was entered in the equation, the SSS and ACE accuracy relationship was rendered nonsignificant (β = .095, *t *= 1.95, *p* = .052). Regressing ACE bias on SSS (also controlling for gender and ACE accuracy) did not yield any significant relationship of SSS with ACE bias (β = .057, *t* = 1.19, *p* = .235). There were no significant gender by SSS interaction effects either on ACE accuracy or ACE bias and SSS was not related to ACE bias. Parental education level, an objective index of social class, was not significantly or meaningfully related to either ACE accuracy (*r* (338) =.033, p = .549) or bias (*r* (338) =.072, p = .186, see Table S1). We also explored the relation of SSS with emotion recognition accuracy of faces without context (single faces) and the relationship was positive but nonsignificant (β = .086, *t* = 1.7, *p* = .078 see Table S2).

Overall, the results tend to support the hypothesis that higher social class is associated with higher emotion recognition accuracy in context. There was also initial evidence pointing to the role of person-level individualism for explaining this relationship. However, these results came from a single cultural group known to be relatively high on horizontal collectivism [60], therefore, Study 2 tested the hypotheses using a broader sample of cultural groups.

## Study 2

Study 2 tested the main hypothesis in 12 cultures across the globe. A broad sample set allowed to also test ERA effects at the collective level and in a more reliable way than in the past. This broad sample of cultural groups from different regions of the world allowed us to explore, for the first time, socio-ecological factors that may affect the social class –ERA relationships, beyond independent and interdependent self-construal: future orientation, relational mobility, and economic inequality.

## Metvhod

### Measures

The study was completed in the official language of each respective country. The data collection period started on 13^th^ July 2020 and ended on 1^st^ July 2021 for all sites (see Supplementary File S1 for specific period dates for each site data collection). Questionnaires and instructions were compiled in English and then translated into other study languages following a team translation approach [57]. All scales and tasks were first translated by native speakers and then checked by a team member (fluent in both English and the local language) to ensure they were understandable, meaningful, familiar, and appropriate for the respective cultural context. The data presented here are part of a larger pre-registered study (https://osf.io/pk3mu) testing hypotheses on cultural differences in attachment and self-other orientation. However, the present study hypotheses were not preregistered.

Given the lack of prior research on this topic, we followed previous multisite cross-cultural studies that included cognitive predictors similar to those in our study [e.g., 61]. An a priori power analysis [56], based on a small to medium effect size of *f*² = 0.07, with a power target of 0.80 and an alpha level of 0.05, suggested a required sample size of 141. All sites significantly surpassed this number (see Table 1 for a detailed breakdown of data collection and sample demographics). The final dataset comprised 2,440 participants (1,653 women, 775 men, and 12 non-binary individuals) with an average age of 24.4 years (*SD* = 7.81). Following data collection, we performed a multilevel power analysis using the simr package in R [62], which confirmed that the sample size was sufficient to detect medium Level 1 effects, with statistical power of 86.10%, CI_95%_ [83.80%, 88.19%].

**Table 1:** Main descriptives for each data collection site.

Country	*N*	Language	% Fem.	Age		SSS		Accuracy		Bias		Hit rates		Acc Bias	Gini	IND	LTO	RM
				*M*	*SD*	*M*	SD	*M*	*SD*	*M*	*SD*	*M*	*SD*	*r*				
**China**	211	Chinese	66.80	19.84	1.75	5.86	1.47	2.89	0.52	1.83	0.41	.30	.14	0.47	46.80	20	87	4.04
**Spain**	181	Spanish	54.70	28.94	4.31	5.97	1.31	3.47	0.56	1.82	0.43	.43	.17	0.07	34.30	51	48	4.42
**Germany**	211	German	82.90	25.63	9.34	6.34	1.58	3.51	0.54	1.72	0.36	.51	.17	0.32	32.00	67	83	4.19
**Greece**	215	Greek	76.30	27.51	9.16	5.74	1.51	3.46	0.54	1.78	0.38	.44	.16	0.29	33.20	35	45	4.10
**India**	154	English	61.70	29.22	9.78	6.39	1.88	3.37	0.59	2.12	0.54	.28	.16	0.20	37.80	48	51	3.20
**Ireland**	146	English	67.10	27.89	10.69	6.09	1.47	3.40	0.52	1.86	0.41	.38	.17	0.11	30.30	70	24	4.29
**Italy**	308	Italian	69.50	23.80	7.27	6.11	1.34	3.26	0.53	1.72	0.33	.42	.17	0.30	35.40	76	61	3.95
**Japan**	187	Japanese	54.00	20.42	3.16	6.54	1.30	3.27	0.57	2.20	0.44	.25	.14	0.41	32.10	46	88	3.93
**Poland**	193	Polish	65.80	22.90	4.56	5.55	1.39	3.33	0.57	1.71	0.33	.45	.16	0.18	31.80	60	38	4.42
**Turkey**	207	Turkish	68.60	20.30	2.14	6.64	1.31	3.43	0.59	1.82	0.33	.43	.15	0.33	39.80	37	46	4.12
**USA**	229	English	56.80	28.99	9.44	5.11	1.72	3.38	0.55	1.73	0.38	.40	.17	0.35	41.50	91	26	4.38
**UK**	198	English	84.30	19.31	2.37	5.26	1.43	3.52	0.52	1.75	0.34	.44	.16	0.17	32.50	89	51	4.32
																		
**Total**	2,440		67.70	24.41	7.81	5.97	1.47	3.35	0.57	1.82	0.41	0.40	.17	0.33				

*Note:* SSS: Subjective socio-economic status, IND: Individualism, LTO: Long Term Orientation, RM: Relational mobility

### Transparency and openness

All measures, manipulations, and exclusions in these studies are disclosed in the following sections and the supplementary File S1. All data are publicly available (https://osf.io/vz6jy/?view_only=fa31c2de9c7c4b10bd0df66a1dfa5a06).

### Participants and procedure

Participants completed a web-based survey on “people’s social relationships and cognitive styles” using two online platforms (Qualtrics for the US and the UK samples and LimeSurvey for all other samples). The survey was distributed to student participants, who were over the age of 18, from universities across twelve countries (China, Germany, Greece, India, Ireland, Italy, Japan, Poland, Spain, Turkey, the UK, and the US). Completion of the survey took approximately 35 minutes (*M* = 35.6, *SD* = 15.5). Participants with extreme values in completion time (i.e., under 10 min.) and those who failed attention checks were excluded; these exclusion criteria reduced the final sample to *N* = 2440 from *N* = 2618. Five participants were excluded for being under the age of 18, 12 for missing values and outliers, and the rest for failing an attention check or having extreme values of completion time. The survey was anonymous, participants could stop at any time, and confidentiality of information was assured. At the beginning of the survey, participants were informed that completing it constituted providing informed consent. The study was approved by the ethics research committee of University of Crete and collaborating Universities which required additional ethics approval. The average age of participants was 24 years (*M = *24.4, *SD* = 7.81). Participants self-identified as female (*n* = 1653) or male (*n* = 775) and 12 participants identified themselves as non-binary. Further, 41% of the participants had a high school diploma, 40% had a university degree, and 18% had a postgraduate degree. Please refer to Table 1 for details on data collection and final sample descriptive statistics. The same measures of **subjective and objective social class** were utilized as in Study 1.

#### Emotion recognition accuracy.

We used a brief version of the Assessment of Contextualized Emotions (modelled after ACE-brief, [33]) to assess ERA. Participants saw a series of 20 images (see Fig 1 for an example) showing four emotions (sad, happy, angry, disgust) expressed by a man and a woman, who were depicted either alone or surrounded by two other individuals of the same gender who showed either the same (congruent) or a neutral expression (incongruent). There are 18 different pictures (9 men) depicting emotion in context (9 incongruent) and 2 pictures (1 man, 1 woman) depicting a single person showing a happy expression. Stimuli were selected from the ACE-full [27] based on their high test-retest reliability as assessed in previous research (see [33], study 7). Decoders used a seven-point scale, to assess the (central) person’s emotion expression in terms of seven emotions: calm, fear, anger, surprise, disgust, sad, happy. Accuracy, Bias and hit rates were calculated as in Study 1. Accuracy and Bias were correlated *r*(2440) =.33 in the present samples. The main test characteristics are presented in Table 1.

#### Self-construal.

We measured self-construal using the revised version of the Self-Construal Scale (SCS) [63]. The scale has two orthogonal dimensions measuring the strength of independent and interdependent self-construal. The independent self-construal subscale contains items that assess uniqueness in social behavior and related cognitions and emotions (e.g., “I do my own thing, regardless of what others think”). The *interdependent* self-construal subscale includes items that assess connectedness in social behavior, specifically emotions, cognitions, and behaviors regarding in-groups (e.g., “It is important to me to respect decisions made by the group”). Each subscale contains 15 items, which were all rated using a 7-point Likert scale (1 = *strongly disagree* to 7 = *strongly agree*). Results for internal consistency and cross-cultural construct equivalence of independent and interdependent self-construal are presented in the supplementary file (Table S1).

Long term orientation indicators for each country were taken from Hofstede [53]. Gini indices were retrieved from the World Bank Data (https://databank.worldbank.org/ home.aspx) with 2019 as the index year. If a value for a country was not available for 2019 the closest values available to that year was used. For relational mobility we utilized the raw country indicators reported in Thomson et al. [50] available for nine of the 12 countries in our dataset and conducted three new, independent online studies in India, Italy and Greece (see Supplementary File S1).

## Results

The data from the present study constituted a nested data structure in which ratings were analysed as nested within people using a series of multilevel random coefficient models. We conceptualized the data as having a two-level hierarchically nested data structure within countries and analysed these data using the program HLM [64] following guidelines offered by Nezlek [65]. First, we calculated unconditional models (no predictors at either level of analysis) that estimated the means and variances (within- and between- persons) of the measures of ACE accuracy and bias. Summaries of these analyses are presented in Table 2. Inspection of the means suggests that overall, participants were more accurate than biased in ERA and much of the measures’ variance was within countries.

**Table 2:** Zero order correlations and descriptive statistics for study variables.

	1	2	3	4	5	6	7
**1. Accuracy**		.334^**^	.283^**^	.052^**^	.014	.087^**^	.070^**^
**2. Bias**			-.572^**^	.076^**^	-.063^**^	.016	.080^**^
**3. Hit rate**				-.011	.043^*^	-.025	-.043^*^
**4. SSS**					.063^**^	.135^**^	.030
**5. Parental education**						-.001	-.002
**6. Independent**							.014
**7. Interdependent**							
**Mean**	3.36	1.84	0.39	5.97	7.24	4.78	4.65
**SD**	0.05	0.04	0.02	0.14	0.23	0.05	0.04
**Within-countries Variance**	91%	86%	82%	90.05%	89%	92.5%	95%

*Note: N* = 2,440, SSS = Subjective social status. The correlations among the study variables are presented to be consistent with previous research. Such correlations confound between- and within-group variability and therefore provide potentially inaccurate descriptions of relationships at the individual level. * *p *< .05, ** *p *< .01.

Residual versus predicted values plots of ACE accuracy and bias (see Figs S1 and S2) suggested the model was homoscedastic and that residuals versus predicted values relationships were not systemically invariant. We present the proportion of variance within persons (the converse of intraclass correlation [ICC]) as also indicative of power for detecting cross-level interaction effects. Lower ICCs favour the power for social interaction direct effects, whereas higher ICCs favour the power for cross-level direct effects [66]. While an extensive discussion on the ideal sample size for multilevel modeling is outside the scope of this paper, it is worth noting that there are varying recommendations among researchers. Some researchers contend that even with as few as 10 groups, utilizing random effects rather than fixed effects is appropriate [67]; see Quesque et al. [68] for such a limited number of randomly varied country predictors on the topic of emotion perception.

The main hypotheses involved relationships between social class (subjective and objective) and ACE accuracy and bias at the individual level with no predictors at the culture level. These analyses also controlled for gender and age given known interrelationships between those and ERA [69,70]. The 12 cases indicating gender ‘non-binary’ were randomly assigned 1 (male) for the current analyses. This coding did not result in any difference in the relationship between Gender and ACE accuracy and bias compared to the initial coding. Results from control MLM analyses performed excluding these 12 cases did not meaningfully differ from those reported here (see Table S16). The model predicting ACE accuracy is presented in (1). Given that some of the samples were collected after the start of the Sars-Cov-2 epidemic and in order to control for possible COVID related behavioral effects we also entered time elapsed since Covid started as a control variable (see Table S17) in the main analyses, but there were no significant or meaningful differences in the results from those in the reported analyses. All variables were group (country)-mean centered, except for gender which was entered uncentered, and all predictors were modelled as randomly varying across countries.

(1) **Level 1:** y_ij_ = β_0j_ + β_1j_*SSS + β_2j_*Gender + β_3j_*Age +β_4j_*Bias +r_ij_.
**Level 2:** β_0j_ = γ_00_ + u_0j_
      β_1j_ = γ_10_ + u_1j_
      β_2j_ = γ_20_ + u_2j_
      β_3j_ = γ_30_ + u_3j_
      β_4j_ = γ_40_ + u_4j_

Subjective social status (SSS) was positively associated with ACE accuracy (γ_01_ = .017, *t *= 2.22, *p* = .048); gender (women scored higher than men and nonbinary persons, γ_02_ = .15, *t* = 3.07, *p *= .011,) and higher age (γ_03_ = -.005, *t* = -3.35, *p* = .006) were also significant predictors of ACE accuracy. SSS was also significantly related with ACE accuracy without the gender and age controls (γ_01_ = .018, *t *= 2.33, *p* = .040). SSS was not a significant predictor of ACE bias (γ_01_ = .001, *t* = .02, *p* = .985) and gender was inversely related to ACE bias (γ_02_ = -.12, *t* = -6.02, *p* < .001). We calculated the effect size of the full model compared to a null model [71] to be *f*^2^ = .0.

Using the same analytic scheme as depicted above, we replaced SSS with parental education level, an objective index of social status. Parental education level was not significantly associated with ACE accuracy (γ_01_ = .017, *t* = 1.65, *p* = .127), yet, was negatively associated with ACE bias (γ_01_ = -.019, *t* = -5.16, *p* < .001) a relationship also depicted in Fig S4. We calculated the effect size of the full model compared to a null model [71] to be *f*^2^ =.03.

Independent self-construal was added to the model in a second step to test the prediction that chronic independence mediates the relationship between SSS and accuracy. As expected, independent self-construal was positively associated with accuracy (γ_05_ = .042, *t* = 2.73, *p* = .020) and its inclusion rendered the SSS and ACE accuracy relationships nonsignificant (γ_01_ = .013, *t* = 1.87, *p* = .089). A separate analysis, with independent self-construal as the outcome variable, found SSS positively associated with independence (γ_01_ = .072, *t* = 6.68, *p* < .001) but not with interdependence (γ_01_ = .002, *t* = 0.28, *p* = .780). These findings suggest that the positive relationship between SSS and ACE accuracy was primarily driven by positive associations between SSS and independent self-construal. By contrast, entering independence or interdependence, did not meaningfully change relationships between parental education level and ACE bias.

### Across and within country assessment of hit rates

A separate set of analyses using subjective social status/parental educational level, gender and age but this time with hit rates as the outcome variable did not find a significant relationship between SSS and hit rates (γ_01_ = .002, *t* = 1.13, *p* = .280). Parental education had a marginally nonsignificant positive association with hit rates (γ_01_ = .002, *t* = 2.03, *p* = .067). Hit rates were significantly associated with gender (women had higher hit rate accuracy γ_02_ = .026, *t* = 2.61, *p* = .024) and age (older persons had lower hit rate accuracy γ_03_ = -.001, *t* = -4.06, *p* < .001).

Given that most of the existing research took place with North American samples, we also examined the main association between subjective social status and ACE accuracy/bias and hit rates with only our sample from the US (see Tables S21 and S22). SSS was not significantly or meaningfully associated with ACE accuracy (β = -.01, *t* = -.01, *p* > .05, Table S21). but was a positive predictor of ACE bias (β = .164, *t* = 2.780, *p* < .01, Table S22). SSS was also negatively associated with hit rates in our US sample (β = -.150, *t* = -2.33, *p* < .05, Table S23). These latter results are in line with evidence from research that adopted traditional ERA measures, i.e., hit rates [7,13] and reinforce our argument for the need to examine ERA across different cultural contexts and with methods that take social context into consideration. As in Study 1 there were no significant gender by SSS interaction effects either on ACE accuracy or ACE bias (see Tables S21 and S22).

### Testing country-level social ecology moderators

To test the potential moderating effect of the targeted country-level variables (Gini, Long-term Orientation and relational mobility) a random intercept and random slope model outlined in (2) was fitted with Accuracy or Bias as respective outcome variables at the individual level predicted by relational mobility, Long-term Orientation and Gini index (both grand-mean cantered) at country-level. The results from those analyses are presented in Table 3. In exploratory analyses we firstly tested for possible effects of country-level individualism, power distance and masculinity using Hofstede [53] indicators, yet no significant or meaningful effects either on the ACE scores or the social status – ACE accuracy/bias relationships were observed.

**Table 3:** Multilevel model of relationships between SSS and ACE accuracy as a function of countries’ Long-Term Orientation (LTO) Relational Mobility (RM) and Gini.

						RM				LTO				Gini	
	Coef.	SE	t-value		Coef.	SE	t-value		Coef.	SE	t-value		Coef.	SE	t-value
Intercept _γ00_	3.118	.098	31.66***	_γ01_	-.045	.067	-.68	_γ02_	**-.004**	**.001**	-2.94*	_γ03_	**-.023**	**.007**	-3.13*
**SSS**_**γ10**_	**.017**	**.006**	2.63*	_γ11_	**-.023**	**.004**	-4.83**	_γ12_	**.0004**	**.0001**	2.79*	_γ13_	**-.002**	**.0007**	-3.40**
Gender. _γ20_	.145	.049	2.96*												
Age _γ30_	-.005	.001	-5.01**												
Bias _γ40_	.579	.037	15.26***												

*Note*: Coefficients in bold are described in the results section. Gender coded -1 = men, 1 = women * *p* < .05 ** *p* < .01, *** *p* < .001

(2) **Level 1:**
  y_ij_ = β_0j_ + β_1j_*SSS + β_2j_*Gender + β_3j_*Age +β_4j_*Bias + r_ij_.
**Level 2**
  β_0j_ = γ_*01 +*_ γ_*01*_ (Gini) + γ_*02*_ (LTO) + γ_*03*_ (RM) + μ_0j_
  β_1j_ = γ_*10 +*_ γ_*11*_ (Gini) + γ_*12*_ (LTO) + γ_*13*_ (RM) + μ_0j_
  β_2j_ = γ_*21*_ + μ_2j_
  β_3j_ = γ_*31 +*_ μ_3j_
  β_4j_ = γ_*41 +*_ μ_4j_

**Fig 2:**
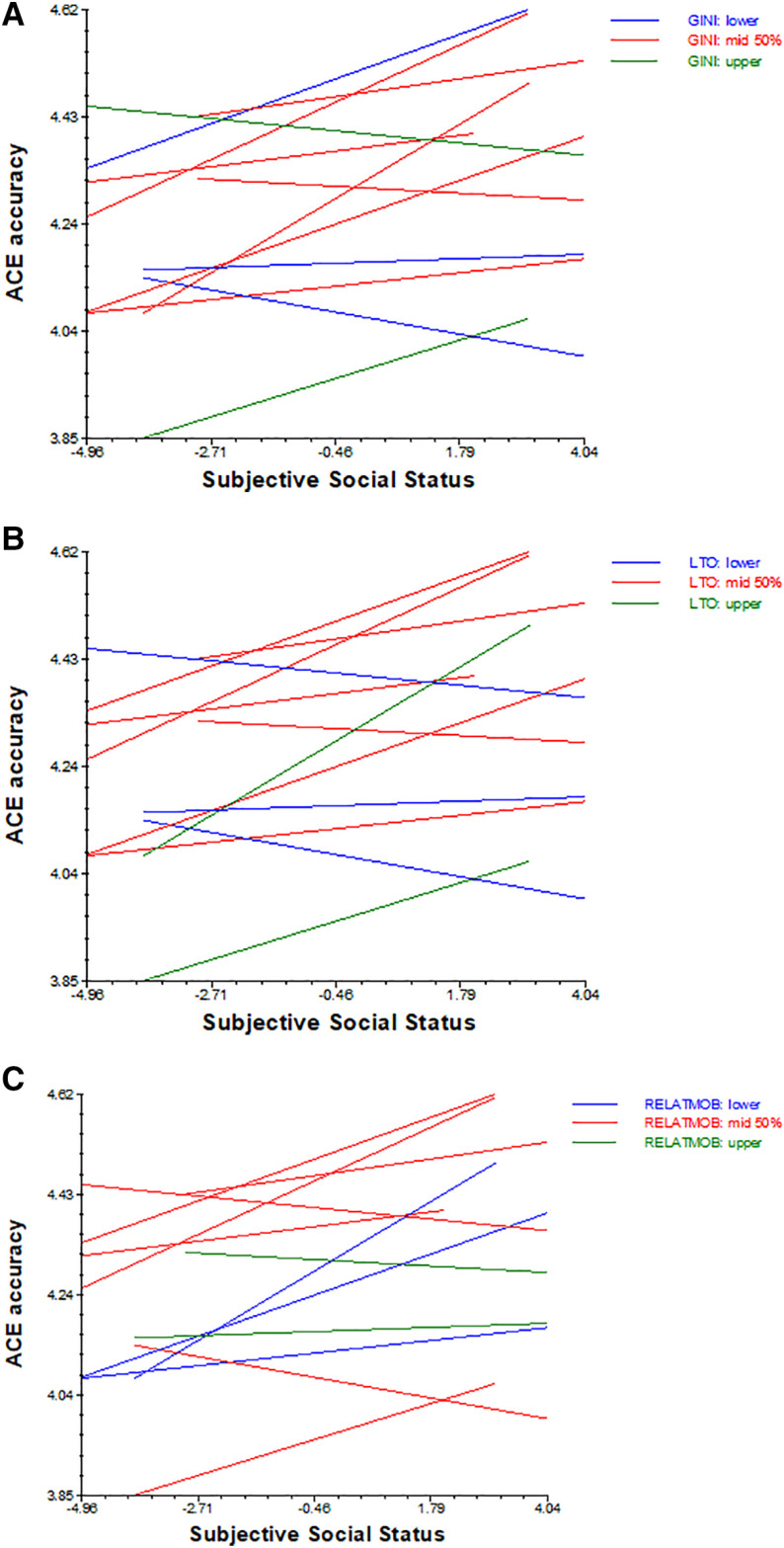
Relationships between social class and ACE accuracy as a function of country-level Long-Term Orientation (1a), Relational mobility (1b) and Gini (1c).

Higher Long-term Orientation and Gini were associated with lower scores on ACE accuracy overall (γ_02_ = -.004, *t* = -2.94, *p* < .05 and γ_01_ = -.023, *t* = -3.13, *p* < .05 respectively). Importantly, all three country level predictors significantly moderated relationships between social class and accuracy. The positive association between SSS and accuracy was strengthened in countries with higher Long-term Orientation (see Fig 2a) and was weakened in cultures with higher Relational Mobility and higher Gini (see Figs 2b and 1c). For example, as observed in Fig 2a in cultures with lower Long-term Orientation (namely, Ireland, USA, Poland) the association between SSS and ACE accuracy was even negative.

We then conducted the above-described analyses replacing SSS with parental education level. Neither Long-term Orientation, Gini or relational mobility moderated the negative relationship between parental educational level and ACE bias (γ_40_ = -.010, *t* = -2.62, *p* < .05); yet, relational mobility was a negative predictor of ACE bias overall (γ_03_ = -.125, *t* = -5.81, *p* < .001). A further test of participants’ own educational level on ACE bias (replacing parental educational level with participants’ respective education level measure) had no such effect on either the ACE bias or the ACE accuracy indices. Additional analyses to test SSS and parental educational level relationships with accuracy and bias separately for each of the four emotions expressed in the ACE (happy, anger, sad, disgust depicted in S3 to S6 Tables) as well as for the congruent and incongruent conditions found that SSS was consistently positively related with individual accuracy scores for each emotion and parental educational level with ACE bias scores for all emotions (Tables S12 to S18). Country level moderators, however, had more variable moderation effects on SSS and ERA relationships. Notably, SSS was more distinctly associated with ACE accuracy in the congruent emotion condition than the incongruent condition (see Tables S9 and S10). Notably, Gini, Long-term Orientation and relational mobility did not moderate SSS or parental education levels relationships with hit rate levels.

## Discussion

As part of a broader interest in the cognitive, behavioral, and emotional consequences of social class differences [6], higher social class has been repeatedly found to be associated with less accuracy in perceiving emotion [7]. However, this research was conducted primarily in North America and with tasks focusing on decontextualized facets of emotion perception (e.g., Diagnostic Analysis of Non-Verbal Accuracy, Reading the Mind in the Eyes Test). The present study addressed this context-free assessment and mono-cultural focus by collecting data from multiple cultures and using a contextualized emotion recognition accuracy assessment that has demonstrated internal and ecological validity across cultures and types of social interaction [27].

Using a community sample in the Czech Republic and student samples across 12 other world cultures we found that higher subjective social status (SSS) was associated with better emotion recognition accuracy using the Assessment of Contextualized Emotion (ACE), an Emotion Recognition Accuracy (ERA) model and method that incorporates social context and distinguishes between accuracy (the perception of intended emotions) and bias (the perception of additional emotions to those expressed), in line with the truth and bias model in social perception [30]. Notably, in both studies, subjective social status was not associated with either a hit rates assessment of ERA (as typically used in previous research, [7,13]) or with a standard assessment of non-contextualized emotions (Mayer Salovey Caruso Emotional Intelligence Test faces, Study 1). Interestingly as shown in Table S1 MSCEIT negatively correlated with ACE bias replicating previous results ([27,33]). As in previous research, bias perception negatively relates to MSCEIT Faces scores suggesting that both ACE bias and the MSCEIT scores reflect culturally normative perception. Moreover, contrary to results from a recent study in the US [72] gender was not found to moderate the subjective social status – accuracy relationship. This evidence is in support of our argument that the ACE provides a more nuanced approach to ERA compared to the categorical (hit rates) approach typically utilized in past research; it also replicates previous research that ACE accuracy and bias but not hit rates are related to constructs capturing social competence [33].

Importantly, the association between higher SSS and ACE accuracy was explained by the endorsement of independent self-construal at the person level. Given that higher SSS was positively associated with independent (but not with interdependent) self-construal, we assume that psychological tendencies associated with independence underlie the association between social status and ERA. Higher social class persons tend to adopt a more self-oriented mode of thinking [38]. As previous research using different versions of the ACE has shown [33,35], experimental manipulations or chronic measures of higher individualism led to better accuracy in recognizing facial emotional expressions in context. This effect can be explained by the observation that persons higher on independence employ a more deliberate, agentic and goal oriented cognitive style [23]. They therefore pay more attention to the emotional expressions of others and are able to decouple them from contextual “noise.” Thus, persons of higher social status, who are typically characterized by higher independence [38], also adopt this socio-cognitive strategy.

Taken together, we interpret this main part of the results as evidence of the influence of the more agentic and analytic modes of thinking that higher class persons adopt [9]. Higher power, which also partially overlaps with social status [73], drives higher action orientation for the perception of emotions [39,42] and this may also explain the positive associations between higher social class and emotion perception. Further evidence of the action-oriented tendencies associated with higher social status (and power) is that subjective social status was more strongly associated with ACE accuracy when the expressions shown by the target and the surrounding group were congruent rather than incongruent (group expressing neutral emotions). A group context (others expressing the same emotion as the central character) tends to amplify the perception of facial expressions at least in some cultures [27].

Additional evidence for a positive link between higher social class and ERA was provided by the observation, in the second study, that parental education level, an objective index of social status, was negatively associated with ACE bias. A negative association between parental education and ACE bias means that higher education level can predict greater ERA by reducing bias (the perception of emotions additional to the ones expressed). It warrants further investigation, however, into why parental educational level might lead to less bias but not to more accuracy in the ACE model whereas subjective social status is associated with more accuracy but not with less bias. Speculating on processes that link parental education to ACE bias, it is possible that exposure to higher education reduces interpersonal biases and stereotypes when judging other people [74]. ACE bias reflects in part the influence of stereotypes on emotion perception [21]. Specifically, ACE bias involves the perception/attribution of additional emotion categories, a process that is likely driven by beliefs and stereotypes. Overall, these results highlight the significance of differentiating between socio-cognitive processes that pertain to signal (accuracy) and those related to noise (bias) in emotion perception [30].

Moreover, the herein presented research provides novel insights into how social-ecological processes at the collective level (Long term orientation, Relational Mobility, Inequality) can explain different aspects of the relationship between social status and ERA. As expected, higher country-level Long-term orientation strengthened the positive SSS and ACE accuracy relationship. Higher social class persons are typically long-term oriented [46,47] and environs where people tend to exhibit more patience and delay of gratification (higher Long-term orientation, [49]) can augment instrumental and action-oriented tendencies associated with higher SSS. In fact, it is possible that this aspect of cultural differences coupled with differences in the rating tasks used can explain some of the contradictory findings between previous research mainly conducted in the US (a low Long-Term Orientation cultural context) and the present findings. For example, relationships between higher SSS and Hit rates accuracy were negative only for our US sample. Future research should test this directly by utilizing contextualized and more traditional stimuli together with contextualized emotion recognition methods and models. Such research would also need to distinguish facets of social status and power (and their overlap) that can account for more and less agentic modes of emotion recognition.

Higher culture-level inequality (higher Gini) was also found to weaken the SSS and emotion recognition accuracy association. The results regarding inequality were consistent with Schmalor and Heine’s [14] individual-level findings that higher inequality reduces ERA. Schmalor and Heine conjectured that the perception of inequality intensifies feelings of self-sufficiency which, in turn, exaggerate an already -- in their study negative -- relationship between SSS and ERA. In our study, however, higher inequality attenuated a *positive* relationship between SSS and accuracy. In that respect, one could consider the type of the task (contextualized facial emotion expressions) and its significance in view of peoples’ – unequal -- social ecologies. As well as signifying individual-level capabilities [14] the ACE entrains a social form of emotion perception [34], which can have a relational significance for peoples’ social communication [4]. Economic inequality can weaken trust and social capital and weaken social ties [75] thereby impacting such social forms of emotion perception.

Last, we explored the role of relational mobility, a behavioral indicator of social interaction, for the social status – ERA link. Whereas higher relational mobility was associated with lower ACE-bias overall, higher country-level relational mobility weakened the social status-accuracy association. The former finding is consistent with evidence that higher frequency of social interaction with others is beneficial for ERA [51,52] and future research should explicitly include a measure of frequency of social interaction and social networking. However, it is as of yet unclear why higher relational mobility weakened the positive relationship between SSS and ERA. It is possible that in more affiliative social ecologies (in high relationally mobile societies where people need to actively show affection to maintain relationships, [76]), everyone is expected to show more attention to others. This would then weaken the difference between higher and lower social class and thereby reduce the link between higher SSS and ERA.

Overall, the results are in line with Schmid Mast et al.’s [19] and Hall et al.’s [17] conclusion that the actual effect of relationships between verticality in social relations (social class, hierarchy, power) and accuracy in decoding others depends on the specific social structuring circumstances. Further research might aim to disentangle the individual- and collective-level processes of how Long-term-orientation and relational mobility influence the social and emotional perceptual abilities of individuals of higher and lower social status.

### Limitations

Despite its strengths (inclusion of a diverse set of world cultures, and a new method for assessing ERA), the present study has certain limitations that should be considered. First, Study 2 involved a younger generation, restricting the generalizability of our findings. Although focusing on younger persons guaranteed a level of comparability across cultures, at the same time younger samples who often include students (as is the case in Study 2), often are from families with higher SES and higher parental education and are thus not representative of the social status differences within each culture. Second, although the analyses included a good representation of countries around the world, and had adequate statistical power concerning questions at individual level [77], the moderate number of groups raises questions about ensuring adequate statistical generalizability at country-level. Therefore, the observed culture-individual-level interactions effects should be considered with caution. Future research should seek to increase the number of group-level observations and aim to disentangle status from power as predictors of ERA capabilities. For several of the tested relationships, the size of the effect was small to medium, a not uncommon phenomenon for moderation effects [78] and for research that combines cognitive with self-report methodologies [79]. Lastly, future research should explore whether the positive relationship between ACE and SSS arises from the possibility that ACE represents a more cognitively demanding emotion recognition task compared to those typically employed. This consideration aligns with evidence suggesting positive associations between higher social status and cognitive abilities, at least in certain cultural contexts [80].

## Conclusion

Using more culturally diverse samples than in previous research on the topic, the present study demonstrated that higher social status is typically associated with higher ERA in a contextualized assessment of ERA and this relationship is accounted for by participants’ higher independent self-construal. Moreover, country level cultural dimensions (Long Term Orientation, Relational Mobility, Inequality) were also found to moderate the relationship between ERA and social status variables. These results point to the function of social power in enhancing action orientation in emotion perception and the social ecologies of emotion perception across cultures.

## Supporting information

S1 Fig Residuals versus predicted values plots of ACE accuracy. (TIF)

S2 Fig Residuals versus predicted values plots of ACE bias. (TIF)

S4 Fig Relationships between Parental Education Level and ACE bias. (TIF)

S1 Table Descriptives and zero-order correlations among main Study 1 variable. (PDF)

S2 Table Regressing ACE accuracy on Subjective Social Status (SSS). (PDF)

S3 Table A review of studies on relationships between emotion recognition accuracy and social class. (PDF)

S4 Table Tucker’s Phi Coefficients for Self-Construal Scale Factors across Countries. (PDF)

S5 Table Multilevel model of relationships between Subjective Social Status (SSS) and ACE accuracy happy. (PDF)

S6 Table Multilevel model of relationships between Subjective Social Status (SSS) and ACE accuracy angry. (PDF)

S9 Table Multilevel model of relationships between Subjective Social Status (SSS) and ACE congruent accuracy. (PDF)

S10 Table Multilevel model of relationships between Subjective Social Status (SSS) and ACE incongruent accuracy. (PDF)

S12 Table Multilevel model of relationships between Parental Education Level (PEL) and ACE bias happy. (PDF)

S13 Table Multilevel model of relationships between Parental Education Level (PEL) and ACE bias angry. (PDF)

S14 Table Multilevel model of relationships between Parental Education Level (PEL) and ACE bias sad. (PDF)

S15 Table Multilevel model of relationships between Parental Education Level (PEL) and ACE bias disgust. (PDF)

S16 Table Multilevel model of relationships between Parental Education Level (PEL) and ACE bias congruent. (PDF)

S17 Table Multilevel model of relationships between Parental Education Level (PEL) and ACE bias incongruent. (PDF)

S18 Table Multilevel model of relationships between Parental Education Level (PEL) and ACE bias. (PDF)

S21 Table Predicting ACE Accuracy Rates across each of the 12 cultures. (PDF)

S22 Table Predicting ACE bias rates across each of the 12 cultures. (PDF)

S1 File This is the Supplemental File. (PDF)

## References

[1] Fischer AH, Manstead ASR. Social functions of emotion. Handbook of emotions. 3rd ed. New York, NY, US: The Guilford Press; 2008. pp. 456–468.

[2] Keltner D, Haidt J. Social functions of emotions. Emotions: Currrent issues and future directions. New York, NY, US: The Guilford Press; 2001. pp. 192–213.

[3] Feldman RS, Philippot P, Custrini RJ. Social competence and nonverbal behavior. Fundamentals of nonverbal behavior. New York, NY, US: Cambridge University Press; 1991. pp. 329–350.

[4] Niedenthal PM, Brauer M. Social functionality of human emotion. Annu Rev Psychol. 2012;63:259–85. doi: 10.1146/annurev.psych.121208.131605 22017377

[5] Kraus MW, Keltner D. Signs of socioeconomic status: a thin-slicing approach. Psychol Sci. 2009;20(1):99–106. doi: 10.1111/j.1467-9280.2008.02251.x 19076316

[6] Manstead ASR. The psychology of social class: How socioeconomic status impacts thought, feelings, and behaviour. Br J Soc Psychol. 2018;57(2):267–91. doi: 10.1111/bjso.12251 29492984 PMC5901394

[7] Kraus MW, Côté S, Keltner D. Social class, contextualism, and empathic accuracy. Psychol Sci. 2010;21(11):1716–23. doi: 10.1177/0956797610387613 20974714

[8] Kraus MW, Piff PK, Keltner D. Social class as culture. Curr Dir Psychol Sci. 2011;20(4):246–50. doi: 10.1177/0963721411414654

[9] Kraus MW, Piff PK, Mendoza-Denton R, Rheinschmidt ML, Keltner D. Social class, solipsism, and contextualism: how the rich are different from the poor. Psychol Rev. 2012;119(3):546–72. doi: 10.1037/a0028756 22775498

[10] Mayer JD, Salovey P, Caruso DR, Sitarenios G. Measuring emotional intelligence with the MSCEIT V2.0. Emotion. 2003;3(1):97–105. doi: 10.1037/1528-3542.3.1.97 12899321

[11] Adler NE, Epel ES, Castellazzo G, Ickovics JR. Relationship of subjective and objective social status with psychological and physiological functioning: preliminary data in healthy white women. Health Psychol. 2000;19(6):586–92. doi: 10.1037//0278-6133.19.6.586 11129362

[12] Baron‐Cohen S, Wheelwright S, Hill J, Raste Y, Plumb I. The “Reading the Mind in the Eyes” test revised version: a study with normal adults, and adults with asperger syndrome or high‐functioning autism. Child Psychol Psychiatry. 2001;42(2):241–51. doi: 10.1111/1469-7610.00715 11280420

[13] Dietze P, Knowles ED. Social class predicts emotion perception and perspective-taking performance in adults. Pers Soc Psychol Bull. 2021;47(1):42–56. doi: 10.1177/0146167220914116 32336209

[14] Schmalor A, Heine SJ. Subjective economic inequality decreases emotional intelligence, especially for people of high social class. Soc Psychol Personal Sci. 2022;13(2):608–17. doi: 10.1177/19485506211024024 35251492 PMC8892066

[15] Monroy M, Cowen AS, Keltner D. Intersectionality in emotion signaling and recognition: the influence of gender, ethnicity, and social class. Emotion. 2022;22(8):1980–8. doi: 10.1037/emo0001082 35389737

[16] Deveney CM, Chen SH, Wilmer JB, Zhao V, Schmidt HB, Germine L. How generalizable is the inverse relationship between social class and emotion perception? PLoS One. 2018;13(10):e0205949. doi: 10.1371/journal.pone.0205949 30339671 PMC6195285

[17] Hall JA, Schmid Mast M, Latu I-M. The vertical dimension of social relations and accurate interpersonal perception: a meta-analysis. J Nonverbal Behav. 2014;39(2):131–63. doi: 10.1007/s10919-014-0205-1

[18] Magee JC, Galinsky AD. 8 social hierarchy: the self‐reinforcing nature of power and status. ANNALS. 2008;2(1):351–98. doi: 10.5465/19416520802211628

[19] Schmid Mast M, Jonas K, Hall JA. Give a person power and he or she will show interpersonal sensitivity: the phenomenon and its why and when. J Pers Soc Psychol. 2009;97(5):835–50. doi: 10.1037/a0016234 19857005

[20] Schmid Mast M, Khademi M, Palese T. Power and social information processing. Curr Opin Psychol. 2020;33:42–6. doi: 10.1016/j.copsyc.2019.06.017 31374370

[21] Hess U, Kafetsios K. Infusing context into emotion perception impacts emotion decoding accuracy. Exp Psychol. 2021;68(6):285–94. doi: 10.1027/1618-3169/a000531 35258359 PMC9446470

[22] Stephens NM, Markus HR, Townsend SSM. Choice as an act of meaning: the case of social class. J Pers Soc Psychol. 2007;93(5):814–30. doi: 10.1037/0022-3514.93.5.814 17983302

[23] Markus HR, Kitayama S. Cultures and selves: a cycle of mutual constitution. Perspect Psychol Sci. 2010;5(4):420–30. doi: 10.1177/1745691610375557 26162188

[24] Barrett LF, Kensinger EA. Context is routinely encoded during emotion perception. Psychol Sci. 2010;21(4):595–9. doi: 10.1177/0956797610363547 20424107 PMC2878776

[25] Hess U, Hareli S. The impact of context on the perception of emotions. 1st ed. In: Abell C, Smith J, editors. The expression of emotion. 1st ed. Cambridge University Press; 2016. pp. 199–218. doi: 10.1017/cbo9781316275672.010

[26] Kafetsios K, Hess U. Reconceptualizing emotion recognition ability. J Intell. 2023;11(6):123. doi: 10.3390/jintelligence11060123 37367525 PMC10301294

[27] Hess U, Kafetsios K, Mauersberger H, Blaison C, Kessler C-L. Signal and noise in the perception of facial emotion expressions: from labs to life. Pers Soc Psychol Bull. 2016;42(8):1092–110. doi: 10.1177/0146167216651851 27277281

[28] Kafetsios K, Hess U. Seeing mixed emotions: Alexithymia, emotion perception bias, and quality in dyadic interactions. Pers Individ Differ. 2019;137:80–5. doi: 10.1016/j.paid.2018.08.014

[29] Kafetsios K, Hess U, Dostal D, Seitl M, Hypsova P, Hareli S, et al. A contextualized emotion perception assessment relates to personal and social well-being. J Res Pers. 2025;114:104556. doi: 10.1016/j.jrp.2024.104556

[30] West TV, Kenny DA. The truth and bias model of judgment. Psychol Rev. 2011;118(2):357–78. doi: 10.1037/a0022936 21480740

[31] Funder DC. On the accuracy of personality judgment: a realistic approach. Psychol Rev. 1995;102(4):652–70. doi: 10.1037/0033-295x.102.4.652 7480467

[32] Zaki J. Cue integration: a common framework for social cognition and physical perception. Perspect Psychol Sci. 2013;8(3):296–312. doi: 10.1177/1745691613475454 26172972

[33] Kafetsios K, Hess U. Personality and the accurate perception of facial emotion expressions: what is accuracy and how does it matter? Emotion. 2022;22(1):100–14. doi: 10.1037/emo0001034 35099242

[34] Antypa D, Kafetsios K, Simos P, Kyvelea M, Kosteletou E, Maris T, et al. Distinct neural correlates of accuracy and bias in the perception of facial emotion expressions. Soc Neurosci. 2024;19(3):215–28. doi: 10.1080/17470919.2024.2403187 39297912

[35] Kafetsios K, Hess U. Effects of Activated and dispositional self-construal on emotion decoding accuracy. J Nonverbal Behav. 2013;37(3):191–205. doi: 10.1007/s10919-013-0149-x

[36] Kitayama S, Duffy S, Kawamura T, Larsen JT. Perceiving an object and its context in different cultures: a cultural look at new look. Psychol Sci. 2003;14(3):201–6. doi: 10.1111/1467-9280.02432 12741741

[37] Masuda T, Ellsworth PC, Mesquita B, Leu J, Tanida S, Van de Veerdonk E. Placing the face in context: cultural differences in the perception of facial emotion. J Pers Soc Psychol. 2008;94(3):365–81. doi: 10.1037/0022-3514.94.3.365 18284287

[38] Gobel MS, Miyamoto Y. Self- and other-orientation in high rank: a cultural psychological approach to social hierarchy. Pers Soc Psychol Rev. 2024;28(1):54–80. doi: 10.1177/10888683231172252 37226514 PMC10851657

[39] Galinsky AD, Gruenfeld DH, Magee JC. From power to action. J Pers Soc Psychol. 2003;85(3):453–66. doi: 10.1037/0022-3514.85.3.453 14498782

[40] Na J, Chan MY. Subjective perception of lower social-class enhances response inhibition. Pers Individ Differ. 2016;90:242–6. doi: 10.1016/j.paid.2015.11.027

[41] Sheehy-Skeffington J. The effects of low socioeconomic status on decision-making processes. Curr Opin Psychol. 2020;33:183–8. doi: 10.1016/j.copsyc.2019.07.043 31494518

[42] Guinote A. Power and goal pursuit. Pers Soc Psychol Bull. 2007;33(8):1076–87. doi: 10.1177/0146167207301011 17575244

[43] Piff PK, Kraus MW, Côté S, Cheng BH, Keltner D. Having less, giving more: the influence of social class on prosocial behavior. J Pers Soc Psychol. 2010;99(5):771–84. doi: 10.1037/a0020092 20649364

[44] Miyamoto Y, Yoo J, Levine CS, Park J, Boylan JM, Sims T, et al. Culture and social hierarchy: Self- and other-oriented correlates of socioeconomic status across cultures. J Pers Soc Psychol. 2018;115(3):427–45. doi: 10.1037/pspi0000133 29771553 PMC6095715

[45] LESHAN LL. Time orientation and social class. J Abnorm Psychol. 1952;47(3):589–92. doi: 10.1037/h0056306 12980757

[46] Trommsdorff G. Future orientation and socialization*. Int J Psychol. 1983;18(1–4):381–406. doi: 10.1080/00207598308247489

[47] Watts TW, Duncan GJ, Quan H. Revisiting the marshmallow test: a conceptual replication investigating links between early delay of gratification and later outcomes. Psychol Sci. 2018;29(7):1159–77. doi: 10.1177/0956797618761661 29799765 PMC6050075

[48] Schwartz A, Eyal T, Tamir M. Emotions and the big picture: the effects of construal level on emotional preferences. J Exp Soc Psychol. 2018;78:55–65. doi: 10.1016/j.jesp.2018.05.005

[49] Hofstede G, Minkov M. Long- versus short-term orientation: new perspectives. Asia Pacific Business Rev. 2010;16(4):493–504. doi: 10.1080/13602381003637609

[50] Thomson R, Yuki M, Talhelm T, Schug J, Kito M, Ayanian AH, et al. Relational mobility predicts social behaviors in 39 countries and is tied to historical farming and threat. Proc Natl Acad Sci U S A. 2018;115(29):7521–6. doi: 10.1073/pnas.1713191115 29959208 PMC6055178

[51] Balas B, Saville A. Hometown size affects the processing of naturalistic face variability. Vision Res. 2017;141:228–36. doi: 10.1016/j.visres.2016.12.005 28025050 PMC5494272

[52] Calvo MG, Gutiérrez-García A, Fernández-Martín A, Nummenmaa L. Recognition of facial expressions of emotion is related to their frequency in everyday life. J Nonverbal Behav. 2014;38(4):549–67. doi: 10.1007/s10919-014-0191-3

[53] Hofstede G. Culture’s consequences: comparing values, behaviors, institutions, and organizations across nations. 2nd ed. Thousand Oaks, CA: Sage; 2001. Available: https://digitalcommons.usu.edu/unf_research/53

[54] Vignoles VL, Kirchner-Häusler A, Uskul AK, Cross SE, Rodriguez-Bailón R, Bossom IRL, et al. Are mediterranean societies “Cultures of Honor?”: prevalence and implications of a cultural logic of honor across three world regions. Pers Soc Psychol Bull. 2024. doi: 10.1177/01461672241295500 PMC13022031 41883064

[55] Snibbe AC, Markus HR. You can’t always get what you want: educational attainment, agency, and choice. J Pers Soc Psychol. 2005;88(4):703–20. doi: 10.1037/0022-3514.88.4.703 15796669

[56] Faul F, Erdfelder E, Buchner A, Lang A-G. Statistical power analyses using G*Power 3.1: tests for correlation and regression analyses. Behav Res Methods. 2009;41(4):1149–60. doi: 10.3758/BRM.41.4.1149 19897823

[57] Harkness JA, Edwards B, Hansen SE, Miller DR, Villar A. Designing questionnaires for multipopulation research. 1st ed. In: Harkness JA, Braun M, Edwards B, Johnson TP, Lyberg L, Mohler PPh, et al., editors. Survey methods in multinational, multiregional, and multicultural contexts. 1st ed. Wiley; 2010. pp. 31–57. doi:10.1002/9780470609927.ch3

[58] Hess U, Blairy S, Kleck RE. J Nonverbal Behavior. 1997;21(4):241–57. doi: 10.1023/a:1024952730333

[59] Singelis TM, Triandis HC, Bhawuk DPS, Gelfand MJ. Horizontal and vertical dimensions of individualism and collectivism: a theoretical and measurement refinement. Cross-Cultural Res. 1995;29(3):240–75. doi: 10.1177/106939719502900302

[60] Lacko D, Čeněk J, Urbánek T. Psychometric properties of the independent and interdependent self-construal questionnaire: evidence from the Czech Republic. Front Psychol. 2021;12:564011. doi: 10.3389/fpsyg.2021.564011 34149489 PMC8209258

[61] Rudnev M, Barrett HC, Buckwalter W, Machery E, Stich S, Barr K, et al. Dimensions of wisdom perception across twelve countries on five continents. Nat Commun. 2024;15(1):6375. doi: 10.1038/s41467-024-50294-0 39143069 PMC11324649

[62] Green P, MacLeod CJ. SIMR: an R package for power analysis of generalized linear mixed models by simulation. Methods Ecol Evol. 2016;7(4):493–8. doi: 10.1111/2041-210x.12504

[63] Kwan VS, Bond MH, Singelis TM. Pancultural explanations for life satisfaction: adding relationship harmony to self-esteem. J Pers Soc Psychol. 1997;73(5):1038–51. doi: 10.1037//0022-3514.73.5.1038 9364759

[64] Raudenbush SW, Bryk AS, Cheong YF, Congdon R. HLM 7.03 for Windows. 2013.

[65] Nezlek JB. Multilevel modeling and cross-cultural research. 1st ed. In: Matsumoto D, Van De Vijver FJR, editors. Cross-cultural research methods in psychology. 1st ed. Cambridge University Press; 2010. pp. 299–345. doi:10.1017/CBO9780511779381.015

[66] Mathieu JE, Aguinis H, Culpepper SA, Chen G. Understanding and estimating the power to detect cross-level interaction effects in multilevel modeling. J Appl Psychol. 2012;97(5):951–66. doi: 10.1037/a0028380 22582726

[67] Snijders T, Bosker R. Multilevel analysis: an introduction to basic and advanced multilevel modeling. Thousand Oaks, Calif.: Sage Publishing; 2012.

[68] Quesque F, Coutrot A, Cox S, de Souza LC, Baez S, Cardona JF, et al. Does culture shape our understanding of others’ thoughts and emotions? An investigation across 12 countries. Neuropsychology. 2022;36(7):664–82. doi: 10.1037/neu0000817 35834208 PMC11186050

[69] Malatesta CZ, Izard CE, Culver C, Nicolich M. Emotion communication skills in young, middle-aged, and older women. Psychol Aging. 1987;2(2):193–203. doi: 10.1037//0882-7974.2.2.193 3268208

[70] Rosip JC, Hall JA. Knowledge of nonverbal cues, gender, and nonverbal decoding accuracy. J Nonverbal Behav. 2004;28(4):267–86. doi: 10.1007/s10919-004-4159-6

[71] Peugh JL. A practical guide to multilevel modeling. J Sch Psychol. 2010;48(1):85–112. doi: 10.1016/j.jsp.2009.09.002 20006989

[72] Brener SA, Frankenhuis WE, Young ES, Ellis BJ. Social class, sex, and the ability to recognize emotions: the main effect is in the interaction. Pers Soc Psychol Bull. 2024;50(8):1197–210. doi: 10.1177/01461672231159775 37013847

[73] Anderson C, Srivastava S, Beer JS, Spataro SE, Chatman JA. Knowing your place: self-perceptions of status in face-to-face groups. J Pers Soc Psychol. 2006;91(6):1094–110. doi: 10.1037/0022-3514.91.6.1094 17144767

[74] Jussim L, Crawford JT, Rubinstein RS. Stereotype (In)accuracy in perceptions of groups and individuals. Curr Dir Psychol Sci. 2015;24(6):490–7. doi: 10.1177/0963721415605257

[75] Wilkinson RD, Pickett K. The spirit level: why more equal societies almost always do better. New York, NY, US: Bloomsbury Publishing; 2009. pp. xvii, 331.

[76] Kito M, Yuki M, Thomson R. Relational mobility and close relationships: a socioecological approach to explain cross‐cultural differences. Pers Relationsh. 2017;24(1):114–30. doi: 10.1111/pere.12174

[77] Snijders T. Power and sample size in multilevel modeling. 2005;3:1570–3.

[78] Aguinis H, Beaty JC, Boik RJ, Pierce CA. Effect size and power in assessing moderating effects of categorical variables using multiple regression: a 30-year review. J Appl Psychol. 2005;90(1):94–107. doi: 10.1037/0021-9010.90.1.94 15641892

[79] Podsakoff PM, MacKenzie SB, Lee J-Y, Podsakoff NP. Common method biases in behavioral research: a critical review of the literature and recommended remedies. J Appl Psychol. 2003;88(5):879–903. doi: 10.1037/0021-9010.88.5.879 14516251

[80] Howard SJ, Cook CJ, Everts L, Melhuish E, Scerif G, Norris S, et al. Challenging socioeconomic status: a cross-cultural comparison of early executive function. Dev Sci. 2020;23(1):e12854. doi: 10.1111/desc.12854 31077525

