# Supplementary figures and images for: Higher social class is associated with higher contextualized emotion recognition accuracy across cultures
Source: PLoS One. 2025 May 13;20(5):e0323552. doi: 10.1371/journal.pone.0323552 (PMC12074547; doi:10.1371/journal.pone.0323552)

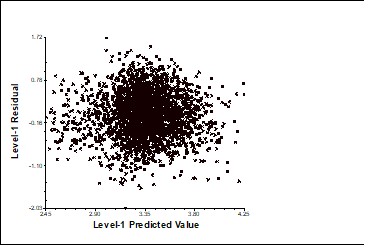

Supplement: S1 Fig — (TIF) [file pone.0323552.s001.tif]

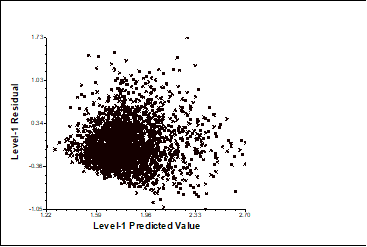

Supplement: S2 Fig — (TIF) [file pone.0323552.s002.tif]

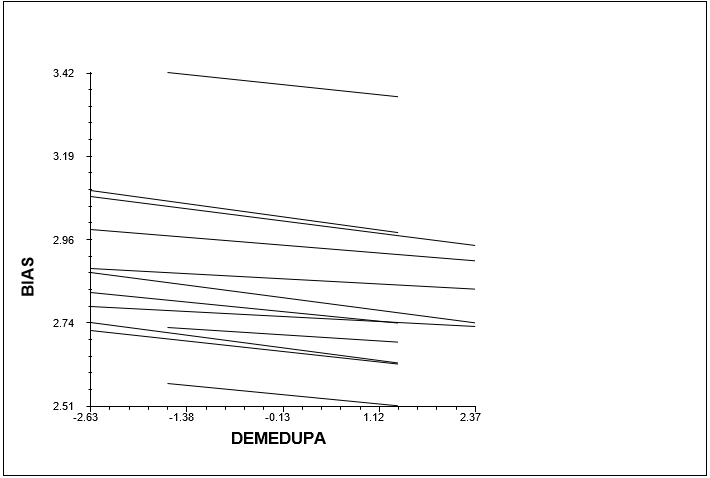

Supplement: S4 Fig — (TIF) [file pone.0323552.s003.tif]
