# Supplementary material for: Higher social class is associated with higher contextualized emotion recognition accuracy across cultures
Source: PLoS One. 2025 May 13;20(5):e0323552. doi: 10.1371/journal.pone.0323552 (PMC12074547; doi:10.1371/journal.pone.0323552)
Supplement: S1 Table — (PDF) [file pone.0323552.s004.pdf]

Table S1

Descriptives and zero-order correlations among main Study 1 variables

	1	2	3	4	5	6	7	8	9	10	11	12
1 Gender	-	.057	.058	-.024	-.074	.121*	-.018	-.143**	.201**	-.092	.041	-.127*
2 Parental education level		-	.266**	-.330**	.033	.072	-.006	.010	.111*	-.006	.061	.080
3 SSS			-	-.410**	.160**	.147**	-.067	-.032	.180**	.053	.098	.009
4 SES				-	-.083	-.087	.029	-.009	-.184**	.017	-.049	-.116*
5 ACE accuracy					.79	.476**	-.017	-.121*	.075	.088	-.058	.138*
6 ACE bias						.96	-.527**	-.517**	.088	.037	-.086	.173**
7 Hit rates							.72	.284**	-.015	-.063	-.006	-.145**
8 MSCEIT faces								.75	-.071	.001	-.007	-.089
9 Vert. individualism									.81	-.091	.203**	.153**
10 Horiz. collectivism										.72	-.183**	.352**
11 Horiz. individualism											.59	-.186**
12 Vert. collectivism												.63
Mean	1.287	3.789	6.078	2.905	5.192	2.329	0.428	44.547	4.772	6.503	6.451	4.461
SD	0.453	1.331	1.330	0.757	0.755	0.575	0.161	7.762	1.399	1.061	0.982	1.177

Note: SSS = Subjective social status, SES = Socio-Economic Status
