# Supplementary material for: Higher social class is associated with higher contextualized emotion recognition accuracy across cultures
Source: PLoS One. 2025 May 13;20(5):e0323552. doi: 10.1371/journal.pone.0323552 (PMC12074547; doi:10.1371/journal.pone.0323552)
Supplement: S2 Table — (PDF) [file pone.0323552.s005.pdf]

Table S2a

Regressing ACE accuracy on Subjective Social Status (SSS)

	β	t	p value
Constant	3.691	16.710	.000
SSS	.102	2.134	.034
Gender	-.144	-3.012	.003
ACE bias	.477	9.932	.000
$F_{(4,334)}$	37.870		
R^2	.256		

Table S2b

Regressing ACE accuracy Single on Subjective Social Status (SSS)

	β	t	p value
Constant	3.739	13.043	.000
SSS	.086	1.769	.078
Gender	-.20	-4.199	.000
Age	.063	1.323	.187
Bias Single	.448	9.237	.000
$F_{(4,334)}$	26.514		
R^2	.240		
