# Supplementary material for: Higher social class is associated with higher contextualized emotion recognition accuracy across cultures
Source: PLoS One. 2025 May 13;20(5):e0323552. doi: 10.1371/journal.pone.0323552 (PMC12074547; doi:10.1371/journal.pone.0323552)
Supplement: S3 Table — (PDF) [file pone.0323552.s006.pdf]

Table S3
A review of studies on relationships between emotion recognition accuracy and social class

Citation	Sample	Emotion recognition accuracy measure	Social class/status measure	Relevant findings
**Studies that support a positive relationship between Social class/ status and EDA**				
Schmid Mast, M., Jonas, K., & Hall, J. A. (2009). Give a Person Power and He or She Will Show Interpersonal Sensitivity: The Phenomenon and Its Why And When. Journal of Personality and Social Psychology, 97(5), 835–850. https://doi.org/10.1037/a0016234	Study 1 (N = 76);<br>Study 2 (N = 134);<br>Study 3 (N = 96);<br>Study 4 (N = 157)	*Study 1*<br>Participants watched videotaped superior-subordinate interactions and inferred the thoughts and feelings of subordinates – accuracy was rated based on the similarity between their inferences and the subordinates' self-reported thoughts or feelings<br><br>*Study 2*<br>Similar to Study 1 (however, participants also assessed superiors' thoughts and feelings)<br><br>*Study 3*<br>The Diagnostic Analysis of Nonverbal Accuracy 2 (DANVA2) (Nowicki & Duke, 1994)<br><br>*Study 4*<br>Profile of Nonverbal Sensitivity (PONS; Rosenthal et al., 1979)	*Study 1*<br>Assigned high (leader)- or low-power role (leader's assistant)<br><br>*Study 2*<br>Word completion task was used to prime participants with either high or low power or with neutral words<br>*Study 3*<br>Participants were randomly assigned to high-power, low-power, or control conditions<br>*Study 4*<br>Participants were randomly assigned to one of three conditions – egoistic, empathic, or neutral (control) – and asked to take the leader's perspective based on a descriptive paragraph.	*Study 1*<br>There was a significant role main effect in the predicted direction, F(1, 72) = 4.91, p = .03, effect size r = .25, with leaders being more interpersonally sensitive (M = 0.52) than assistants (M = 0.43)<br><br>*Study 2*<br>Significant priming main effect, $F(2, 128) = 2.51$, $p = .042$ (one-tailed), effect size r = .14, showing that participants who were primed with high power were more interpersonally sensitive (M = 0.90) than participants who were primed with low power (M = 0.78) and that neutral participants lay in between (M = 0.84)<br><br>*Study 3*<br>The linear contrast was F(1, 93) = 6.04, p = .05, effect size r = .25. The contrast between high power and control was also significant, F(1, 93) =

				5.20, p = .05, effect size r = .23. The low-power and control conditions did not differ (F < 1)<br><br>*Study 4*<br>Results showed a main effect of condition, F(2, 153) = 2.49, p = .04 (one-tailed), indicating that participants identifying with an empathic leader were more interpersonally sensitive (M = 0.71) than participants identifying with an egoistic leader (M = 0.68); participants identifying with the leader in the control condition were in between (M = 0.70)
Schmid Mast, M., & Darioly, A. (2014). Emotion recognition accuracy in hierarchical relationships. Swiss Journal of Psychology, 73(2), 69–75. https://doi.org/10.1024/1421-0185/a000124	A group of 142 superior-subordinate dyads of employees from different companies and organizations (total N = 284)	Diagnostic Analysis of Nonverbal Accuracy – Adult Faces (DANVA-AF-2; Nowicki & Duke, 1994)	Actual hierarchical relationship	Status was a significant positive predictor of ERA, showing that superiors were significantly better in ERA than their subordinates
Hall, J. A., Schmid Mast, M., & Latu, I.-M. (2015). The vertical dimension of social relations and accurate interpersonal perception: A meta-analysis. *Journal of Nonverbal Behavior*, *39*(2), 131–163. https://doi.org/10.1007/s10919-014-0205-1	67 independent studies totaling 15,505 participants	Diverse measures of general accuracy defined as perceiving other people's cues	SES (e.g., own or parents' education, score on a social class index)	Higher social status had a non-significant association with ERA but predicted higher interpersonal accuracy overall

Momm, T., Blickle, G., Liu, Y., Wihler, A., Kholin, M., & Menges, J. I. (2015). It pays to have an eye for emotions: Emotion recognition ability indirectly predicts annual income. *Journal of Organizational Behavior*, 36(1), 147–163. https://doi.org/10.1002/job.1975	N = 142 employee–peer–supervisor triads	The Diagnostic Analysis of Nonverbal Accuracy 2 (DANVA2; Nowicki & Carton, 1993; Baum & Nowicki, 1998)	Annual income	ERA was linked to annual income as an objective indicator of career success through the sequential mediation of peer-rated political skill and supervisor-rated interpersonal facilitation. The path coefficient of interpersonal facilitation on income was β = .16 ($p < .05$; $R^2 = .47$ with all control variables), and the indirect effect was significant (estimate = 0.036; SE = 0.019; 90 percent BC-CI [0.004; 0.068])
Bjornsdottir, R. T., Alaei, R., & Rule, N. O. (2017). The perceptive proletarian: Subjective social class predicts interpersonal accuracy. *Journal of Nonverbal Behavior*, *41*(2), 185-201. https://doi.org/10.1007/s10919-016-0248-6	Study 3 (N = 200)	Study 3 Baron-Cohen et al.'s (2001) RMET & Categorization of greyscale university yearbook portraits of the faces of 60 American male and female undergraduates (primarily Caucasian) who self-identified as Democrats or Republicans used in previous work (Rule and Ambady 2010).	Income as Objective SES indicator<br>MacArthur Scale of Subjective Social Status (Subjective SES) to measure their subjective SES (Adler et al. 2000).	Study 3 Controlling for Subjective SES, and education, rncome was **positively** associated with RMET scores (B = .02, SE = .01, $t(193) = 2.12$, $p = .04$)
**Studies that support a Negative relationship between Social status & EDA**				
Kraus, M. W., Côté, S., & Keltner, D. (2010). Social class, contextualism, and empathic accuracy. *Psychological Science*, *21*(11), 1716–1723. https://doi.org/10.1177/0956797610387613	Study 1 (N = 200);<br>Study 2 (N = 106);<br>Study 3 (N = 81)	*Study 1*<br>MSCEIT (the 20-item subscale score for the ability to identify emotions in photographs of human faces)<br>*Study 2*<br>Participants rated their own emotions	*Study 1*<br>Educational attainment of participants<br>*Study 2*<br>Participants rated themselves on a ladder that had 10 rungs representing	*Study 1*<br>High-school-educated participants scored higher in empathic accuracy than their college-educated counterparts, $F(1, 196) = 5.18$, $p < .05$.<br><br>*Study 2*<br>Lower class, as

		and estimated their partner's emotions during a hypothetical job interview<br>*Study 3*<br>Mind in the Eyes task (Baron-Cohen et al, 2001)	where people stood in the university community<br>*Study 3*<br>Manipulation of social class was adapted from measures of subjective perceptions of socioeconomic rank (Adler et al., 2000; Kraus et al., 2009)	measured by subjective SES, was associated with greater empathic accuracy, r(104) = –.20, p < .05<br><br>*Study 3*<br>Participants experimentally induced to experience lower-class rank were better able than their upper-class-rank counterparts to discern emotions from subtle expressions in the eyes, F(1, 74) = 4.48, p < .05.
Bjornsdottir, R. T., Alaei, R., & Rule, N. O. (2017). The perceptive proletarian: Subjective social class predicts interpersonal accuracy. *Journal of Nonverbal Behavior*, *41*(2), 185-201. https://doi.org/10.1007/s10919-016-0248-6	Study 2 (N = 150)<br>Study 3 (N = 200)<br>Study 4 (N = 220)	Study 2 Baron-Cohen et al.'s (2001) RMET; Categorization task: participants view randomly-ordered greyscale images of the eyes of 36 Caucasian men and women and choose the word that best describes what the target is thinking or feeling from a set of four options based on their first impressions.<br>Study 3 Baron-Cohen et al.'s (2001) RMET & Categorization of greyscale university yearbook portraits of the faces of 60 American male and female undergraduates (primarily Caucasian) who self-identified as Democrats or Republicans used in previous work (Rule and Ambady 2010).	Income as Objective SES indicator<br>MacArthur Scale of Subjective Social Status (Subjective SES) to measure their subjective SES (Adler et al. 2000).	Study 2 Controlling for objective SES, and education, Subjective SES was **negatively** associated with RMET scores B = -.05, SE = .02, t(146) = -2.89, p = .004<br><br>Study 3 Controlling for objective SES, and education, Subjective SES was **negatively** associated with RMET scores (B = -.03, SE = .02, t(193) = -2.03, p = .04). Income was **positively** associated with RMET scores (B = .02, SE = .01, t(193) = 2.12, p = .04)<br><br>Study 4 subjective SES significantly negatively predicted categorization accuracy,

		Study 4 Baron-Cohen et al.'s (2001) RMET, Mini PONS (Rosenthal et al. 1979)		B = -.02, SE = .01, t(216) = -2.30, p = .02,<br>Mini PONS scores, B = -.03, SE = .01, t(197) = -2.64, p = .01, and RME performance, B = -.05, SE = .02, t(216) = -3.12, p = .002
Deveney, C. M., Chen, S. H., Wilmer, J. B., Zhao, V., Schmidt, H. B., & Germine, L. (2018). How generalizable is the inverse relationship between social class and emotion perception?. *PloS one, 13*(10), e0205949. https://doi.org/10.1371/journal.pone.0205949	Study 1 (N = 179);<br>Study 2 (N = 5,187);<br>Study 3 (N = 2,564)<br>Study 4<br>3,859	*Study 1*<br>RMET (Baron-Cohen et al., 2001);<br>Vocabulary test (Richler et al., 2017)<br><br>*Study 2*<br>RMET (Baron-Cohen et al., 2001);<br>Vocabulary test (Richler et al., 2017)<br><br>*Study 3*<br>A multiracial emotion matching test<br><br>*Study 4*<br>The Queen Square Face Discrimination Test: Emotion and Identity Subtests (Garrido et al., 2009)	*Study 1*<br>Subjective SC:<br>status manipulation condition;<br>participant ladder ranking<br>Objective SC:<br>highest level of education;<br>annual income<br><br>*Study 2*<br>family income and parental education questions;<br>participants highest level of education<br><br>*Study 3*<br>participants highest level of education<br><br>*Study 4*<br>family income and parental education questions;<br>participants highest level of education	*Study 1*<br>Emotion identification correlated negatively with subjective social class (β = -0.15, 95% CI = [-0.28,-0.02]) and one of two objective social class measures (participant education β = -0.15, 95% CI = [-0.03,-0.01])<br><br>*Study 2*<br>Complex emotion identification correlated non-significantly with participant education (β = 0.02, p = 0.25; 95% CI = [-0.01, 0.05], n = 2,726), positively with childhood family income (β = 0.03, 95% CI = [0.01,0.06], n = 4,312), and positively with parental education (β = 0.06, 95% CI = [0.04,0.09], n = 4,225)<br>*Study 3*<br>Basic emotion identification correlated positively with participant education (β = 0.05, 95% CI = [0.02, 0.09]), n = 2,564)<br>*Study 4*

				Basic emotion discrimination correlated positively with participant education (β = 0.09, 95% CI = [0.05,0.13], n = 2,079), positively with parental education (β = 0.06, 95% CI = [0.02,0.09], n = 3,225), and non-significantly with childhood family income (β = 0.2, 95% CI = [0.01,0.07], n = 3,272)
Dietze, P., & Knowles, E. D. (2021). Social Class Predicts Emotion Perception and Perspective-Taking Performance in Adults. *Personality and Social Psychology Bulletin, 47*(1), 42-56. https://doi.org/10.1177/0146167220914116	Study 1A (N = 300<br>Study 1B (N = 451	*Study 1A and 1B*<br>RMET (Baron-Cohen et al., 2001)	*Study 1A and 1B*<br>"People talk about social classes such as the poor, the working class, the middle class, the upper-middle class, and the upper class. Which of these classes would you say you belong to?" (Jackman & Jackman, 1983)	*Study 1A*<br>r = −.169, p = .003, 95% confidence interval [CI] = [−0.277, −0.057]<br>*Study 1B*<br>r = −.250, p = 7.30 × 10−8, 95% CI = [−0.335, −0.162]
Schmalor, A., & Heine, S. J. (2022). Subjective Economic Inequality Decreases Emotional Intelligence, Especially for People of High Social Class. *Social Psychological and Personality Science, 13*(2), 608-617. https://doi.org/10.1177/19485506211024024	Study 2a (N = 284)	RMET (Baron-Cohen et al., 2001)	Subjective SC: participants indicated their subjective SES (Adler et al., 2000) on a ladder with 10 rungs that indicated one's relative standing in society; participants indicated which of five social classes they thought they belonged to (i.e., poor, working class, middle class, upper middle class, and upper class; Jackman & Jackman, 1983	β = −3.11, p < .001, 95%CI = [−4.08, −2.13]
Monroy, M., Cowen, A. S., & Keltner, D. (2022). Intersectionality in emotion signaling and recognition: The influence of gender, ethnicity, and social class. *Emotion, 22*(8),	N = 555	Full body expressions for 34 different emotions	MacArthur scale of subjective social status (Adler et al., 2000)	The social class of the encoder predicted overall reliability in emotion recognition, such that lower class individuals were

1980–1988. https://doi.org/10.1037/emo0001082				more reliably judged in their full-body expression of 34 emotions (β = -.21, p = .007). Evidence was also found for the influence of social class upon our decoders' ability to recognize emotion (β = -.22, p , .001). In this analysis, lower class individuals proved to be better judges of others' full-body expressions of 34 emotion states
Kafetsios, K., & Hess, U. (2022). Personality and the accurate perception of facial emotion expressions: What is accuracy and how does it matter? *Emotion*, *22*(1), 100–114. https://doi.org/10.1037/emo0001034	Study 7 (N = 525)	ACE-Faces short version	MacArthur scale of Subjective Social Status (Adler et al., 2000)	ACE bias (but not accuracy) was a positive predictor of higher subjective social status (β = .15, t = 2.42, p , .01)
Brener, S. A., Frankenhuis, W. E., Young, E. S., & Ellis, B. J. (2024). Social class, sex, and the ability to recognize emotions: The main effect is in the interaction. *Personality and Social Psychology Bulletin*, *50*(8), 1197–1210. https://doi.org/10.1177/01461672231159775	Study 1 (N = 418);<br>Study 2 (N = 745);<br>Study 3 (N = 381)	RMET (Baron-Cohen et al., 2001);<br>the visual portion of the CAM (Golan et al., 2006)	Two items for Subjective SC:<br>"People talk about social classes such as the poor, the working class, the middle class, the upper-middle class, and the upper class. Which of these classes would you say you belong to?" (Jackman & Jackman, 1983);<br>McArthur ladder (Adler et al., 2000)<br>Objective SC:<br>average of mother and father education	Subjective social class was inversely related to performance on the RMET and the CAM;<br>Multivariate analyses in Studies 1 and 2 revealed that (a) objective social class (i.e., parental education) did not significantly predict emotion recognition (though p = .073 in Study 2), and (b) subjective social class remained a significant predictor of emotion recognition after controlling for objective social class;

				The association between SSC and emotion recognition occurred only in males
Engstrom, H. R., & Laurin, K. (2024). Lower social class, better social skills? A registered report testing diverging predictions from the rank and cultural approaches to social class. *Journal of Experimental Social Psychology*, *111*, 1–15. https://doi.org/10.1016/j.jesp.2023.104577	N = 1139	RMET (Baron-Cohen et al., 2001);<br>The miniPONS (Bänziger et al., 2011)	Subjective SES:<br>ladder with 10 rungs representing the socioeconomic hierarchy (Adler et al., 1994); participants indicated their social class category (poor / working class / middle class / upper-middle class / upper class; Dietze & Knowles, 2021).<br>Objective SES:<br>highest educational degree; household income	Lower subjective rank, social class cultural group, and income—but not education—all predict better empathic accuracy;<br>subjective rank more strongly predicts empathic accuracy compared to SES cultural group (consistent with the rank approach), but childhood SES more strongly predicts empathic accuracy than adulthood SES (consistent with the cultural approach).
