# Supplementary material for: Higher social class is associated with higher contextualized emotion recognition accuracy across cultures
Source: PLoS One. 2025 May 13;20(5):e0323552. doi: 10.1371/journal.pone.0323552 (PMC12074547; doi:10.1371/journal.pone.0323552)
Supplement: S4 Table — (PDF) [file pone.0323552.s007.pdf]

Table S4

Tucker's Phi Coefficients for Self-Construal Scale Factors across Countries

Country	Factor 1 (Independent SC)	Factor 2 (Interdependent SC)
China	.919	.962
Spain	.928	.954
Germany	.895	.947
Greece	.904	.928
India	.921	.848
Ireland	.953	.922
Italy	.970	.961
Japan	.955	.940
Poland	.922	.958
Turkey	.960	.966
USA	.965	.970
UK	.986	.986
