# Supplementary material for: Higher social class is associated with higher contextualized emotion recognition accuracy across cultures
Source: PLoS One. 2025 May 13;20(5):e0323552. doi: 10.1371/journal.pone.0323552 (PMC12074547; doi:10.1371/journal.pone.0323552)
Supplement: S5 Table — (PDF) [file pone.0323552.s008.pdf]

Table S5a (Accuracy – Happy)

Multilevel model of relationships between Subjective Social Status (SSS) and ACE accuracy happy

	Coef.	SE	t-value
Intercept γ_{00}	4.319	.079	54.54**
SSS γ_{10}	.019	.009	1.94^
Gender. γ_{20}	-.016	.06	-.267
Age γ_{30}	.004	.002	1.95^
Bias happy γ_{40}	-.278	.076	-3.636**

Note: Coefficients in bold are described in the results section. Gender coded -1 = males , 1 = females * p < .05, ** p < .01, *** p < .001, ^ < .08

Table S5b (Accuracy – Happy)

Multilevel model of relationships between Subjective Social Status (SSS) and ACE accuracy happy as a function of countries' Long Term Orientation (LTO), Relational Mobility (RM) and GINI

					GINI				LTO				RM	
	Coef.	SE	t-value		Coef.	SE	t-value		Coef.	SE	t-value		Coef.	SE	t-value
Intercept γ_{00}	4.323	.098	44.015***	γ_{01}	.0003	.0006	.96	γ_{02}	**-.007**	**.0001**	**-3.852*** **	γ_{03}	-.059	.053	-1.113
SSS γ_{10}	.018	.012	1.502	γ_{11}	-.0001	.0001	-.771	γ_{12}	**.0001**	**.0003**	**4.506** **	**γ_{13}**	.0006	.012	.958
Gender. γ_{20}	-.018	.060	-.302												
Age γ_{30}	.004	.002	2.004^												
Bias happy γ_{40}	-.280	.080	-3.467**												

Note: Coefficients in bold are described in the results section. Gender coded -1 = males , 1 = females * p < .05, ** p < .01, *** p < .001, ^ < .08
