# Supplementary material for: Higher social class is associated with higher contextualized emotion recognition accuracy across cultures
Source: PLoS One. 2025 May 13;20(5):e0323552. doi: 10.1371/journal.pone.0323552 (PMC12074547; doi:10.1371/journal.pone.0323552)
Supplement: S6 Table — (PDF) [file pone.0323552.s009.pdf]

Table S6a (Accuracy – Angry)

Multilevel model of relationships between Subjective Social Status (SSS) and ACE accuracy angry

	Coef.	SE	t-value
Intercept $_{\gamma 00}$	2.884	.086	33.245**
SSS $_{\gamma 10}$	.020	.01	1.968^
Gender. $_{\gamma 20}$	.144	.051	2.806*
Age $_{\gamma 30}$	-.008	.001	-4.534**
Bias angry $_{\gamma 40}$	.476	.052	9.040**

Note: Coefficients in bold are described in the results section. Gender coded -1 = males , 1 = females * *p* < .05, ** *p* < .01, *** *p* < .001, ^ < .08

Table S6b (Accuracy – Angry)

Multilevel model of relationships between Subjective Social Status (SSS) and ACE accuracy angry as a function of countries' Long Term Orientation (LTO), Relational Mobility (RM) and GINI

					GINI				LTO				RM		
	Coef.	SE	t-value		Coef.	SE	t-value		Coef.	SE	t-value		Coef.	SE	t-value
Intercept $_{\gamma 00}$	2.891	.126	22.855***	γ_{01}	**-.01**	**.00**	**-4.22****	γ_{02}	-.003	.002	-1.33	γ_{03}	**.340**	**.112**	**3.039***
SSS $_{\gamma 10}$	.021	.007	3.030*	γ_{11}	-.003	.001	-2.205^	γ_{12}	**.001**	**.0003**	**3.290***	γ_{13}	**.028**	**.009**	**2.974***
Gender. $_{\gamma 20}$	.140	.051	2.703*												
Age $_{\gamma 30}$	-.006	.051	-2.777*												
Bias angry $_{\gamma 40}$	.478	.052	9.075***												

Note: Coefficients in bold are described in the results section. Gender coded -1 = males . 1 = females * *p* < .05, ** *p* < .01, *** *p* < .001, ^ < .08
