# Supplementary material for: Higher social class is associated with higher contextualized emotion recognition accuracy across cultures
Source: PLoS One. 2025 May 13;20(5):e0323552. doi: 10.1371/journal.pone.0323552 (PMC12074547; doi:10.1371/journal.pone.0323552)
Supplement: S9 Table — (PDF) [file pone.0323552.s010.pdf]

Table S9a (Accuracy – Congruent)

Multilevel model of relationships between Subjective Social Status (SSS) and ACE congruent accuracy

	Coef.	SE	t-value
Intercept γ_{00}	2.977	.085	34.910***
SSS γ_{10}	.021	.009	2.239*
Gender. γ_{20}	.187	.042	3.518**
Age γ_{30}	-.007	.001	-2.991*
Bias congruent γ_{40}	.545	.050	13.724***

Note: Coefficients in bold are described in the results section. Gender coded -1 = males , 1 = females * $p < .05$, ** $p < .01$, *** $p < .001$

Table S9b (Accuracy – Congruent)

Multilevel model of relationships between Subjective Social Status (SSS) and ACE congruent accuracy as a function of countries' Long Term Orientation (LTO), Relational Mobility (RM) and GINI

					GINI				LTO				RM		
	Coef.	SE	t-value		Coef.	SE	t-value		Coef.	SE	t-value		Coef.	SE	t-value
Intercept γ_{00}	2.981	.116	25.548***	γ_{01}	**-.027**	**.008**	**-3.301***	γ_{02}	**-.005**	**.001**	**-3.473****	γ_{03}	-.063	.065	-.968
SSS γ_{10}	.023	.005	4.473**	γ_{11}	-.001	.000	-1.509	γ_{12}	**.0008**	**.0001**	**5.761*****	**γ_{13}**	**-.016**	**.004**	**-3.653****
Gender. γ_{20}	.185	.053	3.466**												
Age γ_{30}	-.008	.001	-5.203**												
Bias congruent γ_{40}	.547	.040	13.653***												

Note: Coefficients in bold are described in the results section. Gender coded -1 = males . 1 = females * $p < .05$, ** $p < .01$, *** $p < .001$, ^ < .08
