# Supplementary material for: Higher social class is associated with higher contextualized emotion recognition accuracy across cultures
Source: PLoS One. 2025 May 13;20(5):e0323552. doi: 10.1371/journal.pone.0323552 (PMC12074547; doi:10.1371/journal.pone.0323552)
Supplement: S10 Table — (PDF) [file pone.0323552.s011.pdf]

Table S10a (Accuracy – Incongruent)

Multilevel model of relationships between Subjective Social Status (SSS) and ACE incongruent accuracy

	Coef.	SE	t-value
Intercept γ_{00}	3.452	.036	95.354***
SSS γ_{10}	.012	.008	1.430
Gender. γ_{20}	.082	.041	2.012^
Age γ_{30}	-.001	.001	-1.540
Bias Incongruent γ_{40}	.466	.045	10.337***

Note: Coefficients in bold are described in the results section. Gender coded -1 = males , 1 = females * $p < .05$, ** $p < .01$, *** $p < .001$, ^ < .08

Table S10b (Accuracy – Incongruent)

Multilevel model of relationships between Subjective Social Status (SSS) and ACE congruent accuracy as a function of countries' Long Term Orientation (LTO), Relational Mobility (RM) and GINI

					GINI				LTO				RM		
	Coef.	SE	t-value		Coef.	SE	t-value		Coef.	SE	t-value		Coef.	SE	t-value
Intercept γ_{00}	3.456	.043	80.023***	γ_{01}	**-.012**	**.004**	**-2.594***	γ_{02}	-.0001	.001	-.073	γ_{03}	.03	.05	.61
SSS γ_{10}	.012	.008	1.572	γ_{11}	**-.003**	**.001**	**-3.158****	γ_{12}	.0004	.0002	1.577	$\boldsymbol{\gamma_{13}}$	**-.018**	**.0007**	**-2.401***
Gender. γ_{20}	.080	.041	1.941												
Age γ_{30}	-.001	.0008	-1.751												
Bias Incongruent γ_{40}	.466	.044	10.375***												

Note: Coefficients in bold are described in the results section. Gender coded -1 = males . 1 = females * $p < .05$, ** $p < .01$, *** $p < .001$, ^ < .08
