# Supplementary material for: Higher social class is associated with higher contextualized emotion recognition accuracy across cultures
Source: PLoS One. 2025 May 13;20(5):e0323552. doi: 10.1371/journal.pone.0323552 (PMC12074547; doi:10.1371/journal.pone.0323552)
Supplement: S12 Table — (PDF) [file pone.0323552.s012.pdf]

Table S12a (Bias – Happy)

Multilevel model of relationships between Parental Education Level (PEL) and ACE bias happy

	Coef.	SE	t-value
Intercept γ_{00}	1.686	.040	41.429***
Parental Education Level γ_{10}	-.006	.002	-2.798*
Gender. γ_{20}	-.057	.013	-4.386**
Age γ_{30}	-.006	.002	-2.798*
ACCHAP γ_{40}	.293	.019	14.965***

Note: Coefficients in bold are described in the results section. Gender coded -1 = males , 1 = females * $p < .05$, ** $p < .01$, *** $p < .001$

Table S12b (Bias – Happy)

Multilevel model of relationships between Parental Education Level (PEL) and ACE bias happy as a function of countries' Long Term Orientation (LTO), Relational Mobility (RM) and GINI

					GINI				LTO				RM		
	Coef.	SE	t-value		Coef.	SE	t-value		Coef.	SE	t-value		Coef.	SE	t-value
Intercept γ_{00}	1.685	.029	56.934***	γ_{01}	-.006	.003	-2.124	γ_{02}	-.001	.0007	-1.84	γ_{03}	**-.231**	**.032**	**-7.213*** **
Gender. γ_{10}	-.057	.013	-4.399***												
Age γ_{20}	-.002	.001	-1.864												
Parental Education Level γ_{30}	-.006	.002	-2.801*	γ_{31}	.0007	.0003	-2.235^	γ_{32}	.000	.000	.603	$\boldsymbol{\gamma_{33}}$	-.004	.0004	-.999
ACCHAP γ_{40}	-.080	.025	-3.117*												

Note: Coefficients in bold are described in the results section. Gender coded -1 = males , 1 = females * $p < .05$, ** $p < .01$, *** $p < .001$, ^ < .031
