# Supplementary material for: Higher social class is associated with higher contextualized emotion recognition accuracy across cultures
Source: PLoS One. 2025 May 13;20(5):e0323552. doi: 10.1371/journal.pone.0323552 (PMC12074547; doi:10.1371/journal.pone.0323552)
Supplement: S13 Table — (PDF) [file pone.0323552.s013.pdf]

Table S13a (Bias – Angry)

Multilevel model of relationships between Parental Education Level (PEL) and ACE bias angry

	Coef.	SE	t-value
Intercept γ_{00}	1.686	.040	41.429***
Parental Education Level γ_{10}	-.006	.002	-2.798*
Gender. γ_{20}	-.057	.013	-4.386**
Age γ_{30}	-.006	.002	-2.798*
Accuracy Angry γ_{40}	.293	.019	14.965***

Note: Coefficients in bold are described in the results section. Gender coded -1 = males , 1 = females * p < .05, ** p < .01, *** p < .001

Table S13b (Bias – Angry)

Multilevel model of relationships between Parental Education Level (PEL) and ACE bias angry as a function of countries' Long Term Orientation (LTO), Relational Mobility (RM) and GINI

					GINI				LTO				RM		
	Coef.	SE	t-value		Coef.	SE	t-value		Coef.	SE	t-value		Coef.	SE	t-value
Intercept γ_{00}	2.144	.047	45.305***	γ_{01}	-.009	.004	-1.975	γ_{02}	**-.003**	**.001**	**-3.050***	γ_{03}	**-.162**	**.017**	**-9.452***
Gender. γ_{10}	-.098	.018	-5.436***												
Age γ_{20}	.001	.001	.793												
Parental Education Level γ_{30}	-.01	.004	-2.359*	γ_{31}	-.000	.000	-1.088	γ_{32}	.000	.000	.488	$\boldsymbol{\gamma_{33}}$	.012	.012	1.022
Accuracy angry γ_{40}	.156	.021	7.158***												

Note: Coefficients in bold are described in the results section. Gender coded -1 = males , 1 = females * p < .05, ** p < .01, *** p < .001, ^ < .031
