# Supplementary material for: Higher social class is associated with higher contextualized emotion recognition accuracy across cultures
Source: PLoS One. 2025 May 13;20(5):e0323552. doi: 10.1371/journal.pone.0323552 (PMC12074547; doi:10.1371/journal.pone.0323552)
Supplement: S14 Table — (PDF) [file pone.0323552.s014.pdf]

Table S14a (Bias – Sad)

Multilevel model of relationships between Parental Education Level (PEL) and ACE bias sad

	Coef.	SE	t-value
Intercept γ_{00}	1.686	.040	41.429***
Parental Education Level γ_{10}	-.006	.002	-2.798*
Gender. γ_{20}	-.057	.013	-4.386**
Age γ_{30}	-.006	.002	-2.798*
Accuracy sad γ_{40}	.293	.019	14.965***

Note: Coefficients in bold are described in the results section. Gender coded -1 = males , 1 = females * *p* < .05, ** *p* < .01, *** *p* < .001

Table S14b (Bias – Sad)

Multilevel model of relationships between Parental Education Level (PEL) and ACE bias sad as a function of countries' Long Term Orientation (LTO), Relational Mobility (RM) and GINI

					**GINI**				**LTO**				**RM**		
	Coef.	SE	t-value		Coef.	SE	t-value		Coef.	SE	t-value		Coef.	SE	t-value
Intercept γ_{00}	2.060	.059	34.810***	γ_{01}	-.003	.005	-.746	γ_{02}	-.001	.001	-.745	γ_{03}	**-.425**	**.036**	**-11.697*** **
Gender. γ_{10}	-.120	.018	-6.619***												
Age γ_{20}	.002	.002	1.083												
Parental Education Level γ_{30}	-.006	.005	-1.304	γ_{31}	.000	.000	-2.148^	γ_{32}	.000	.000	.075	$\boldsymbol{\gamma_{33}}$	-.013	.010	-1.279
Accuracy sad γ_{40}	.149	.013	10.720***												

Note: Coefficients in bold are described in the results section. Gender coded -1 = males , 1 = females * *p* < .05, ** *p* < .01, *** *p* < .001, ^ < .031
