# Supplementary material for: Higher social class is associated with higher contextualized emotion recognition accuracy across cultures
Source: PLoS One. 2025 May 13;20(5):e0323552. doi: 10.1371/journal.pone.0323552 (PMC12074547; doi:10.1371/journal.pone.0323552)
Supplement: S15 Table — (PDF) [file pone.0323552.s015.pdf]

Table S15a (Bias – Disgust)

Multilevel model of relationships between Parental Education Level (PEL) and ACE bias disgust

	Coef.	SE	t-value
Intercept γ_{00}	1.686	.040	41.429***
Parental Education Level γ_{10}	-.006	.002	-2.798*
Gender. γ_{20}	-.057	.013	-4.386**
Age γ_{30}	-.006	.002	-2.798*
Accuracy disgust γ_{40}	.293	.019	14.965***

Note: Coefficients in bold are described in the results section. Gender coded -1 = males , 1 = females * $p < .05$, ** $p < .01$, *** $p < .001$

Table S15b (Bias – Disgust)

Multilevel model of relationships between Parental Education Level (PEL) and ACE bias disgust as a function of countries' Long Term Orientation (LTO), Relational Mobility (RM) and GINI

					GINI				LTO				RM	
	Coef.	SE	t-value		Coef.	SE	t-value		Coef.	SE	t-value		Coef.	SE	t-value
Intercept γ_{00}	2.021	.052	38.242***	γ_{01}	-.008	.006	-1.230	γ_{02}	-.001	.001	-.099	γ_{03}	**-.283**	**.051**	**-5.513*** **
Gender. γ_{10}	-.128	.020	-6.64***												
Age γ_{20}	.002	.002	1.15												
Parental Education Level γ_{30}	-.013	.005	-2.48*	γ_{31}	.0002	.0008	-.243	γ_{32}	.0001	.0001	-1.102	$\boldsymbol{\gamma_{33}}$	-.010	.009	-1.031
Accuracy disgust γ_{40}	.088	.017	5.014***												

Note: Coefficients in bold are described in the results section. Gender coded -1 = males , 1 = females * $p < .05$, ** $p < .01$, *** $p < .001$, ^ < .031
