# Supplementary material for: Higher social class is associated with higher contextualized emotion recognition accuracy across cultures
Source: PLoS One. 2025 May 13;20(5):e0323552. doi: 10.1371/journal.pone.0323552 (PMC12074547; doi:10.1371/journal.pone.0323552)
Supplement: S16 Table — (PDF) [file pone.0323552.s016.pdf]

Table S16a (Bias – Congruent)

Multilevel model of relationships between Parental Education Level (PEL) and ACE bias congruent

	Coef.	SE	t-value
Intercept γ_{00}	1.686	.040	41.429***
Parental Education Level γ_{10}	-.006	.002	-2.798*
Gender. γ_{20}	-.057	.013	-4.386**
Age γ_{30}	-.006	.002	-2.798*
Accuracy congruent γ_{40}	.293	.019	14.965***

Note: Coefficients in bold are described in the results section. Gender coded -1 = males , 1 = females * p < .05, ** p < .01, *** p < .001

Table S16b (Bias – Congruent)

Multilevel model of relationships between Parental Education Level (PEL) and ACE bias congruent as a function of countries' Long Term Orientation (LTO), Relational Mobility (RM) and Gini

					GINI				LTO				RM		
	Coef.	SE	t-value		Coef.	SE	t-value		Coef.	SE	t-value		Coef.	SE	t-value
Intercept γ_{00}	2.060	.059	34.349***	γ_{01}	-.008	.002	-3.094	γ_{02}	-.001	.001	-1.012	γ_{03}	**-.121**	**.017**	**-6.973*****
Gender. γ_{10}	-.135	.021	-6.300***												
Age γ_{20}	.001	.001	.531												
Accuracy congruent γ_{30}	.255	.019	12.941***												
Parental Education Level γ_{40}	-.012	.004	-2.923*	γ_{41}	.0004	.0004	-.962	γ_{42}	**.0001**	**.004**	**-2.923***	**γ_{43}**	.0004	.0004	-.962

Note: Coefficients in bold are described in the results section. Gender coded -1 = males , 1 = females * p < .05, ** p < .01, *** p < .001, ^ < .031
