# Supplementary material for: Higher social class is associated with higher contextualized emotion recognition accuracy across cultures
Source: PLoS One. 2025 May 13;20(5):e0323552. doi: 10.1371/journal.pone.0323552 (PMC12074547; doi:10.1371/journal.pone.0323552)
Supplement: S17 Table — (PDF) [file pone.0323552.s017.pdf]

Table S17a (Bias – Incongruent)

Multilevel model of relationships between Parental Education Level (PEL) and ACE bias incongruent

	Coef.	SE	t-value
Intercept γ_{00}	1.686	.040	41.429***
Parental Education Level γ_{10}	-.006	.002	-2.798*
Gender. γ_{20}	-.057	.013	-4.386**
Age γ_{30}	-.006	.002	-2.798*
Accuracy congruent γ_{40}	.293	.019	14.965***

Note: Coefficients in bold are described in the results section. Gender coded -1 = males , 1 = females * $p < .05$, ** $p < .01$, *** $p < .001$

Table S17b (Bias – Incongruent)

Multilevel model of relationships between Parental Education Level (PEL) and ACE bias incongruent as a function of countries' Long Term Orientation (LTO), Relational Mobility (RM) and Gini

				GINI				LTO				RM		
	Coef.	SE	t-value		Coef.	SE	t-value		Coef.	SE	t-value		Coef.	SE	t-value
Intercept γ_{00}	1.960	.037	52.244***	γ_{01}	-.005	.004	-1.27	γ_{02}	.001	.001	.725	γ_{03}	**-.335**	**.037**	**-8.907*** **
Gender. γ_{10}	-.080	.016	-4.756***												
Age γ_{20}	.001	.001	0.845												
Accuracy congruent γ_{40}	.211	.017	12.322***												
Parental Education Level γ_{40}	-.005	.004	-1.223	γ_{41}	**-.001**	**.0004**	**-3.362***	γ_{42}	.000	.004	-1.223	γ_{43}	**-.016**	**.005**	**-2.773***

Note: Coefficients in bold are described in the results section. Gender coded -1 = males , 1 = females * $p < .05$, ** $p < .01$, *** $p < .001$, ^ < .031
