# Supplementary material for: Higher social class is associated with higher contextualized emotion recognition accuracy across cultures
Source: PLoS One. 2025 May 13;20(5):e0323552. doi: 10.1371/journal.pone.0323552 (PMC12074547; doi:10.1371/journal.pone.0323552)
Supplement: S18 Table — (PDF) [file pone.0323552.s018.pdf]

Table S18a

Multilevel model of relationships between Parental Education Level (PEL) and ACE bias

	Coef.	SE	t-value
Intercept γ_{00}	2.029	.055	36.421***
Gender. γ_{20}	-.115	.020	-5.751***
Age γ_{30}	.001	.001	.506
Accuracy γ_{40}	.293	.019	14.965***
Parental Education Level γ_{10}	**-.010**	**.004**	**-2.652***

Note: Coefficients in bold are described in the results section. Gender coded -1 = males , 1 = females * $p < .05$, ** $p < .01$, *** $p < .001$, ^ < .08

Table S18b

Multilevel model of relationships between Parental Education Level (PEL) and ACE bias as a function of countries' Long Term Orientation (LTO), Relational Mobility (RM) and GINI

					Gini				LTO				RM		
	Coef.	SE	t-value		Coef.	SE	t-value		Coef.	SE	t-value		Coef.	SE	t-value
Intercept γ_{00}	2.029	.049	40.885***	γ_{01}	**-.009**	**.003**	**-2.723***	γ_{02}	-.000	.001	-.298	γ_{03}	**-.125**	**.021**	**-5.813*****
Gender. γ_{10}	-.116	.020	-5.541***												
Age γ_{20}	.001	.001	0.576												
Accuracy γ_{30}	.293	.019	15.148***												
Parental Education Level γ_{40}	**-.01**	**.004**	**-2.619***	γ_{41}	.0008	.0005	-1.617	γ_{42}	-.0002	.004	-1.998^	γ_{43}	-.008	.008	-.949

Note: Coefficients in bold are described in the results section. Gender coded -1 = males , 1 = females * $p < .05$, ** $p < .01$, *** $p < .001$, ^ < .031
