# Supplementary material for: Higher social class is associated with higher contextualized emotion recognition accuracy across cultures
Source: PLoS One. 2025 May 13;20(5):e0323552. doi: 10.1371/journal.pone.0323552 (PMC12074547; doi:10.1371/journal.pone.0323552)
Supplement: S21 Table — (PDF) [file pone.0323552.s019.pdf]

Table S21
Predicting ACE Accuracy Rates across each of the 12 cultures

	USA			Germany			Greece			UK			Spain			India		
	β	*t*	*p*	*β*	*t*	*p*	*β*	*t*	*p*	*β*	*t*	*p*	*β*	*t*	*p*	*β*	*t*	*p*
Model 1																		
Constant		9.10	.000		7.58	.000		9.59	.000		5.64	.000		6.04	.000		6.13	.000
Gender	0.22	3.66	.000	0.04	0.68	.500	-0.05	-0.85	.397	0.15	2.22	.027	0.24	3.39	.001	0.16	2.05	.042
SSS	-0.01	-0.11	.911	0.13	2.10	.037	0.16	2.48	.014	-0.01	-0.16	.873	-0.03	-0.41	.680	0.03	0.39	.694
Age	-0.15	-2.40	.017	-0.15	-2.32	.021	-0.10	-1.62	.107	-0.03	-0.50	.617	-0.01	-0.21	.837	0.01	0.13	.899
Bias	0.47	7.86	.000	0.43	6.94	.000	0.40	6.34	.000	0.38	5.74	.000	0.36	5.12	.000	0.36	4.55	.000
Model 2																		
Constant		8.68	.000		7.45	.000		9.35	.000		5.62	.000		6.13	.000		5.76	.000
Gender	0.23	3.44	.001	0.04	0.67	.503	-0.07	-1.10	.271	0.16	1.94	.053	0.24	3.46	.001	0.17	2.11	.036
SSS	-0.01	-0.14	.891	0.13	1.84	.067	0.20	2.86	.005	-0.02	-0.23	.818	-0.03	-0.42	.678	0.04	0.51	.608
Age	-0.15	-2.40	.017	-0.15	-2.32	.022	-0.11	-1.74	.084	-0.03	-0.50	.617	-0.01	-0.17	.863	0.01	0.13	.897
Bias	0.47	7.82	.000	0.43	6.87	.000	0.40	6.42	.000	0.38	5.70	.000	0.35	5.03	.000	0.36	4.57	.000
Gender x SSS	0.02	0.35	.723	0.00	0.02	.985	-0.10	-1.45	.149	0.02	0.17	.868	0.17	2.48	.014	-0.04	-0.54	.588
R^2	.26			.21			.21			.17			.18			.14		
F (5,145-228)	15.50			11.07			10.80			7.65			7.89			4.81		

Note. SSS = Subjective social status

Table S21 (continued)

	Ireland			Italy			Japan			Poland			Turkey			China		
	β	*t*	*P*	*β*	*t*	*P*	*β*	*t*	*p*	*β*	*T*	*p*	*β*	*t*	*p*	*β*	*t*	*p*
Model 1																		
Constant		8.52	.000		7.63	.000		3.59	.000		5.09	.000		2.78	.006		3.33	.001
Gender	0.31	3.97	.000	0.12	2.30	.022	0.06	1.03	.303	0.32	4.76	.000	0.09	1.47	.144	-0.22	-3.77	.000
SSS	-0.12	-1.55	.122	0.07	1.38	.168	0.14	2.21	.028	0.06	0.88	.381	0.01	0.18	.860	0.10	1.76	.081
Age	-0.05	-0.63	.528	-0.12	-2.33	.020	0.01	0.12	.908	-0.06	-0.95	.343	0.06	0.98	.328	0.09	1.61	.108
Bias	0.26	3.32	.001	0.43	8.07	.000	0.54	8.69	.000	0.33	4.90	.000	0.44	6.94	.000	0.51	9.02	.000
Model 2																		
Constant		8.13	.000		7.48	.000		3.67	.000		5.05	.000		2.67	.008		3.20	.002
Gender	0.31	4.01	.000	0.12	2.28	.024	0.04	0.61	.545	0.33	4.68	.000	0.09	1.32	.188	-0.23	-3.99	.000
SSS	-0.10	-1.19	.235	0.07	1.28	.201	0.13	2.10	.038	0.06	0.84	.401	0.01	0.17	.861	0.13	2.17	.032
Age	-0.05	-0.68	.497	-0.12	-2.33	.021	0.01	0.13	.898	-0.07	-0.95	.342	0.06	0.98	.330	0.09	1.64	.102
Bias	0.26	3.31	.001	0.43	8.01	.000	0.54	8.69	.000	0.33	4.89	.000	0.44	6.93	.000	0.52	9.12	.000
Gender x SSS	-0.06	-0.69	.493	0.00	-0.02	.987	0.06	0.82	.413	0.02	0.34	.732	0.00	-0.01	.989	-0.09	-1.51	.132
R^2	.17			.19			.32			.19			.20			.35		
F *(5,145-228)*	5.78			14.65			16.77			8.69			9.81			21.66		
