# Supplementary material for: Higher social class is associated with higher contextualized emotion recognition accuracy across cultures
Source: PLoS One. 2025 May 13;20(5):e0323552. doi: 10.1371/journal.pone.0323552 (PMC12074547; doi:10.1371/journal.pone.0323552)
Supplement: S22 Table — (PDF) [file pone.0323552.s020.pdf]

Table S22

Predicting ACE bias rates across each of the 12 cultures

	USA			Germany			Greece			UK			Spain			India		
	β	*t*	*p*	*β*	*T*	*p*	*B*	*t*	*p*	*β*	*t*	*p*	*β*	*t*	*p*	*β*	*t*	*p*
Model 1																		
Constant		2.74	.007		4.14	.000		3.29	.001		3.41	.001		3.68	.000		5.48	.000
Gender	-0.17	-2.88	.004	-0.06	-0.92	.358	-0.03	-0.46	.649	-0.07	-1.01	.312	-0.25	-3.53	.001	-0.17	-2.21	.029
SSS	0.16	2.78	.006	-0.14	-2.21	.029	0.02	0.25	.800	0.04	0.63	.531	0.01	0.18	.858	-0.06	-0.80	.426
Age	0.18	2.99	.003	0.10	1.55	.123	0.09	1.35	.178	-0.02	-0.25	.805	0.00	0.07	.945	-0.18	-2.37	.019
Accuracy	0.46	7.86	.000	0.44	6.94	.000	0.41	6.34	.000	0.39	5.74	.000	0.36	5.12	.000	0.34	4.55	.000
Model 2																		
Constant		3.07	.002		3.76	.000		3.34	.001		3.45	.001		3.64	.000		5.61	.000
Gender	-0.23	-3.43	.001	-0.05	-0.77	.443	-0.01	-0.21	.834	-0.03	-0.37	.713	-0.25	-3.52	.001	-0.19	-2.44	.016
SSS	0.16	2.89	.004	-0.08	-1.13	.260	-0.02	-0.30	.767	-0.01	-0.12	.901	0.01	0.18	.858	-0.08	-1.06	.292
Age	0.18	2.99	.003	0.09	1.47	.143	0.09	1.46	.147	-0.02	-0.25	.804	0.00	0.07	.946	-0.18	-2.36	.020
Accuracy	0.45	7.82	.000	0.43	6.87	.000	0.41	6.42	.000	0.38	5.70	.000	0.36	5.03	.000	0.34	4.57	.000
Gender x SSS	-0.12	-1.91	.057	-0.12	-1.70	.090	0.09	1.24	.216	0.09	0.87	.387	-0.01	-0.11	.912	0.09	1.18	.238
R^2	.29			.21			.18			.15			.16			.18		
$F_{(5,145-228)}$	18.29			11.19			9.20			6.87			6.67			6.50		

Note. SSS = Subjective social status

Table S22(continued)

	Ireland			Italy			Japan			Poland			Turkey			China		
	β	*t*	*p*	*β*	*t*	*p*	*β*	*t*	*p*	*β*	*t*	*p*	*β*	*t*	*p*	*B*	*t*	*p*
Model 1																		
Constant		4.14	.000		6.64	.000		3.52	.001		5.74	.000		4.70	.000		2.26	.025
Gender	-0.17	-2.04	.043	-0.21	-4.09	.000	-0.13	-2.13	.034	-0.24	-3.30	.001	-0.13	-2.11	.036	0.06	1.01	.312
SSS	0.08	0.98	.328	0.03	0.55	.580	-0.04	-0.62	.536	-0.05	-0.67	.501	0.01	0.17	.864	-0.08	-1.32	.189
Age	-0.01	-0.10	.921	0.08	1.57	.118	0.05	0.82	.413	0.14	2.04	.043	-0.05	-0.80	.423	-0.03	-0.57	.572
Accuracy	0.28	3.32	.001	0.42	8.07	.000	0.54	8.69	.000	0.34	4.90	.000	0.44	6.94	.000	0.55	9.02	.000
Model 2																		
Constant		4.11	.000		6.13	.000		3.35	.001		5.72	.000		4.56	.000		2.34	.020
Gender	-0.17	-2.04	.043	-0.19	-3.78	.000	-0.12	-1.75	.082	-0.23	-3.16	.002	-0.14	-1.95	.053	0.08	1.30	.194
SSS	0.08	0.85	.398	0.06	1.15	.250	-0.04	-0.57	.572	-0.05	-0.68	.499	0.01	0.13	.893	-0.11	-1.79	.075
Age	-0.01	-0.08	.933	0.08	1.50	.133	0.05	0.81	.418	0.14	2.03	.043	-0.05	-0.81	.420	-0.04	-0.61	.543
Accuracy	0.28	3.31	.001	0.41	8.01	.000	0.54	8.69	.000	0.34	4.89	.000	0.44	6.93	.000	0.56	9.12	.000
Gender x SSS	0.02	0.18	.855	-0.09	-1.68	.094	-0.03	-0.45	.650	0.01	0.09	.927	0.01	0.13	.897	0.10	1.62	.107
R^2	.08			.22			.31			.15			.20			.30		
$F_{(5,145-228)}$	2.52			17.19			16.45			6.82			10.31			17.42		

Note. SSS = Subjective social status
