# Supplementary material for: Higher social class is associated with higher contextualized emotion recognition accuracy across cultures
Source: PLoS One. 2025 May 13;20(5):e0323552. doi: 10.1371/journal.pone.0323552 (PMC12074547; doi:10.1371/journal.pone.0323552)
Supplement: S1 File — (PDF) [file pone.0323552.s021.pdf]

Supplemental Material

"Higher social class is associated with higher contextualized emotion recognition accuracy across cultures"

Konstantinos Kafetsios , Ursula Hess, Itziar Alonso-Arbiol, Astrid Schütz, Dritjon Gruda, Kelly Campbell, Bin-Bin Chen, Daniel Dostal, Marco J. Held, Petra Hypsova, Shanmukh Kamble, Takuma Kimura, Alexander Kirchner-Häusler, Marina Kyvelea, Stefano Livi, Eugenia Mandal, Dominika Ochnik, Nektarios Papageorgakopoulos, Martin Seitl, Ezgi Sakman, Nebi Sumer, Filip Sulejmanov, Annalisa Theodorou, & Ayse K. Uskul

Study 1

A. In the first phase of the study, a series of online questionnaires were completed in the University XXX online Platform. Before completing the questionnaires, participants were informed of the aim of the study and given a participant consent form. The order of the questionnaires is shown below.

1. Socioeconomic Status (SES)
   MacArthur Scale of Subjective Socioeconomic Status (Adler et al., 2000).
   Parental Education Status
2. The Individualism-Collectivism Scale (INDCOL, Singelis et al., 1995).
3. Empathy Scale (EQ; Muncer & Ling, 2006).
4. The Experiences in Close Relationships (ECR, Breannan et al., 1998).
5. Faces part of the MSCEIT (Mayer, Salovey, Caruso Emotional Intelligence Test, Mayer et al., 2003).
6. Big Five Inventory-2-Short (Hřebíčková et al., 2020; Soto & John, 2017).
7. Agency and Communion Scale (Li et al., 2007).
8. The Emotion Regulation Questionnaire (ERQ, Gross & John, 2003).
9. Three-Item Loneliness Scale (Hughes et al., 2004).
10. A short version of the Assessment of Contextualised Emotions (ACE-Faces, Hess et al., 2016).

After the participants had completed all the questionnaires, we divided them into dyads based on the same gender and asked them to come to the laboratory of the Psychology Department of the University of XXX

B. Participation in a laboratory task. The laboratory task consisted of four subtasks.

- B1. Completion of the following questionnaires:

1. The State-Trait Anxiety Inventory (STAI-X1, Spielberger et al., 1970).
2. Rosenberg Self Esteem Scale (Rosenberg, 1965).
3. The Psychological Well-Being Scale (PWB, Diener et al., 2009).

- B2. Emotion elicitation task (Levenson et al., 1991). Participants were individually asked to describe different situations from their lives and to show their facial expression (i.e., how they felt in that situation) while being recorded by a camera. A total of four emotional situations were requested in a fixed order: anger, sadness, disgust, and happiness.
- B3. Table talk with the participant's dyad member. Participants had a short conversation with the other person in their dyad using the talk-table method (Gottman et al., 1977). A warm-up phase was conducted before the recorded conversation. In this phase, each participant selected a question from a bowl placed in front of them. In the recorded conversation, both a sad and a happy situation were described. The sequence began with the first participant describing the happy situation, followed by the second participant describing the sad situation. The first participant then continued with the sad situation and the second participant concluded the conversation by describing the happy situation.
- B4. Completion of two additional questionnaires on perceptions of interaction quality.

1. The single-item Inclusion of Other in the Self (IOIS, Aron et al., 1992).
2. The Quality of Social Interaction Rating, (part of the Rochester Social Interaction Record, RIR, Nezlek, 1993).

C. Online completion of the ACE dyad task. Participants received an email in which they were firstly instructed to fill in the ACE-dyads (an ACE generic online task). ACE), which were formed as a modification of the ACE-Faces measure. Participants were asked to rate the emotional expression of the central person on a seven-point scale, considering the same seven emotions used in the ACE-Faces procedure. The difference is that the participant's dyad partner is the central person in the photos. The photos of the central person were obtained from a video recorded during the emotion elicitation phase. The Rekognition service provided by Amazon Web Services (Kretch & Banker, 2024) was used to identify images with the desired emotional expression. Each ACE dyad contains a total of 20 stimuli. Participants only completed the ACE dyad, which included the photo of the person they were interacting with.

D. Diary study. After completing the ACE-dyad, participants were asked to complete the Rochester Interaction Record (RIR; Wheeler & Nezlek, 1977) on ten consecutive days to rate each interaction (lasting at least 10 minutes) they had during the day. In addition to when and with whom, they rated the interaction on 17 seven-point scales measuring the quality and emotional intensity of that interaction. If the participant had not sent a record after 24 hours, they were reminded of the study by an automated message. If participants failed to complete the records, an automatic reminder email was sent. After 10 days of participation in the diary study, each participant received an online package containing the results of some completed questionnaires, an individual 25-second demonstration of their video recordings analysed with Noldus FaceReader software, their heart rate graph during the interaction and

two infographics on the meaning of heart rate and emotional expressions. These materials were provided as a further thank you for participating in such a study.

References

Adler, N. E., Epel, E. S., Castellazzo, G., & Ickovics, J. R. (2000). Relationship of subjective and objective social status with psychological and physiological functioning: preliminary data in healthy white women. *Health Psychology: Official Journal of the Division of Health Psychology, American Psychological Association, 19*(6), 586–592.

Aron, A., Aron, E. N., & Smollan, D. (1992). Inclusion of Other in the Self Scale and the structure of interpersonal closeness. *Journal of Personality and Social Psychology, 63*(4), 596–612. https://doi.org/10.1037/0022-3514.63.4.596

Brennan, K. A., Clark, C. L., & Shaver, P. R. (1998). Self-report measurement of adult attachment: An integrative overview. In J. A. Simpson & W. S. Rholes (Eds.), *Attachment theory and close relationships* (pp. 46–76). The Guilford Press.

Diener, E., Wirtz, D., Biswas-Diener, R., Tov, W., Kim-Prieto, C., Choi, D.-w., & Oishi, S. (2009). New measures of well-being. In E. Diener (Ed.), *Assessing well-being: The collected works of Ed Diener* (pp. 247–266). Springer Science + Business Media. https://doi.org/10.1007/978-90-481-2354-4_12

Gottman, J., Markman, H., & Notarius, C. (1977). The topography of marital conflict: A sequential analysis of verbal and nonverbal behavior. *Journal of Marriage and Family, 39*(3), 461–477. https://doi.org/10.2307/350902

Gross, J. J., & John, O. P. (2003). Individual differences in two emotion regulation processes: Implications for affect, relationships, and well-being. *Journal of Personality and Social Psychology, 85*(2), 348–362. https://doi.org/10.1037/0022-3514.85.2.348

Hess, U., Kafetsios, K., Mauersberger, H., Blaison, C., & Kessler, C. L. (2016). Signal and noise in the perception of facial emotion expressions: From labs to life. *Personality & Social Psychology Bulletin, 42*(8), 1092–1110. https://doi.org/10.1177/0146167216651851

Hřebíčková, M., Jelínek, M., Květon, P., Benkovič, A., Botek, M., Sudzina, F., Soto, C. J., & John, O. P. (2020). Big Five Inventory 2 (BFI-2): Hierarchický model s 15 subškálami. *Československá psychologie, 64*(4), 437-460.

Hughes, M. E., Waite, L. J., Hawkley, L. C., & Cacioppo, J. T. (2004). A short scale for measuring loneliness in large surveys: Results from two population-based studies. *Research on Aging, 26*(6), 655–672. https://doi.org/10.1177/0164027504268574

Kraus, M. W., & Stephens, N. M. (2012). A road map for an emerging psychology of social class. *Social and Personality Psychology Compass, 6*(9), 642–656. https://doi.org/10.1111/j.1751-9004.2012.00453.x

Kretch D, Banker A (2024). *paws: Amazon Web Services Software Development Kit.* R package version 0.5.0, https://CRAN.R-project.org/package=paws

Kretch, D., & Banker, A. (2024). *paws: Amazon Web Services Software Development Kit.* R package version 0.5.0, https://CRAN.R-project.org/package=paws

Levenson, R. W., Carstensen, L. L., Friesen, W. V., & Ekman, P. (1991). Emotion, physiology, and expression in old age. *Psychology and Aging, 6*(1), 28–35. https://doi.org/10.1037/0882-7974.6.1.28

Li, S.-M., Tseng, L.-C., Wu, C.-S., & Chen, C.-J. (2007). *Agency and Communion Scale* [Database record]. APA PsycTests. https://doi.org/10.1037/t50661-000

Mayer, J. D., Salovey, P., Caruso, D. R., & Sitarenios, G. (2003). Measuring emotional intelligence with the MSCEIT V2.0. *Emotion (Washington, D.C.), 3*(1), 97–105. https://doi.org/10.1037/1528-3542.3.1.97

Muncer, S. J., & Ling, J. (2006). Psychometric analysis of the Empathy Quotient (EQ) scale. *Personality and Individual Differences, 40*(6), 1111–1119. https://doi.org/10.1016/j.paid.2005.09.020

Nezlek, J. B. (1993). The stability of social interaction. *Journal of Personality and Social Psychology*, *65*(5), 930–941. https://doi.org/10.1037/0022-3514.65.5.930

Rosenberg, M. (1965). *Rosenberg Self-Esteem Scale* (RSES) [Database record]. APA PsycTests. https://doi.org/10.1037/t01038-000

Seitl, M., Ketner, D., Dostál, D., Tesařík, J., Charvát, M., Babůrek, T., Dolejš, M., & Skoupil, D. (2021). *MindMap Diagnostic Methods: Software for development, administration, and evaluation of psychological tests.* Retrieved from [https://mmdm.upol.cz/].

Singelis, T. M., Triandis, H. C., Bhawuk, D., & Gelfand, M. J. (1995). Horizontal and vertical dimensions of individualism and collectivism: A theoretical and measurement refinement. Cross-Cultural Research: *The Journal of Comparative Social Science*, *29*(3), 240–275. https://doi.org/10.1177/106939719502900302

Soto, C. J., & John, O. P. (2017). Short and extra-short forms of the Big Five Inventory–2: The BFI-2-S and BFI-2-XS. *Journal of Research in Personality*, *68*, 69–81. https://doi.org/10.1016/j.jrp.2017.02.004

Spielberger, C. D., Gorsuch, R. L., & Lushene, R. (1970). *Test Manual for the State-Trait Anxiety Inventory*. Palo Alto, CA: Consulting Psychologists Press.

Wheeler, L., & Nezlek, J. (1977). *Rochester Interaction Record (RIR)* [Database record]. APA PsycTests. https://doi.org/10.1037/t06423-000

Table S1

Descriptives and zero-order correlations among main Study 1 variables

	1	2	3	4	5	6	7	8	9	10	11	12
1 Gender	-	.057	.058	-.024	-.074	.121*	-.018	-.143**	.201**	-.092	.041	-.127*
2 Parental education level		-	.266**	-.330**	.033	.072	-.006	.010	.111*	-.006	.061	.080
3 SSS			-	-.410**	.160**	.147**	-.067	-.032	.180**	.053	.098	.009
4 SES				-	-.083	-.087	.029	-.009	-.184**	.017	-.049	-.116*
5 ACE accuracy					.79	.476**	-.017	-.121*	.075	.088	-.058	.138*
6 ACE bias						.96	-.527**	-.517**	.088	.037	-.086	.173**
7 Hit rates							.72	.284**	-.015	-.063	-.006	-.145**
8 MSCEIT faces								.75	-.071	.001	-.007	-.089
9 Vert. individualism									.81	-.091	.203**	.153**
10 Horiz. collectivism										.72	-.183**	.352**
11 Horiz. individualism											.59	-.186**
12 Vert. collectivism												.63
Mean	1.287	3.789	6.078	2.905	5.192	2.329	0.428	44.547	4.772	6.503	6.451	4.461
SD	0.453	1.331	1.330	0.757	0.755	0.575	0.161	7.762	1.399	1.061	0.982	1.177

Note: SSS = Subjective social status, SES = Socio-Economic Status

Table S2a

Regressing ACE accuracy on Subjective Social Status (SSS)

	β	t	p value
Constant	3.691	16.710	.000
SSS	.102	2.134	.034
Gender	-.144	-3.012	.003
ACE bias	.477	9.932	.000
$F_{(4,334)}$	37.870		
R^2	.256		

Table S2b

Regressing ACE accuracy Single on Subjective Social Status (SSS)

	β	t	p value
Constant	3.739	13.043	.000
SSS	.086	1.769	.078
Gender	-.20	-4.199	.000
Age	.063	1.323	.187
Bias Single	.448	9.237	.000
$F_{(4,334)}$	26.514		
R^2	.240		

Study 2

A. Relational Mobility indices through data collection in Italy, India and Greece

We conducted three online studies in Italy (*N* = 223, *Mage* = 30.5, *SD* = 8.55, 54% females, *MeanRM* = 3.95, *SD* = .71), India (*N* = 252, *Mage* = 31, *SD* = 6.45, 71% females, *MeanRM* = 3.20, SD = .55), and Greece (*N* = 252, *Mage* = 29.80, *SD* = 8.11, 62% females, *MeanRM* = 4.10, *SD* = .71). In each study participants completed the Relational Mobility Scale (Yuki, 2007) and the Experiences in Close Relationships (Fraley et al., 2000) –Revised version. The scales were translated in the local language and the study was approved by the ethics committee of XXX University.

References

Fraley, R. C., Waller, N. G., & Brennan, K. A. (2000). An item response theory analysis of self-report measures of adult attachment. *Journal of Personality and Social Psychology*, *78*, 350–365.

Yuki M, et al. (2007). Development of a Scale to Measure Perceptions of Relational Mobility in Society (Hokkaido University, Sapporo, Japan)

B. Study Protocol

1. ECR-R (Fraley et al., 2000) 36 items
2. Self-construal scale (Singelis, 1994) 24 items
3. "How satisfied are you with your life?" 1 item Question
4. Friends and Family parts of the Intragroup Marginalization Inventory (Castillo et al., 2007). 11 items
5. The State Attachment Scale (Gillath et al., 2009) – 10 items
6. Shifting emphasis on the self or the other I – we – two paragraphs (Brewer & Gardner, 1996)
   Participants were randomly assigned in one of the two conditions
   2 items from Singelis individualism scale as manipulation check
7. Self-other interest scale (Gerbasi & Prentice, 2013) 17 items
8. Well-being (Diener et al. 2009) – 8 items
9. Risk avoidance (from STRAQ-1 Vergara et al., 2019)
10. 3-item loneliness scale (Hughes et al., 2004)

11. Short Assessment of Contextualized Emotions-Faces, 16 items (Kafetsios & Hess, 2022)
12.Demographics (including the MacArthur scale of Subjective Social Status, Adler et al. 1994)

Study start and end dates per country site

	start	end
DE	21-Jul-2020	10-May-2021
GR	13-Jul-2020	17-Dec-2020
UK	14-Oct-2020	13-Mar-2021
ES	31-Oct-2020	14-Mar-2021
IND	01-Nov-2020	31-Jan-2021
IRL	04-Jul-2020	23-Apr-2021
ITL	26-May-2021	01-Jul-2021
JPN	22-Jul-2020	23-Apr-2021
POL	31-Oct-2020	04-Jan-2021
TUR	21-Oct-2020	04-Jan-2021
USA	27-Oct-2020	30-Mar-2021
CHN	17-Jul-2020	01-Jan-2021

C. Construct equivalence test

Before further analysis, SCS construct equivalence was examined to ensure cross-cultural comparability (van de Vijver & Leung, 1997). Tucker's phi coefficients were calculated for each country, after comparison with the norm of the Self-Construals Scale (forced to two factors) and using a Procrustean factor rotation. All values showed fair similarity according to critical values indicated by Lorenzo-Seva and ten Berge (2006): i.e., at least .85 or above (See Table S1).

Table S3

A review of studies on relationships between emotion recognition accuracy and social class

Citation	Sample	Emotion recognition accuracy measure	Social class/status measure	Relevant findings
**Studies that support a positive relationship between Social class/ status and EDA**				
Schmid Mast, M., Jonas, K., & Hall, J. A. (2009). Give a Person Power and He or She Will Show Interpersonal Sensitivity: The Phenomenon and Its Why And When. Journal of Personality and Social Psychology, 97(5), 835–850. https://doi.org/10.1037/a0016234	Study 1 (N = 76); Study 2 (N = 134); Study 3 (N = 96); Study 4 (N = 157)	*Study 1* Participants watched videotaped superior-subordinate interactions and inferred the thoughts and feelings of subordinates – accuracy was rated based on the similarity between their inferences and the subordinates' self-reported thoughts or feelings<br><br>*Study 2* Similar to Study 1 (however, participants also assessed superiors' thoughts and feelings)<br><br>*Study 3* The Diagnostic Analysis of Nonverbal Accuracy 2 (DANVA2) (Nowicki & Duke, 1994)<br><br>*Study 4* Profile of Nonverbal Sensitivity (PONS; Rosenthal et al., 1979)	*Study 1* Assigned high (leader)- or low-power role (leader's assistant)<br><br>*Study 2* Word completion task was used to prime participants with either high or low power or with neutral words<br>*Study 3* Participants were randomly assigned to high-power, low-power, or control conditions<br>*Study 4* Participants were randomly assigned to one of three conditions – egoistic, empathic, or neutral (control) – and asked to take the leader's perspective based on a descriptive paragraph.	*Study 1* There was a significant role main effect in the predicted direction, F(1, 72) = 4.91, p = .03, effect size r = .25, with leaders being more interpersonally sensitive (M = 0.52) than assistants (M = 0.43)<br><br>*Study 2* Significant priming main effect, $F(2, 128) = 2.51$, $p = .042$ (one-tailed), effect size r = .14, showing that participants who were primed with high power were more interpersonally sensitive (M = 0.90) than participants who were primed with low power (M = 0.78) and that neutral participants lay in between (M = 0.84)<br><br>*Study 3* The linear contrast was F(1, 93) = 6.04, p = .05, effect size r = .25. The contrast between high power and control was also significant, F(1, 93) = 5.20, p = .05, effect size r = .23. The low-power and

				control conditions did not differ (F < 1)<br><br>*Study 4*<br>Results showed a main effect of condition, F(2, 153) = 2.49, p = .04 (one-tailed), indicating that participants identifying with an empathic leader were more interpersonally sensitive (M = 0.71) than participants identifying with an egoistic leader (M = 0.68); participants identifying with the leader in the control condition were in between (M = 0.70)
Schmid Mast, M., & Darioly, A. (2014). Emotion recognition accuracy in hierarchical relationships. Swiss Journal of Psychology, 73(2), 69–75. https://doi.org/10.1024/1421-0185/a000124	A group of 142 superior-subordinate dyads of employees from different companies and organizations (total N = 284)	Diagnostic Analysis of Nonverbal Accuracy – Adult Faces (DANVA-AF-2; Nowicki & Duke, 1994)	Actual hierarchical relationship	Status was a significant positive predictor of ERA, showing that superiors were significantly better in ERA than their subordinates
Hall, J. A., Schmid Mast, M., & Latu, I.-M. (2015). The vertical dimension of social relations and accurate interpersonal perception: A meta-analysis. *Journal of Nonverbal Behavior*, *39*(2), 131–163. https://doi.org/10.1007/s10919-014-0205-1	67 independent studies totaling 15,505 participants	Diverse measures of general accuracy defined as perceiving other people's cues	SES (e.g., own or parents' education, score on a social class index)	Higher social status had a non-significant association with ERA but predicted higher interpersonal accuracy overall
Momm, T., Blickle, G., Liu, Y., Wihler, A., Kholin, M., & Menges, J. I. (2015). It pays to have an eye for emotions: Emotion recognition ability indirectly predicts annual income. *Journal of Organizational Behavior*, 36(1), 147–163. https://doi.org/10.1002/job.1975	N = 142 employee–peer–supervisor triads	The Diagnostic Analysis of Nonverbal Accuracy 2 (DANVA2; Nowicki & Carton, 1993; Baum & Nowicki, 1998)	Annual income	ERA was linked to annual income as an objective indicator of career success through the sequential mediation of peer-rated political skill and supervisor-rated interpersonal facilitation. The path coefficient of

				interpersonal facilitation on income was β = .16 (p < .05; R^2 = .47 with all control variables), and the indirect effect was significant (estimate = 0.036; SE = 0.019; 90 percent BC-CI [0.004; 0.068])
Bjornsdottir, R. T., Alaei, R., & Rule, N. O. (2017). The perceptive proletarian: Subjective social class predicts interpersonal accuracy. *Journal of Nonverbal Behavior*, *41*(2), 185-201. https://doi.org/10.1007/s10919-016-0248-6	Study 3 (N = 200)	Study 3 Baron-Cohen et al.'s (2001) RMET & Categorization of greyscale university yearbook portraits of the faces of 60 American male and female undergraduates (primarily Caucasian) who self-identified as Democrats or Republicans used in previous work (Rule and Ambady 2010).	Income as Objective SES indicator<br>MacArthur Scale of Subjective Social Status (Subjective SES) to measure their subjective SES (Adler et al. 2000).	Study 3 Controlling for Subjective SES, and education, rncome was **positively** associated with RMET scores (B = .02, SE = .01, t(193) = 2.12, p = .04)
**Studies that support a Negative relationship between Social status & EDA**				
Kraus, M. W., Côté, S., & Keltner, D. (2010). Social class, contextualism, and empathic accuracy. *Psychological Science*, *21*(11), 1716–1723. https://doi.org/10.1177/0956797610387613	Study 1 (N = 200);<br>Study 2 (N = 106);<br>Study 3 (N = 81)	*Study 1*<br>MSCEIT (the 20-item subscale score for the ability to identify emotions in photographs of human faces)<br>*Study 2*<br>Participants rated their own emotions and estimated their partner's emotions during a hypothetical job interview<br>*Study 3*<br>Mind in the Eyes task (Baron-Cohen et al, 2001)	*Study 1*<br>Educational attainment of participants<br>*Study 2*<br>Participants rated themselves on a ladder that had 10 rungs representing where people stood in the university community<br>*Study 3*<br>Manipulation of social class was adapted from measures of subjective perceptions of socioeconomic rank (Adler et al., 2000; Kraus et al., 2009)	*Study 1*<br>High-school-educated participants scored higher in empathic accuracy than their college-educated counterparts, F(1, 196) = 5.18, p < .05.<br><br>*Study 2*<br>Lower class, as measured by subjective SES, was associated with greater empathic accuracy, r(104) = –.20, p < .05<br><br>*Study 3*<br>Participants experimentally induced to experience lower-class rank were better able than their upper-class-rank counterparts to discern

				emotions from subtle expressions in the eyes, F(1, 74) = 4.48, p < .05.
Bjornsdottir, R. T., Alaei, R., & Rule, N. O. (2017). The perceptive proletarian: Subjective social class predicts interpersonal accuracy. *Journal of Nonverbal Behavior*, *41*(2), 185-201. https://doi.org/10.1007/s10919-016-0248-6	Study 2 (N = 150)<br>Study 3 (N = 200)<br>Study 4 (N = 220)	Study 2 Baron-Cohen et al.'s (2001) RMET; Categorization task: participants view randomly-ordered greyscale images of the eyes of 36 Caucasian men and women and choose the word that best describes what the target is thinking or feeling from a set of four<br>options based on their first impressions.<br>Study 3 Baron-Cohen et al.'s (2001) RMET & Categorization of greyscale university yearbook portraits of the faces of 60 American male and female undergraduates (primarily Caucasian) who self-identified as Democrats or Republicans used in previous work (Rule and Ambady 2010).<br>Study 4 Baron-Cohen et al.'s (2001) RMET, Mini PONS (Rosenthal<br>et al. 1979)	Income as Objective SES indicator<br>MacArthur Scale of Subjective Social Status (Subjective SES) to measure their subjective<br>SES (Adler et al. 2000).	Study 2 Controlling for objective SES, and education, Subjective SES was **negatively** associated with RMET scores B = -.05, SE = .02, t(146) = -2.89, p = .004<br><br>Study 3 Controlling for objective SES, and education, Subjective SES was **negatively** associated with RMET scores (B = -.03, SE = .02, t(193) = -2.03, p = .04). Income was **positively** associated with RMET scores (B = .02, SE = .01, t(193) = 2.12, p = .04)<br><br>Study 4 subjective SES significantly negatively predicted categorization accuracy,<br>B = -.02, SE = .01, t(216) = -2.30, p = .02,<br>Mini PONS scores, B = -.03, SE = .01, t(197) = -2.64, p = .01, and RME performance, B = -.05, SE = .02, t(216) = -3.12, p = .002
Deveney, C. M., Chen, S. H., Wilmer, J. B., Zhao, V., Schmidt, H. B., & Germine, L. (2018). How generalizable is the inverse relationship between social class and emotion perception?. *PloS one*, *13*(10), e0205949. https://doi.org/10.1371/journal.pone.0205949	Study 1 (N = 179);<br>Study 2 (N = 5,187);<br>Study 3 (N = 2,564)<br>Study 4<br>3,859	*Study 1*<br>RMET (Baron-Cohen et al., 2001);<br>Vocabulary test (Richler et al., 2017)	*Study 1*<br>Subjective SC:<br>status manipulation condition;<br>participant ladder ranking<br>Objective SC:<br>highest level of education;<br>annual income	*Study 1*<br>Emotion identification correlated negatively with subjective social class (β = -0.15, 95% CI = [-0.28,-0.02]) and one of two objective social class measures (participant education β = -0.15, 95% CI = [-0.03,-0.01])

		Study 2<br>RMET (Baron-Cohen et al., 2001);<br>Vocabulary test (Richler et al., 2017)<br><br>*Study 3*<br>A multiracial emotion matching test<br><br>*Study 4*<br>The Queen Square Face Discrimination Test: Emotion and Identity Subtests (Garrido et al., 2009)	*Study 2*<br>family income and parental education questions; participants highest level of education<br><br>*Study 3*<br>participants highest level of education<br><br>*Study 4*<br>family income and parental education questions; participants highest level of education	*Study 2*<br>Complex emotion identification correlated non-significantly with participant education (β = 0.02, p = 0.25; 95% CI = [-0.01, 0.05], n = 2,726), positively with childhood family income (β = 0.03, 95% CI = [0.01,0.06], n = 4,312), and positively with parental education (β = 0.06, 95% CI = [0.04,0.09], n = 4,225)<br>*Study 3*<br>Basic emotion identification correlated positively with participant education (β = 0.05, 95% CI = [0.02, 0.09]), n = 2,564)<br>*Study 4*<br>Basic emotion discrimination correlated positively with participant education (β = 0.09, 95% CI = [0.05,0.13], n = 2,079), positively with parental education (β = 0.06, 95% CI = [0.02,0.09], n = 3,225), and non-significantly with childhood family income (β = 0.2, 95% CI = [0.01,0.07], n = 3,272)
Dietze, P., & Knowles, E. D. (2021). Social Class Predicts Emotion Perception and Perspective-Taking Performance in Adults. *Personality and Social Psychology Bulletin, 47*(1), 42-56. https://doi.org/10.1177/0146167220914116	Study 1A (N = 300<br>Study 1B (N = 451	*Study 1A and 1B*<br>RMET (Baron-Cohen et al., 2001)	*Study 1A and 1B*<br>“People talk about social classes such as the poor, the working class, the middle class, the upper-middle class, and the upper class. Which of these classes would you say you belong to?” (Jackman & Jackman, 1983)	*Study 1A*<br>r = −.169, p = .003, 95% confidence interval [CI] = [−0.277, −0.057]<br>*Study 1B*<br>r = −.250, p = 7.30 × 10−8, 95% CI = [−0.335, −0.162]

Schmalor, A., & Heine, S. J. (2022). Subjective Economic Inequality Decreases Emotional Intelligence, Especially for People of High Social Class. *Social Psychological and Personality Science*, *13*(2), 608-617. https://doi.org/10.1177/19485506211024024	Study 2a (N = 284)	RMET (Baron-Cohen et al., 2001)	Subjective SC: participants indicated their subjective SES (Adler et al., 2000) on a ladder with 10 rungs that indicated one's relative standing in society; participants indicated which of five social classes they thought they belonged to (i.e., poor, working class, middle class, upper middle class, and upper class; Jackman & Jackman, 1983	β = −3.11, p < .001, 95%CI = [−4.08, −2.13]
Monroy, M., Cowen, A. S., & Keltner, D. (2022). Intersectionality in emotion signaling and recognition: The influence of gender, ethnicity, and social class. *Emotion*, *22*(8), 1980–1988. https://doi.org/10.1037/emo0001082	N = 555	Full body expressions for 34 different emotions	MacArthur scale of subjective social status (Adler et al., 2000)	The social class of the encoder predicted overall reliability in emotion recognition, such that lower class individuals were more reliably judged in their full-body expression of 34 emotions (β = -.21, p = .007). Evidence was also found for the influence of social class upon our decoders' ability to recognize emotion (β = -.22, p , .001). In this analysis, lower class individuals proved to be better judges of others' full-body expressions of 34 emotion states
Kafetsios, K., & Hess, U. (2022). Personality and the accurate perception of facial emotion expressions: What is accuracy and how does it matter? *Emotion*, *22*(1), 100–114. https://doi.org/10.1037/emo0001034	Study 7 (N = 525)	ACE-Faces short version	MacArthur scale of Subjective Social Status (Adler et al., 2000)	ACE bias (but not accuracy) was a positive predictor of higher subjective social status (β = .15, t = 2.42, p , .01)
Brener, S. A., Frankenhuis, W. E., Young, E. S., & Ellis, B. J. (2024). Social class, sex, and the ability to recognize emotions: The main effect is in the interaction. *Personality and Social Psychology Bulletin*, *50*(8), 1197–1210. https://doi.org/10.1177/01461672231159775	Study 1 (N = 418); Study 2 (N = 745); Study 3 (N = 381)	RMET (Baron-Cohen et al., 2001); the visual portion of the CAM (Golan et al., 2006)	Two items for Subjective SC: "People talk about social classes such as the poor, the working class, the middle class, the upper-middle class, and the upper class. Which	Subjective social class was inversely related to performance on the RMET and the CAM; Multivariate analyses in Studies 1

			of these classes would you say you belong to?” (Jackman & Jackman, 1983); McArthur ladder (Adler et al., 2000)<br>Objective SC: average of mother and father education	and 2 revealed that (a) objective social class (i.e., parental education) did not significantly predict emotion recognition (though p = .073 in Study 2), and (b) subjective social class remained a significant predictor of emotion recognition after controlling for objective social class;<br>The association between SSC and emotion recognition occurred only in males
Engstrom, H. R., & Laurin, K. (2024). Lower social class, better social skills? A registered report testing diverging predictions from the rank and cultural approaches to social class. *Journal of Experimental Social Psychology, 111*, 1–15. https://doi.org/10.1016/j.jesp.2023.104577	N = 1139	RMET (Baron-Cohen et al., 2001);<br>The miniPONS (Bänziger et al., 2011)	Subjective SES: ladder with 10 rungs representing the socioeconomic hierarchy (Adler et al., 1994); participants indicated their social class category (poor / working class / middle class / upper-middle class / upper class; Dietze & Knowles, 2021).<br>Objective SES: highest educational degree; household income	Lower subjective rank, social class cultural group, and income—but not education—all predict better empathic accuracy;<br>subjective rank more strongly predicts empathic accuracy compared to SES cultural group (consistent with the rank approach), but childhood SES more strongly predicts empathic accuracy than adulthood SES (consistent with the cultural approach).

Table S4

Tucker's Phi Coefficients for Self-Construal Scale Factors across Countries

Country	Factor 1 (Independent SC)	Factor 2 (Interdependent SC)
China	.919	.962
Spain	.928	.954
Germany	.895	.947
Greece	.904	.928
India	.921	.848
Ireland	.953	.922
Italy	.970	.961
Japan	.955	.940
Poland	.922	.958
Turkey	.960	.966
USA	.965	.970
UK	.986	.986

Figure S1

Residuals versus predicted values plots of ACE accuracy

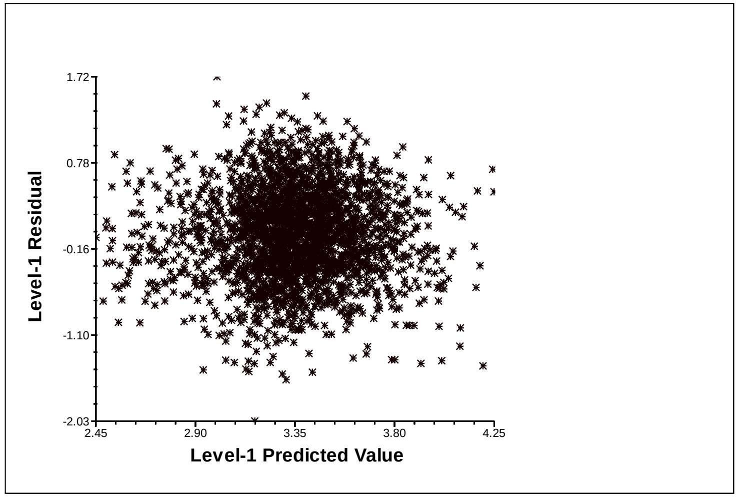

Figure S2

Residuals versus predicted values plots of ACE bias

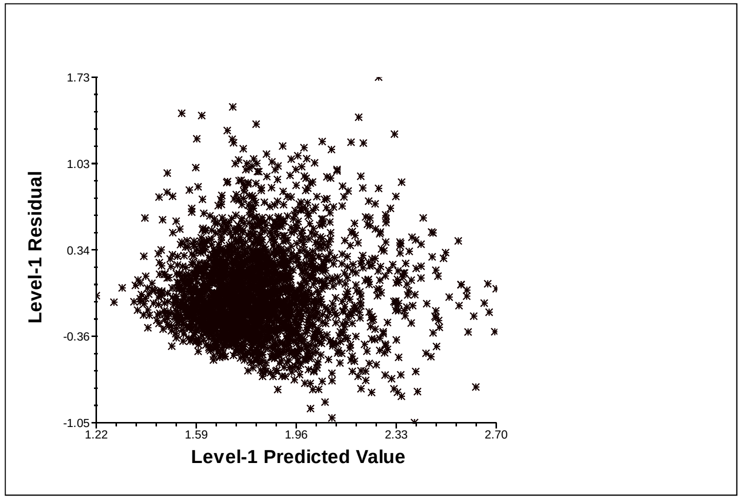

Fig. S3

Relationships between SSS and ACE accuracy

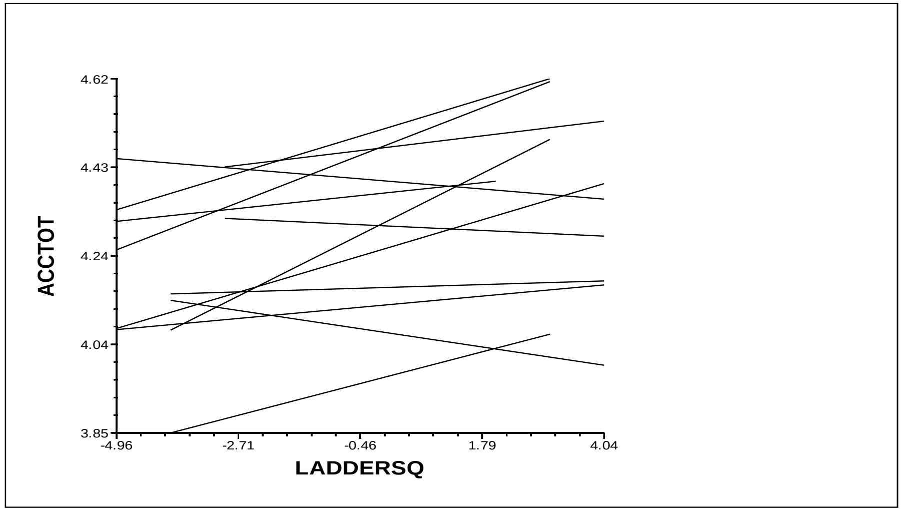

Fig. S4

Relationships between Parental Education Level and ACE bias

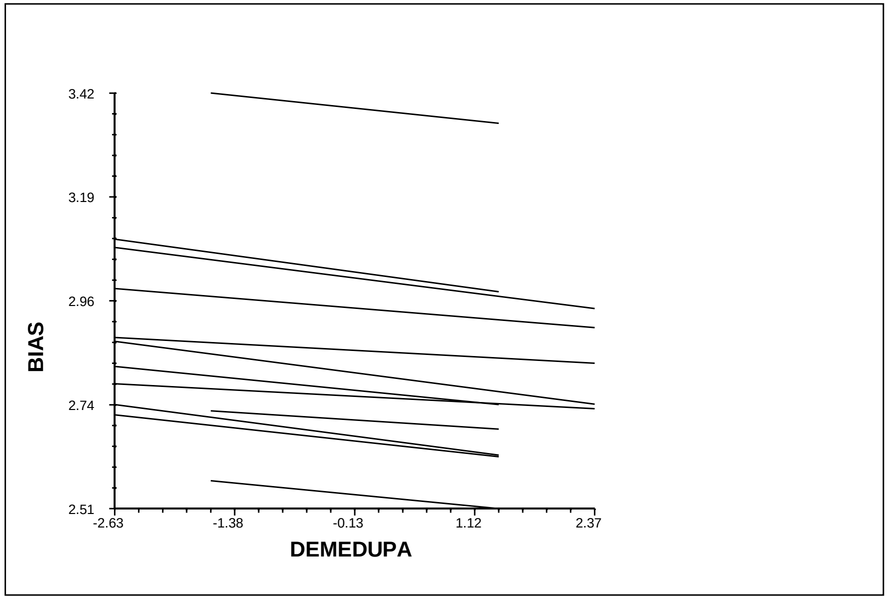

Table S5a (Accuracy – Happy)

Multilevel model of relationships between Subjective Social Status (SSS) and ACE accuracy happy

	Coef.	SE	t-value
Intercept γ_{00}	4.319	.079	54.54**
SSS γ_{10}	.019	.009	1.94^
Gender. γ_{20}	-.016	.06	-.267
Age γ_{30}	.004	.002	1.95^
Bias happy γ_{40}	-.278	.076	-3.636**

Note: Coefficients in bold are described in the results section. Gender coded -1 = males , 1 = females * *p* < .05, ** *p* < .01, *** *p* < .001, ^ < .08

Table S5b (Accuracy – Happy)

Multilevel model of relationships between Subjective Social Status (SSS) and ACE accuracy happy as a function of countries' Long Term Orientation (LTO), Relational Mobility (RM) and GINI

					**GINI**				**LTO**				**RM**		
	Coef.	SE	t-value		Coef.	SE	t-value		Coef.	SE	t-value		Coef.	SE	t-value
Intercept γ_{00}	4.323	.098	44.015***	γ_{01}	.0003	.0006	.96	γ_{02}	**-.007**	**.0001**	**-3.852*****	γ_{03}	-.059	.053	-1.113
SSS γ_{10}	.018	.012	1.502	γ_{11}	-.0001	.0001	-.771	γ_{12}	**.0001**	**.0003**	**4.506****	$\boldsymbol{\gamma_{13}}$	.0006	.012	.958
Gender. γ_{20}	-.018	.060	-.302												
Age γ_{30}	.004	.002	2.004^												
Bias happy γ_{40}	-.280	.080	-3.467**												

Note: Coefficients in bold are described in the results section. Gender coded -1 = males , 1 = females * *p* < .05, ** *p* < .01, *** *p* < .001, ^ < .08

Table S6a (Accuracy – Angry)

Multilevel model of relationships between Subjective Social Status (SSS) and ACE accuracy angry

	Coef.	SE	t-value
Intercept $_{\gamma 00}$	2.884	.086	33.245**
SSS $_{\gamma 10}$	.020	.01	1.968^
Gender. $_{\gamma 20}$	.144	.051	2.806*
Age $_{\gamma 30}$	-.008	.001	-4.534**
Bias angry $_{\gamma 40}$	.476	.052	9.040**

Note: Coefficients in bold are described in the results section. Gender coded -1 = males , 1 = females * *p* < .05, ** *p* < .01, *** *p* < .001, ^ < .08

Table S6b (Accuracy – Angry)

Multilevel model of relationships between Subjective Social Status (SSS) and ACE accuracy angry as a function of countries' Long Term Orientation (LTO), Relational Mobility (RM) and GINI

					GINI				LTO				RM		
	Coef.	SE	t-value		Coef.	SE	t-value		Coef.	SE	t-value		Coef.	SE	t-value
Intercept $_{\gamma 00}$	2.891	.126	22.855***	γ_{01}	**-.01**	**.00**	**-4.22****	γ_{02}	-.003	.002	-1.33	γ_{03}	**.340**	**.112**	**3.039***
SSS $_{\gamma 10}$	.021	.007	3.030*	γ_{11}	-.003	.001	-2.205^	γ_{12}	**.001**	**.0003**	**3.290***	γ_{13}	**.028**	**.009**	**2.974***
Gender. $_{\gamma 20}$	.140	.051	2.703*												
Age $_{\gamma 30}$	-.006	.051	-2.777*												
Bias angry $_{\gamma 40}$	.478	.052	9.075***												

Note: Coefficients in bold are described in the results section. Gender coded -1 = males . 1 = females * *p* < .05, ** *p* < .01, *** *p* < .001, ^ < .08

Table S7a (Accuracy – Sad)

Multilevel model of relationships between Subjective Social Status (SSS) and ACE sad accuracy

	Coef.	SE	t-value
Intercept γ_{00}	3.036	.066	45.802***
SSS γ_{10}	.021	.01	2.784*
Gender. γ_{20}	.149	.042	3.517**
Age γ_{30}	-.001	.001	-1.030
Bias sad γ_{40}	.443	.035	12.606***

Note: Coefficients in bold are described in the results section. Gender coded -1 = males , 1 = females * $p < .05$, ** $p < .01$, *** $p < .001$, ^ < .08

Table S7b (Accuracy – Sad)

Multilevel model of relationships between Subjective Social Status (SSS) and ACE sad accuracy as a function of countries' Long Term Orientation (LTO), Relational Mobility (RM) and GINI

					GINI				LTO				RM		
	Coef.	SE	t-value		Coef.	SE	t-value		Coef.	SE	t-value		Coef.	SE	t-value
Intercept γ_{00}	3.043	.071	42.488***	γ_{01}	-.05	.009	-1.709	γ_{02}	-.002	.001	-1.108	γ_{03}	**-.159**	**.053**	**-3.001***
SSS γ_{10}	.021	.007	2.720*	γ_{11}	-.000	.000	-.899	γ_{12}	.000	.000	.008	**γ_{13}**	**-.026**	**.008**	**-3.16***
Gender. γ_{20}	.145	.044	3.313**												
Age γ_{30}	-.001	.002	-1.010												
Bias sad γ_{40}	.447	.035	12.552***												

Note: Coefficients in bold are described in the results section. Gender coded -1 = males . 1 = females * $p < .05$, ** $p < .01$, *** $p < .001$, ^ < .08

Table S8a (Accuracy – Disgust)

Multilevel model of relationships between Subjective Social Status (SSS) and ACE accuracy disgust

	Coef.	SE	t-value
Intercept $_{\gamma 00}$	3.50	.074	47.017***
SSS $_{\gamma 10}$	.021	.008	2.347*
Gender. $_{\gamma 20}$	.106	.042	2.285*
Age $_{\gamma 30}$	-.014	.001	-6.235***
Bias disgust $_{\gamma 40}$	.231	.050	4.616***

Note: Coefficients in bold are described in the results section. Gender coded -1 = males , 1 = females * *p* < .05, ** *p* < .01, *** *p* < .001

Table S8b (Accuracy – Disgust)

Multilevel model of relationships between Subjective Social Status (SSS) and ACE accuracy disgust as a function of countries' Long Term Orientation (LTO), Relational Mobility (RM) and GINI

					**GINI**				**LTO**				**RM**		
	Coef.	SE	t-value		Coef.	SE	t-value		Coef.	SE	t-value		Coef.	SE	t-value
Intercept $_{\gamma 00}$	3.503	.082	42.215***	$\gamma 01$	**-.018**	**.007**	**-2.317***	$\gamma 02$	.001	.001	1.033	$\gamma 03$	**.148**	**.054**	**2.699***
SSS $_{\gamma 10}$	.021	.007	2.774*	$\gamma 11$	**-.004**	**.0009**	**-4.224****	$\gamma 12$	.0004	.0002	1.795	**$\gamma 13$**	.010	.010	.989
Gender. $_{\gamma 20}$	.105	.047	2.220*												
Age $_{\gamma 30}$	-.013	.002	-6.331***												
Bias disgust $_{\gamma 40}$	.232	.049	4.687***												

Note: Coefficients in bold are described in the results section. Gender coded -1 = males . 1 = females * *p* < .05, ** *p* < .01, *** *p* < .001

Table S9a (Accuracy – Congruent)

Multilevel model of relationships between Subjective Social Status (SSS) and ACE congruent accuracy

	Coef.	SE	t-value
Intercept γ_{00}	2.977	.085	34.910***
SSS γ_{10}	.021	.009	2.239*
Gender. γ_{20}	.187	.042	3.518**
Age γ_{30}	-.007	.001	-2.991*
Bias congruent γ_{40}	.545	.050	13.724***

Note: Coefficients in bold are described in the results section. Gender coded -1 = males , 1 = females * *p* < .05, ** *p* < .01, *** *p* < .001

Table S9b (Accuracy – Congruent)

Multilevel model of relationships between Subjective Social Status (SSS) and ACE congruent accuracy as a function of countries' Long Term Orientation (LTO), Relational Mobility (RM) and GINI

					**GINI**				**LTO**				**RM**		
	Coef.	SE	t-value		Coef.	SE	t-value		Coef.	SE	t-value		Coef.	SE	t-value
Intercept γ_{00}	2.981	.116	25.548***	γ_{01}	**-.027**	**.008**	**-3.301***	γ_{02}	**-.005**	**.001**	**-3.473****	γ_{03}	-.063	.065	-.968
SSS γ_{10}	.023	.005	4.473**	γ_{11}	-.001	.000	-1.509	γ_{12}	**.0008**	**.0001**	**5.761*****	**γ_{13}**	**-.016**	**.004**	**-3.653****
Gender. γ_{20}	.185	.053	3.466**												
Age γ_{30}	-.008	.001	-5.203**												
Bias congruent γ_{40}	.547	.040	13.653***												

Note: Coefficients in bold are described in the results section. Gender coded -1 = males . 1 = females * *p* < .05, ** *p* < .01, *** *p* < .001, ^ < .08

Table S10a (Accuracy – Incongruent)

Multilevel model of relationships between Subjective Social Status (SSS) and ACE incongruent accuracy

	Coef.	SE	t-value
Intercept γ_{00}	3.452	.036	95.354***
SSS γ_{10}	.012	.008	1.430
Gender. γ_{20}	.082	.041	2.012^
Age γ_{30}	-.001	.001	-1.540
Bias Incongruent γ_{40}	.466	.045	10.337***

Note: Coefficients in bold are described in the results section. Gender coded -1 = males , 1 = females * *p* < .05, ** *p* < .01, *** *p* < .001, ^ < .08

Table S10b (Accuracy – Incongruent)

Multilevel model of relationships between Subjective Social Status (SSS) and ACE congruent accuracy as a function of countries' Long Term Orientation (LTO), Relational Mobility (RM) and GINI

					**GINI**				**LTO**				**RM**		
	Coef.	SE	t-value		Coef.	SE	t-value		Coef.	SE	t-value		Coef.	SE	t-value
Intercept γ_{00}	3.456	.043	80.023***	γ_{01}	**-.012**	**.004**	**-2.594***	γ_{02}	-.0001	.001	-.073	γ_{03}	.03	.05	.61
SSS γ_{10}	.012	.008	1.572	γ_{11}	**-.003**	**.001**	**-3.158****	γ_{12}	.0004	.0002	1.577	γ_{13}	**-.018**	**.0007**	**-2.401***
Gender. γ_{20}	.080	.041	1.941												
Age γ_{30}	-.001	.0008	-1.751												
Bias Incongruent γ_{40}	.466	.044	10.375***												

Note: Coefficients in bold are described in the results section. Gender coded -1 = males . 1 = females * *p* < .05, ** *p* < .01, *** *p* < .001, ^ < .08

Table S11a

Multilevel model of relationships between Subjective Social Status (SSS) and ACE accuracy total

	Coef.	SE	t-value
Intercept γ_{00}	3.110	.065	47.243***
SSS γ_{10}	.017	.008	2.222*
Gender. γ_{20}	.149	.048	3.065*
Age γ_{30}	-.004	.001	-3.351*
Bias γ_{40}	.577	.037	15.245***

Note: Coefficients in bold are described in the results section. Gender coded -1 = males , 1 = females * $p < .05$, ** $p < .01$, *** $p < .001$

Table S11b [Table 3 in main MS]

Multilevel model of relationships between Subjective Social Status (SSS) and ACE accuracy as a function of countries' Long Term Orientation (LTO) and GINI

					GINI				LTO				RM		
	Coef.	SE	t-value		Coef.	SE	t-value		Coef.	SE	t-value		Coef.	SE	t-value
Intercept γ_{00}	3.118	.098	31.658***	γ_{01}	**-.023**	**.007**	**-3.131***	γ_{02}	**-.004**	**.001**	**-2.937***	γ_{03}	-.045	.067	-.677
SSS γ_{10}	.017	.006	2.633*	γ_{11}	**-.002**	**.0007**	**-3.398****	γ_{12}	**.0004**	**.0001**	**2.794***	γ_{13}	**-.023**	**.004**	**-4.828****
Gender. γ_{20}	.145	.049	2.959*												
Age γ_{30}	-.005	.001	-5.014**												
Bias γ_{40}	.579	.037	15.259***												

Note: Coefficients in bold are described in the results section. Gender coded -1 = males , 1 = females * $p < .05$ ** $p < .01$, *** $p < .001$ ^ < .08

Table S12a (Bias – Happy)

Multilevel model of relationships between Parental Education Level (PEL) and ACE bias happy

	Coef.	SE	t-value
Intercept γ_{00}	1.686	.040	41.429***
Parental Education Level γ_{10}	-.006	.002	-2.798*
Gender. γ_{20}	-.057	.013	-4.386**
Age γ_{30}	-.006	.002	-2.798*
ACCHAP γ_{40}	.293	.019	14.965***

Note: Coefficients in bold are described in the results section. Gender coded -1 = males , 1 = females * *p* < .05, ** *p* < .01, *** *p* < .001

Table S12b (Bias – Happy)

Multilevel model of relationships between Parental Education Level (PEL) and ACE bias happy as a function of countries' Long Term Orientation (LTO), Relational Mobility (RM) and GINI

					**GINI**				**LTO**				**RM**		
	Coef.	SE	t-value		Coef.	SE	t-value		Coef.	SE	t-value		Coef.	SE	t-value
Intercept γ_{00}	1.685	.029	56.934***	γ_{01}	-.006	.003	-2.124	γ_{02}	-.001	.0007	-1.84	γ_{03}	**-.231**	**.032**	**-7.213*** **
Gender. γ_{10}	-.057	.013	-4.399***												
Age γ_{20}	-.002	.001	-1.864												
Parental Education Level γ_{30}	-.006	.002	-2.801*	γ_{31}	.0007	.0003	-2.235^	γ_{32}	.000	.000	.603	**γ_{33}**	-.004	.0004	-.999
ACCHAP γ_{40}	-.080	.025	-3.117*												

Note: Coefficients in bold are described in the results section. Gender coded -1 = males , 1 = females * *p* < .05, ** *p* < .01, *** *p* < .001, ^ < .031

Table S13a (Bias – Angry)

Multilevel model of relationships between Parental Education Level (PEL) and ACE bias angry

	Coef.	SE	t-value
Intercept γ_{00}	1.686	.040	41.429***
Parental Education Level γ_{10}	-.006	.002	-2.798*
Gender. γ_{20}	-.057	.013	-4.386**
Age γ_{30}	-.006	.002	-2.798*
Accuracy Angry γ_{40}	.293	.019	14.965***

Note: Coefficients in bold are described in the results section. Gender coded -1 = males , 1 = females * p < .05, ** p < .01, *** p < .001

Table S13b (Bias – Angry)

Multilevel model of relationships between Parental Education Level (PEL) and ACE bias angry as a function of countries' Long Term Orientation (LTO), Relational Mobility (RM) and GINI

					GINI				LTO				RM		
	Coef.	SE	t-value		Coef.	SE	t-value		Coef.	SE	t-value		Coef.	SE	t-value
Intercept γ_{00}	2.144	.047	45.305***	γ_{01}	-.009	.004	-1.975	γ_{02}	**-.003**	**.001**	**-3.050***	γ_{03}	**-.162**	**.017**	**-9.452*** **
Gender. γ_{10}	-.098	.018	-5.436***												
Age γ_{20}	.001	.001	.793												
Parental Education Level γ_{30}	-.01	.004	-2.359*	γ_{31}	-.000	.000	-1.088	γ_{32}	.000	.000	.488	γ_{33}	.012	.012	1.022
Accuracy angry γ_{40}	.156	.021	7.158***												

Note: Coefficients in bold are described in the results section. Gender coded -1 = males , 1 = females * p < .05, ** p < .01, *** p < .001, ^ < .031

Table S14a (Bias – Sad)

Multilevel model of relationships between Parental Education Level (PEL) and ACE bias sad

	Coef.	SE	t-value
Intercept γ_{00}	1.686	.040	41.429***
Parental Education Level γ_{10}	-.006	.002	-2.798*
Gender. γ_{20}	-.057	.013	-4.386**
Age γ_{30}	-.006	.002	-2.798*
Accuracy sad γ_{40}	.293	.019	14.965***

Note: Coefficients in bold are described in the results section. Gender coded -1 = males , 1 = females * $p < .05$, ** $p < .01$, *** $p < .001$

Table S14b (Bias – Sad)

Multilevel model of relationships between Parental Education Level (PEL) and ACE bias sad as a function of countries' Long Term Orientation (LTO), Relational Mobility (RM) and GINI

					**GINI**				**LTO**				**RM**		
	Coef.	SE	t-value		Coef.	SE	t-value		Coef.	SE	t-value		Coef.	SE	t-value
Intercept γ_{00}	2.060	.059	34.810***	γ_{01}	-.003	.005	-.746	γ_{02}	-.001	.001	-.745	γ_{03}	**-.425**	**.036**	**-11.697*** **
Gender. γ_{10}	-.120	.018	-6.619***												
Age γ_{20}	.002	.002	1.083												
Parental Education Level γ_{30}	-.006	.005	-1.304	γ_{31}	.000	.000	-2.148^	γ_{32}	.000	.000	.075	**γ_{33}**	-.013	.010	-1.279
Accuracy sad γ_{40}	.149	.013	10.720***												

Note: Coefficients in bold are described in the results section. Gender coded -1 = males , 1 = females * $p < .05$, ** $p < .01$, *** $p < .001$, ^ < .031

Table S15a (Bias – Disgust)

Multilevel model of relationships between Parental Education Level (PEL) and ACE bias disgust

	Coef.	SE	t-value
Intercept γ_{00}	1.686	.040	41.429***
Parental Education Level γ_{10}	-.006	.002	-2.798*
Gender. γ_{20}	-.057	.013	-4.386**
Age γ_{30}	-.006	.002	-2.798*
Accuracy disgust γ_{40}	.293	.019	14.965***

Note: Coefficients in bold are described in the results section. Gender coded -1 = males , 1 = females * $p < .05$, ** $p < .01$, *** $p < .001$

Table S15b (Bias – Disgust)

Multilevel model of relationships between Parental Education Level (PEL) and ACE bias disgust as a function of countries' Long Term Orientation (LTO), Relational Mobility (RM) and GINI

					GINI				LTO				RM		
	Coef.	SE	t-value		Coef.	SE	t-value		Coef.	SE	t-value		Coef.	SE	t-value
Intercept γ_{00}	2.021	.052	38.242***	γ_{01}	-.008	.006	-1.230	γ_{02}	-.001	.001	-.099	γ_{03}	**-.283**	**.051**	**-5.513*** **
Gender. γ_{10}	-.128	.020	-6.64***												
Age γ_{20}	.002	.002	1.15												
Parental Education Level γ_{30}	-.013	.005	-2.48*	γ_{31}	.0002	.0008	-.243	γ_{32}	.0001	.0001	-1.102	γ_{33}	-.010	.009	-1.031
Accuracy disgust γ_{40}	.088	.017	5.014***												

Note: Coefficients in bold are described in the results section. Gender coded -1 = males , 1 = females * $p < .05$, ** $p < .01$, *** $p < .001$, ^ < .031

Table S16a (Bias – Congruent)

Multilevel model of relationships between Parental Education Level (PEL) and ACE bias congruent

	Coef.	SE	t-value
Intercept γ_{00}	1.686	.040	41.429***
Parental Education Level γ_{10}	-.006	.002	-2.798*
Gender. γ_{20}	-.057	.013	-4.386**
Age γ_{30}	-.006	.002	-2.798*
Accuracy congruent γ_{40}	.293	.019	14.965***

Note: Coefficients in bold are described in the results section. Gender coded -1 = males , 1 = females * $p < .05$, ** $p < .01$, *** $p < .001$

Table S16b (Bias – Congruent)

Multilevel model of relationships between Parental Education Level (PEL) and ACE bias congruent as a function of countries' Long Term Orientation (LTO), Relational Mobility (RM) and Gini

					GINI				LTO				RM	
	Coef.	SE	t-value		Coef.	SE	t-value		Coef.	SE	t-value		Coef.	SE	t-value
Intercept γ_{00}	2.060	.059	34.349***	γ_{01}	-.008	.002	-3.094	γ_{02}	-.001	.001	-1.012	γ_{03}	**-.121**	**.017**	**-6.973*****
Gender. γ_{10}	-.135	.021	-6.300***												
Age γ_{20}	.001	.001	.531												
Accuracy congruent γ_{30}	.255	.019	12.941***												
Parental Education Level γ_{40}	-.012	.004	-2.923*	γ_{41}	.0004	.0004	-.962	γ_{42}	**.0001**	**.004**	**-2.923***	γ_{43}	.0004	.0004	-.962

Note: Coefficients in bold are described in the results section. Gender coded -1 = males , 1 = females * $p < .05$, ** $p < .01$, *** $p < .001$, ^ < .031

Table S17a (Bias – Incongruent)

Multilevel model of relationships between Parental Education Level (PEL) and ACE bias incongruent

	Coef.	SE	t-value
Intercept γ_{00}	1.686	.040	41.429***
Parental Education Level γ_{10}	-.006	.002	-2.798*
Gender. γ_{20}	-.057	.013	-4.386**
Age γ_{30}	-.006	.002	-2.798*
Accuracy congruent γ_{40}	.293	.019	14.965***

Note: Coefficients in bold are described in the results section. Gender coded -1 = males , 1 = females * p < .05, ** p < .01, *** p < .001

Table S17b (Bias – Incongruent)

Multilevel model of relationships between Parental Education Level (PEL) and ACE bias incongruent as a function of countries' Long Term Orientation (LTO), Relational Mobility (RM) and Gini

					GINI				LTO				RM		
	Coef.	SE	t-value		Coef.	SE	t-value		Coef.	SE	t-value		Coef.	SE	t-value
Intercept γ_{00}	1.960	.037	52.244***	γ_{01}	-.005	.004	-1.27	γ_{02}	.001	.001	.725	γ_{03}	**-.335**	**.037**	**-8.907*** **
Gender. γ_{10}	-.080	.016	-4.756***												
Age γ_{20}	.001	.001	0.845												
Accuracy congruent γ_{40}	.211	.017	12.322***												
Parental Education Level γ_{40}	-.005	.004	-1.223	γ_{41}	**-.001**	**.0004**	**-3.362***	γ_{42}	.000	.004	-1.223	γ_{43}	**-.016**	**.005**	**-2.773***

Note: Coefficients in bold are described in the results section. Gender coded -1 = males , 1 = females * p < .05, ** p < .01, *** p < .001, ^ < .031

Table S18a

Multilevel model of relationships between Parental Education Level (PEL) and ACE bias

	Coef.	SE	t-value
Intercept γ_{00}	2.029	.055	36.421***
Gender. γ_{20}	-.115	.020	-5.751***
Age γ_{30}	.001	.001	.506
Accuracy γ_{40}	.293	.019	14.965***
Parental Education Level γ_{10}	**-.010**	**.004**	**-2.652***

Note: Coefficients in bold are described in the results section. Gender coded -1 = males , 1 = females * *p* < .05, ** *p* < .01, *** *p* < .001, ^ < .08

Table S18b

Multilevel model of relationships between Parental Education Level (PEL) and ACE bias as a function of countries' Long Term Orientation (LTO), Relational Mobility (RM) and GINI

					**Gini**				**LTO**				**RM**		
	Coef.	SE	t-value		Coef.	SE	t-value		Coef.	SE	t-value		Coef.	SE	t-value
Intercept γ_{00}	2.029	.049	40.885***	γ_{01}	**-.009**	**.003**	**-2.723***	γ_{02}	-.000	.001	-.298	γ_{03}	**-.125**	**.021**	**-5.813*****
Gender. γ_{10}	-.116	.020	-5.541***												
Age γ_{20}	.001	.001	0.576												
Accuracy γ_{30}	.293	.019	15.148***												
Parental Education Level γ_{40}	**-.01**	**.004**	**-2.619***	γ_{41}	.0008	.0005	-1.617	γ_{42}	-.0002	.004	-1.998^	γ_{43}	-.008	.008	-.949

Note: Coefficients in bold are described in the results section. Gender coded -1 = males , 1 = females * *p* < .05, ** *p* < .01, *** *p* < .001, ^ < .031

Table S19

Multilevel model of relationships between Subjective Social Status (SSS) and ACE accuracy as a function of countries' Long Term Orientation (LTO) and GINI

					GINI				LTO				RM		
	Coef.	SE	t-value		Coef.	SE	t-value		Coef.	SE	t-value		Coef.	SE	t-value
Intercept γ_{00}	3.118	.098	32.491***	γ_{01}	**-.023**	**.007**	**-3.092***	γ_{02}	**-.004**	**.001**	**-2.822***	γ_{03}	-.045	.067	-.677
SSS γ_{10}	.017	.006	2.635*	γ_{11}	**-.002**	**.0007**	**-3.398****	γ_{12}	**.0004**	**.0001**	**2.794***	γ_{13}	**-.023**	**.004**	**-4.828****
Gender. γ_{20}	.146	.048	2.9992*												
Age γ_{30}	-.005	.001	-4.969**												
Bias γ_{40}	.579	.037	15.279***												

Note: Coefficients in bold are described in the results section. Gender coded -1 = males , 1 = females * p < .05 ** p < .01, *** p < .001 ^ < .08

[Analyses excluding 12 cases where gender was coded as non-binary N = 2428]

Table S20

Multilevel model of relationships between Subjective Social Status (SSS) and ACE accuracy controlling for time elapsed since Covid-19

	Coef.	SE	t-value
Intercept γ_{00}	3.122	.070	44.079***
SSS γ_{10}	.018	.008	2.310*
Gender. γ_{20}	.139	.05	2.679*
Age γ_{30}	-.004	.001	-2.668**
Bias γ_{40}	.565	.039	14.174***
Covid γ_{50}	-.001	.0001	-.771

Note: Coefficients in bold are described in the results section. Gender coded -1 = males , 1 = females * p < .05 ** p < .01, *** p < .001

Table S21

Predicting ACE Accuracy Rates across each of the 12 cultures

	USA			Germany			Greece			UK			Spain			India		
	β	*t*	*p*	*β*	*t*	*p*	*β*	*t*	*p*	*β*	*t*	*p*	*β*	*t*	*p*	*β*	*t*	*p*
Model 1																		
Constant		9.10	.000		7.58	.000		9.59	.000		5.64	.000		6.04	.000		6.13	.000
Gender	0.22	3.66	.000	0.04	0.68	.500	-0.05	-0.85	.397	0.15	2.22	.027	0.24	3.39	.001	0.16	2.05	.042
SSS	-0.01	-0.11	.911	0.13	2.10	.037	0.16	2.48	.014	-0.01	-0.16	.873	-0.03	-0.41	.680	0.03	0.39	.694
Age	-0.15	-2.40	.017	-0.15	-2.32	.021	-0.10	-1.62	.107	-0.03	-0.50	.617	-0.01	-0.21	.837	0.01	0.13	.899
Bias	0.47	7.86	.000	0.43	6.94	.000	0.40	6.34	.000	0.38	5.74	.000	0.36	5.12	.000	0.36	4.55	.000
Model 2																		
Constant		8.68	.000		7.45	.000		9.35	.000		5.62	.000		6.13	.000		5.76	.000
Gender	0.23	3.44	.001	0.04	0.67	.503	-0.07	-1.10	.271	0.16	1.94	.053	0.24	3.46	.001	0.17	2.11	.036
SSS	-0.01	-0.14	.891	0.13	1.84	.067	0.20	2.86	.005	-0.02	-0.23	.818	-0.03	-0.42	.678	0.04	0.51	.608
Age	-0.15	-2.40	.017	-0.15	-2.32	.022	-0.11	-1.74	.084	-0.03	-0.50	.617	-0.01	-0.17	.863	0.01	0.13	.897
Bias	0.47	7.82	.000	0.43	6.87	.000	0.40	6.42	.000	0.38	5.70	.000	0.35	5.03	.000	0.36	4.57	.000
Gender x SSS	0.02	0.35	.723	0.00	0.02	.985	-0.10	-1.45	.149	0.02	0.17	.868	0.17	2.48	.014	-0.04	-0.54	.588
R^2	.26			.21			.21			.17			.18			.14		
F (5,145-228)	15.50			11.07			10.80			7.65			7.89			4.81		

Note. SSS = Subjective social status

Table S21 (continued)

	Ireland			Italy			Japan			Poland			Turkey			China		
	β	*t*	*P*	*β*	*t*	*P*	*β*	*t*	*p*	*β*	*T*	*p*	*β*	*t*	*p*	*β*	*t*	*p*
Model 1																		
Constant		8.52	.000		7.63	.000		3.59	.000		5.09	.000		2.78	.006		3.33	.001
Gender	0.31	3.97	.000	0.12	2.30	.022	0.06	1.03	.303	0.32	4.76	.000	0.09	1.47	.144	-0.22	-3.77	.000
SSS	-0.12	-1.55	.122	0.07	1.38	.168	0.14	2.21	.028	0.06	0.88	.381	0.01	0.18	.860	0.10	1.76	.081
Age	-0.05	-0.63	.528	-0.12	-2.33	.020	0.01	0.12	.908	-0.06	-0.95	.343	0.06	0.98	.328	0.09	1.61	.108
Bias	0.26	3.32	.001	0.43	8.07	.000	0.54	8.69	.000	0.33	4.90	.000	0.44	6.94	.000	0.51	9.02	.000
Model 2																		
Constant		8.13	.000		7.48	.000		3.67	.000		5.05	.000		2.67	.008		3.20	.002
Gender	0.31	4.01	.000	0.12	2.28	.024	0.04	0.61	.545	0.33	4.68	.000	0.09	1.32	.188	-0.23	-3.99	.000
SSS	-0.10	-1.19	.235	0.07	1.28	.201	0.13	2.10	.038	0.06	0.84	.401	0.01	0.17	.861	0.13	2.17	.032
Age	-0.05	-0.68	.497	-0.12	-2.33	.021	0.01	0.13	.898	-0.07	-0.95	.342	0.06	0.98	.330	0.09	1.64	.102
Bias	0.26	3.31	.001	0.43	8.01	.000	0.54	8.69	.000	0.33	4.89	.000	0.44	6.93	.000	0.52	9.12	.000
Gender x SSS	-0.06	-0.69	.493	0.00	-0.02	.987	0.06	0.82	.413	0.02	0.34	.732	0.00	-0.01	.989	-0.09	-1.51	.132
R^2	.17			.19			.32			.19			.20			.35		
F *(5,145-228)*	5.78			14.65			16.77			8.69			9.81			21.66		

Table S22

Predicting ACE bias rates across each of the 12 cultures

	USA			Germany			Greece			UK			Spain			India		
	β	*t*	*p*	*β*	*T*	*p*	*B*	*t*	*p*	*β*	*t*	*p*	*β*	*t*	*p*	*β*	*t*	*p*
Model 1																		
Constant		2.74	.007		4.14	.000		3.29	.001		3.41	.001		3.68	.000		5.48	.000
Gender	-0.17	-2.88	.004	-0.06	-0.92	.358	-0.03	-0.46	.649	-0.07	-1.01	.312	-0.25	-3.53	.001	-0.17	-2.21	.029
SSS	0.16	2.78	.006	-0.14	-2.21	.029	0.02	0.25	.800	0.04	0.63	.531	0.01	0.18	.858	-0.06	-0.80	.426
Age	0.18	2.99	.003	0.10	1.55	.123	0.09	1.35	.178	-0.02	-0.25	.805	0.00	0.07	.945	-0.18	-2.37	.019
Accuracy	0.46	7.86	.000	0.44	6.94	.000	0.41	6.34	.000	0.39	5.74	.000	0.36	5.12	.000	0.34	4.55	.000
Model 2																		
Constant		3.07	.002		3.76	.000		3.34	.001		3.45	.001		3.64	.000		5.61	.000
Gender	-0.23	-3.43	.001	-0.05	-0.77	.443	-0.01	-0.21	.834	-0.03	-0.37	.713	-0.25	-3.52	.001	-0.19	-2.44	.016
SSS	0.16	2.89	.004	-0.08	-1.13	.260	-0.02	-0.30	.767	-0.01	-0.12	.901	0.01	0.18	.858	-0.08	-1.06	.292
Age	0.18	2.99	.003	0.09	1.47	.143	0.09	1.46	.147	-0.02	-0.25	.804	0.00	0.07	.946	-0.18	-2.36	.020
Accuracy	0.45	7.82	.000	0.43	6.87	.000	0.41	6.42	.000	0.38	5.70	.000	0.36	5.03	.000	0.34	4.57	.000
Gender x SSS	-0.12	-1.91	.057	-0.12	-1.70	.090	0.09	1.24	.216	0.09	0.87	.387	-0.01	-0.11	.912	0.09	1.18	.238
R^2	.29			.21			.18			.15			.16			.18		
$F_{(5,145-228)}$	18.29			11.19			9.20			6.87			6.67			6.50		

Table S22(continued)

	Ireland			Italy			Japan			Poland			Turkey			China		
	β	*t*	*p*	*β*	*t*	*p*	*β*	*t*	*p*	*β*	*t*	*p*	*β*	*t*	*p*	*B*	*t*	*p*
Model 1																		
Constant		4.14	.000		6.64	.000		3.52	.001		5.74	.000		4.70	.000		2.26	.025
Gender	-0.17	-2.04	.043	-0.21	-4.09	.000	-0.13	-2.13	.034	-0.24	-3.30	.001	-0.13	-2.11	.036	0.06	1.01	.312
SSS	0.08	0.98	.328	0.03	0.55	.580	-0.04	-0.62	.536	-0.05	-0.67	.501	0.01	0.17	.864	-0.08	-1.32	.189
Age	-0.01	-0.10	.921	0.08	1.57	.118	0.05	0.82	.413	0.14	2.04	.043	-0.05	-0.80	.423	-0.03	-0.57	.572
Accuracy	0.28	3.32	.001	0.42	8.07	.000	0.54	8.69	.000	0.34	4.90	.000	0.44	6.94	.000	0.55	9.02	.000
Model 2																		
Constant		4.11	.000		6.13	.000		3.35	.001		5.72	.000		4.56	.000		2.34	.020
Gender	-0.17	-2.04	.043	-0.19	-3.78	.000	-0.12	-1.75	.082	-0.23	-3.16	.002	-0.14	-1.95	.053	0.08	1.30	.194
SSS	0.08	0.85	.398	0.06	1.15	.250	-0.04	-0.57	.572	-0.05	-0.68	.499	0.01	0.13	.893	-0.11	-1.79	.075
Age	-0.01	-0.08	.933	0.08	1.50	.133	0.05	0.81	.418	0.14	2.03	.043	-0.05	-0.81	.420	-0.04	-0.61	.543
Accuracy	0.28	3.31	.001	0.41	8.01	.000	0.54	8.69	.000	0.34	4.89	.000	0.44	6.93	.000	0.56	9.12	.000
Gender x SSS	0.02	0.18	.855	-0.09	-1.68	.094	-0.03	-0.45	.650	0.01	0.09	.927	0.01	0.13	.897	0.10	1.62	.107
R^2	.08			.22			.31			.15			.20			.30		
$F_{(5,145-228)}$	2.52			17.19			16.45			6.82			10.31			17.42		

Note. SSS = Subjective social status

Table S23

Predicting Hit Rates in the US

	β	*t*	*p*
Model 1			
Constant		7.96	.000
Gender	0.04	0.63	.532
SSS	**-0.15**	**-2.33**	**.021**
Age	-0.13	-1.86	.065
Model 2			
Constant		7.57	.000
Gender	0.07	0.91	.365
SSS	**-0.15**	**-2.36**	**.019**
Age	-0.13	-1.85	.066
Gender x SSS	0.06	0.80	.423
R^2	.05		
F (3,229)	2.95		

Note: SSS = Subjective social status

Table S24

Predicting Single Faces Results from MRCM across the 12-culture sample

	Hit rates		ACE Accuracy		ACE Bias	
	Coef.	p-value	Coef.	p-value	Coef.	p-value
Constant	.579	.041***	4.43	.043***	1.668	.048***
Gender	.006	.018	.04	.913	-.061	.017**
SSS	.001	.004	**.026**	**.01***	.0002	.005
Age	-.001	.0009	-.0001	.0001	-.0001	.0001
ACE bias			-.05	.077		
ACE accuracy					-.030	.030

Note. Gender coded -1 = males, 1 = females * $p < .05$ ** $p < .01$, *** $p < .001$

Table 25

Zero order correlations for main study variables in each of the 12 cultures

	Germany 1	2	3	4	5	Greece 1	2	3	4	5	UK 1	2	3	4	5
1. Accuracy	1	,406**	,339**	,110	-,031	1	,407**	,182**	,179**	,081	1	,379**	,165*	,021	-,041
2. Bias		1	-,495**	-,113	-,057		1	-,523**	,098	-,076		1	-,607**	,044	,042
3. Hit Rate			1	,113	-,006			1	-,020	,146*			1	,022	,094
4. SSS				1	,201**				1	,120				1	,183*
5. Parental Education					1					1					1

	Italy 1	2	3	4	5	Japan 1	2	3	4	5	Poland 1	2	3	4	5
1. Accuracy	1	,402**	,296**	,098	,088	1	,539**	,134	,164*	,140	1	,263**	,455**	,066	,058
2. Bias		1	-,521**	,081	,031		1	-,506**	,045	,043		1	-,472**	-,024	-,081
3. Hit Rate			1	,082	,055			1	,035	,055			1	,069	,046
4. SSS				1	,252**				1	,207**				1	,210**
5. Parental Education					1					1					1

	Spain 1	2	3	4	5	India 1	2	3	4	5	Ireland				
1. Accuracy	1	,316**	,242**	-,008	-,002	1	,336**	,181*	-,007	,050	1	,223**	,250**	-,125	-,015
2. Bias		1	-,584**	-,018	-,130		1	-,632**	-,090	-,077		1	-,606**	,046	-,014
3. Hit Rate			1	,000	,053			1	,073	,071			1	,101	,024
4. SSS				1	,107				1	-,125				1	,091
5. Parental Education					1					1					1

	Turkey					USA					China				
1. Accuracy	1	2	3	4	5	1	2	3	4	5	1	2	3	4	5
2. Bias	1	,429**	,306**	,014	-,050	1	,414**	,111	,081	-,005	1	,529**	,235**	,039	-,010
3. Hit Rate		1	-,429**	,023	-,050		1	-,559**	,211**	,008		1	-,504**	-,045	-,048
4. SSS			1	,004	,015			1	-,163*	-,065			1	,007	,020
5. Parental Education				1	,209**				1	,076				1	,267**
					1					1					1
